# Distribution and Effects of Nonsense Polymorphisms in Human Genes

**DOI:** 10.1371/journal.pone.0003393

**Published:** 2008-10-14

**Authors:** Yumi Yamaguchi-Kabata, Makoto K. Shimada, Yosuke Hayakawa, Shinsei Minoshima, Ranajit Chakraborty, Takashi Gojobori, Tadashi Imanishi

**Affiliations:** 1 Biological Information Research Center, National Institute of Advanced Industrial Science and Technology, Tokyo, Japan; 2 Japan Biological Information Research Center, Japan Biological Informatics Consortium, Tokyo, Japan; 3 Hamamatsu University School of Medicine, Hamamatsu, Shizuoka, Japan; 4 Center for Genome Information, University of Cincinnati, Cincinnati, Ohio, United States of America; 5 Center for Information Biology and DNA Data Bank of Japan, National Institute of Genetics, Mishima, Shizuoka, Japan; University of Western Cape, South Africa

## Abstract

**Background:**

A great amount of data has been accumulated on genetic variations in the human genome, but we still do not know much about how the genetic variations affect gene function. In particular, little is known about the distribution of nonsense polymorphisms in human genes despite their drastic effects on gene products.

**Methodology/Principal Findings:**

To detect polymorphisms affecting gene function, we analyzed all publicly available polymorphisms in a database for single nucleotide polymorphisms (dbSNP build 125) located in the exons of 36,712 known and predicted protein-coding genes that were defined in an annotation project of all human genes and transcripts (H-InvDB ver3.8). We found a total of 252,555 single nucleotide polymorphisms (SNPs) and 8,479 insertion and deletions in the representative transcripts in these genes. The SNPs located in ORFs include 40,484 synonymous and 53,754 nonsynonymous SNPs, and 1,258 SNPs that were predicted to be nonsense SNPs or read-through SNPs. We estimated the density of nonsense SNPs to be 0.85×10^−3^ per site, which is lower than that of nonsynonymous SNPs (2.1×10^−3^ per site). On average, nonsense SNPs were located 250 codons upstream of the original termination codon, with the substitution occurring most frequently at the first codon position. Of the nonsense SNPs, 581 were predicted to cause nonsense-mediated decay (NMD) of transcripts that would prevent translation. We found that nonsense SNPs causing NMD were more common in genes involving kinase activity and transport. The remaining 602 nonsense SNPs are predicted to produce truncated polypeptides, with an average truncation of 75 amino acids. In addition, 110 read-through SNPs at termination codons were detected.

**Conclusion/Significance:**

Our comprehensive exploration of nonsense polymorphisms showed that nonsense SNPs exist at a lower density than nonsynonymous SNPs, suggesting that nonsense mutations have more severe effects than amino acid changes. The correspondence of nonsense SNPs to known pathological variants suggests that phenotypic effects of nonsense SNPs have been reported for only a small fraction of nonsense SNPs, and that nonsense SNPs causing NMD are more likely to be involved in phenotypic variations. These nonsense SNPs may include pathological variants that have not yet been reported. These data are available from Transcript View of H-InvDB and VarySysDB (http://h-invitational.jp/varygene/).

## Introduction

Genetic variations in the human genome are maintained by a balance of mutation, selection and random genetic drift. Some of the polymorphisms cause phenotypic variations and diseases. Therefore, many studies have attempted to identify causative variants of genetic diseases and the relationships between genetic variations and phenotypic effects. Genetic variations within linked loci are inherited to the same gamete. Based on the linkage of genetic variations, loci that contain disease-causing genes have been mapped by using polymorphic markers. At present, about 14 million clusters of genetic polymorphisms have been identified in the human genome [1]. On average, two haploid genomes are estimated to differ by one single nucleotide polymorphism (SNP) in every 1200–1500 bp [2]. SNPs have been recently used to conduct genome-wide association studies to find genomic regions that are susceptible to diseases and phenotypic variations [3], [4], [5], [6]. In this approach, usually, causative polymorphisms for diseases or phenotypic variations are identified after the identification of susceptible genomic regions by using SNP markers. Such SNPs are called landmark SNPs, and the indirect relationships between polymorphisms and phenotypic variations were examined to identify genomic regions where causative genes are located.

Another approach in finding pathological variants is to extract polymorphisms that alter amino acids in functional genes or affect gene expression or splicing, using a comprehensive set of functional elements of the human genome. Several studies have analyzed nonsynonymous SNPs to predict pathological variants [7], [8], [9], [10], [11], [12], [13], [14]. A large number of nonsynonymous SNPs also have been examined for associations with diseases[15], [16].

Although many pathological mutations have been identified [17], [18], the number of such variants is small compared to the number of known polymorphisms, and it is still unclear which polymorphisms have biological effects. In a study of consanguineous marriage [19], it was estimated that each person has deleterious alleles that are equivalent to a few lethal genes. Gene-centric SNP surveys have shown that the ratio of nonsynonymous to synonymous SNPs is significantly higher in the low frequency class than in the common frequency class [20], [21], [22]. These results suggest that a large fraction of the low frequency nonsynonymous SNPs are deleterious. To understand the molecular basis of the effects of human genetic variations on phenotypic variations, a prediction analysis of possible effects of polymorphisms on gene function in all human genes appears to be needed.

In this study, to detect polymorphisms affecting gene function, we analyzed all publicly available polymorphisms in the Single Nucleotide Polymorphism Database (dbSNP) (build 125) in the exons of all 36,712 protein-coding genes that were defined in an annotation project of all human genes and transcripts (H-InvDB ver3.8)[23], [24]. In summary with representative transcripts (one transcript from one gene), we detected 53,754 nonsynonymous SNPs and 1,417 SNPs causing changes between amino acids and stop codons. Among possible point mutations in ORFs, nonsense mutations cause the most drastic changes of gene products. In fact, several reports have shown that nonsense mutations cause genetic diseases [25], [26], [27], [28]. Truncation of a polypeptide by a premature termination codon causes a drastic change in the gene product. Furthermore, it is known that a nonsense mutation can cause decay of mRNA resulting in the absence of the gene product. This process, called ‘nonsense-mediated decay (NMD)’ limits the synthesis of abnormal proteins[29], [30], [31]. On the other hand, the loss of a termination codon in a transcript also appears to cause decay of mRNA (referred to as non-stop decay) and thus to prevent translation[32], [33]. In spite of the severe effects of nonsense mutations, the distribution of nonsense SNPs in human genes is little understood. In this study, we examined the density of nonsense SNPs in human genes, and showed that nonsense SNPs exist at a lower density than nonsynonymous SNPs, possibly due to the more severe effects of premature stop codons than amino acid changes. About a half of nonsense SNPs are predicted to cause NMD. The correspondence between known pathological variants and nonsense SNPs suggests that nonsense SNPs causing NMD are more likely to be involved in phenotypic variations.

## Results

### Selection and classification of polymorphisms in exon regions

**Figure 1:**
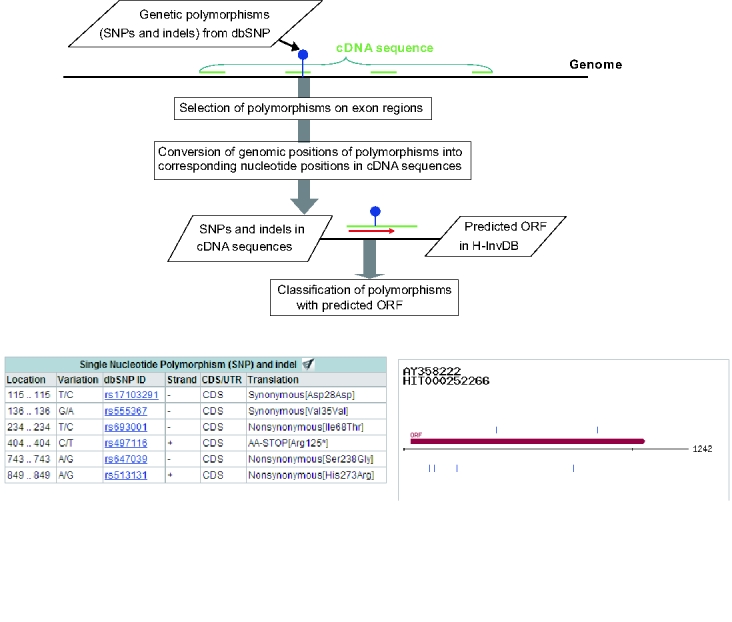
Analysis of polymorphisms with gene structure. Top: Scheme of analysis pipeline of polymorphisms with gene structure. Bottom: Screen shots taken from ‘Transcript View’ in H-InvDB that show classified SNPs and their positions (blue bars) in the *CASP12* gene.

We analyzed 9,235,997 polymorphisms (dbSNP build 125) in the human genome with exon positions and predicted ORFs that were revealed in our annotation project of human genes (H-InvDB) (Figure 1). In all of the 36,712 protein-coding loci in the genome, we detected 252,555 SNPs and 8,479 insertions and deletions (indels) that exist in exon regions of the representative transcript (one transcript from one gene) (Table 1). The polymorphisms in the exon regions were further classified according to the predicted ORFs. We detected 96,164 SNPs within the ORFs, 51,881 SNPs in the 5′UTR regions and 104,510 SNPs in the 3′UTR regions. Among the SNPs in the ORFs, 40,484 were synonymous and 53,754 were nonsynonymous (Further analyses of nonsynonymous SNPs are described in Results S1.). Most of the indels were detected in the UTR regions. The ORF regions contained 1,258 SNPs that cause changes between amino acids and stop codons (Table S1). Of the 1,258 SNPs, 1,183 SNPs were regarded as nonsense SNPs, while 75 were found to have stop codons as ancestral alleles. We also detected 247 SNPs at termination codon sites, 88 of which were synonymous. The remaining 159 SNPs were changes between stop codons and amino acids. After checking ancestral alleles, 110 of the 159 SNPs were inferred to be read-through SNPs, while the other 49 were inferred to changes to stop codons.

**Table 1:** SNPs and indels in exon, intron and other genomic regions.

	Exon	Intron	Other genomic regions
SNPs	249,182	3,332,537	5,209,127
Indels	9,742	185,761	249,648

Polymorphisms mapped on single positions were analyzed with 36,712 protein-coding genes.

### Distribution of polymorphisms in exon regions

Densities of polymorphisms were estimated for 23,717 genes whose functions are clearly defined or suggested (similarity category I–III, see Materials and Methods) and genes annotated as conserved hypothetical proteins (similarity category IV). To estimate the densities of SNPs for synonymous, nonsynonymous and nonsense SNPs in the ORFs, we calculated the numbers of potential nucleotide sites for synonymous, nonsynonymous and nonsense mutations in the coding regions. The fractions of sites (%) in the coding regions for synonymous, nonsynonymous, and nonsense mutations were estimated to be 28.5%, 68.1%, and 3.4%, respectively. Of the three types of SNPs, synonymous SNPs had the highest density, 4.1×10^−3^ per synonymous site, in ORFs (Table 2). The estimated density of nonsynonymous SNP was 2.1×10^−3^ per site (Table 2). The lower density of nonsynonymous SNPs compared with synonymous SNPs (51%) is due to the functional constraint of amino acid changes, and is in agreement with previous studies [20], [22], [34]. However, the ratio of the numbers of nonsynonymous SNPs to synonymous SNPs per site is higher in this study compared with previous studies (32–34%) [20], [21], [22], which they focused on specific populations. The higher ratio of nonsynonymous SNPs in this study may be due to the fact that our study is based on pooled data from various populations world wide. This study includes many nonsynonymous SNPs that exist in relatively lower frequencies and are likely to be more population-specific in comparison to synonymous SNPs [20].

**Table 2:** Classified SNPs in exon regions.

Region	Effects on translation	Genes in category I–IV^a^	All protein-coding genes^b^
5′UTR		23454 [3.3×10^−3^/site]^c^	51881
ORF	Total	85233 [2.7×10^−3^/site]	96164
	Synonymous	37484 [4.1×10^−3^ /site]	40484
	Nonsynonymous	46261 [2.1×10^−3^ /site]	53754
	AA↔Ter^d^	938	1258
	Unclassified^e^	398	421
Stop codon	Total	152	247
	Synonymous	63	88
	Ter↔AA^d^	89	159
3′UTR		69691 [3.3×10^−3^/site]	104510
Total		178378	252555

a Representative transcripts in 23,717 genes whose function were defined or suggested (similarity category I–III) and genes annotated as conserved hypothetical proteins (similarity category IV).

b Representative transcripts in all protein-coding genes (36,712) including genes in similarity category I–IV plus similarity category V–VII (hypothetical protein, hypothetical short protein, and pseudogene candidate, respectively).

c Densities of polymorphisms are shown in brackets as average number of polymorphisms per site. The average lengths of the 5′UTR, ORF and 3′UTR regions in 23717 genes were 303.9 bp, 1343.5 bp, and 877.6 bp, respectively. The densities of SNPs for synonymous, nonsynonymous and nonsense SNPs in ORFs were calculated based on the numbers of potential nucleotide sites for synonymous, nonsynonymous and nonsense mutations in coding regions. The density of nonsense SNPs is shown in Table 3.

d SNPs causing changes between amino acids and stop codons.

Among random nucleotide mutations in ORFs, 3.4% would be expected to be nonsense mutations; however, the distribution of nonsense SNPs has not been evaluated or reported. The density of nonsense SNPs was estimated to be 0.85×10^−3^ per site (Table 3), which is only 21% of the density of synonymous SNPs, and 40% of the density of nonsynonymous SNPs. The reason for the lowest density of nonsense SNPs may be that premature stop codons have more severe effects than amino acid changes.

**Table 3:** SNPs causing changes between amino acids and stop codons.

Region	Effects on translation	Genes in category I–IV^a^	All protein-coding genes^a^
ORF	Nonsense	910 [0.85×10^−3^/site]^d^	1183
	Read-through^b^	28	75
Stop codon	Read-through	67	110
	Nonsense^c^	22	49

a These two gene sets are the same as Table 2.

b Possible read-through SNPs in which alleles coding stop codons were ancestral type. This may be due to existence of shorter ORFs in the ancestral population.

c Possible nonsense SNPs in which alleles coding stop codons were derived alleles. This may be due to existence of longer ORFs in the ancestral population.

d The densities of nonsense SNPs in ORFs were calculated based on the numbers of potential nucleotide sites for nonsense mutations in coding regions.

In the exons of the 36,712 loci, 8479 indels were detected, and 1,532 of them were found in ORFs. Among the latter, 1,331 are expected to cause frame shifts, resulting in drastic changes of proteins. The density of indels in ORFs was much lower than in the UTR regions (Table 4). The lower density of indels in the 5′UTRs than in the 3′UTRs suggests that functional constraint for insertions and deletions is higher in the 5′UTR regions than in the 3′UTR regions.

**Table 4:** Insertions and deletions in exon regions.

	Genes in category I–IV^a^	All protein-coding genes^a^
5′UTR	785 [0.11×10^−3^]^b^	2005
ORF	1120 [0.035×10^−3^]	1532
3′UTR	3323 [0.16×10^−3^]	4942
Total	5225^c^	8479

a These two gene sets are the same as Table 2.

b Densities of polymorphisms are shown in brackets as average number of polymorphisms per site.

c Three indels were located on both of ORF and UTR.

### Nonsense SNPs

We examined the patterns and the positions of the nonsense SNPs. There are 23 possible ways to change codons into stop codons (nine, seven and seven for the first, second and third positions, respectively), and all 23 were found (Table 5). Nonsense SNPs were more frequent at the first codon position than at the second and third positions (p<0.005, chi-square test). The most frequent type of nonsense mutation is the change from CGA to TGA (Table 5), which is a transitional change at CpG mutation hotspots [35]. However, it is notable that there were frequent transversional mutations such as GAA to TAA and GAG to TAG. Our analyses of nonsense polymorphisms revealed that changes between hydrophilic amino acids and termination codons by nucleotide changes at the first codon positions were very frequent.

**Table 5:** Frequency of each type of codon change for nonsense SNPs.

	TAA		TAG		TGA		Total
	Aaa→Taa	33	Aag→Tag	31	Aga→Tga	20	
1st	**Caa→Taa**	**62**	**Cag→Tag**	**162**	**Cga→Tga**	**203**	748*
	Gaa→Taa	80	Gag→Tag	125	Gga→Tga	32	
	tCa→tAa	27	tCg→tAg	19	tCa→tGa	25	
2nd			**tGg→tAg**	**80**			200
	tTa→tAa	18	tTg→tAg	18	tTa→tGa	13	
	taC→taA	25	taC→taG	25	tgC→tgA	22	
3rd					**tgG→tgA**	**85**	235
	taT→taA	19	taT→taG	27	tgT→tgA	32	
Total		264		487		432	1183

Bold letters show nucleotide changes by transition.

* P<0.005 by chi-square test.

We examined the positions of 1,183 nonsense polymorphisms in the coding regions. On average, nonsense SNPs were located at 250 codons upstream of the original termination codons. To predict whether a nonsense mutation causes nonsense-mediated decay (NMD) of mRNA, we examined the locations of nonsense SNPs in the exon-intron structure of the genes (Table 6). As a result, of the 1183 nonsense SNPs, 581 were predicted to cause NMD, and thus to prevent translation. The other 602 cases of nonsense SNPs were predicted to result in truncated proteins. For the cases that truncated proteins are produced, the average truncation was estimated to be 75 amino acids.

**Table 6:** Nonsense SNPs and prediction of NMD.

	Predicted to cause NMD^a^	Not for NMD^b^	Total
Known pathological variants	8^c^	0	8
Other nonsense SNPs	573	602	1175
Total	581	602	1183

a This prediction is based on that mRNA would be destroyed if a stop codon occurs in the 5′ side of the boundary, which is 50–55 nucleotides upstream from the 3′ end of the second to last exon. Here, the nonsense SNPs located in the 5′ side of the boundary, which was set at 50 nucleotides upstream from the 3′ end of the second to last exon, were predicted to cause NMD.

b This number includes SNPs in genes consisting of only one exon.

c P = 0.0033 by Fisher's exact test.

To see which of these nonsense SNPs were known pathological mutations, we compared them with allelic variants in the Online Mendelian Inheritance in Man (OMIM) database. Only eight of 1,183 nonsense SNPs (rs17602729 in *AMPD1*, rs283413 in *ADH1C*, rs10250779 in *PGAM2*, rs17215500 in *KCNQ1*, rs497116 in *CASP12*, rs2228325 in *ACTN3*, rs3092891 in *RB1* and rs28989186 in *BUB1B*) matched the variants in the OMIM database that are known variants with phenotypic variations (Table 7). This low value suggests that the biological effects of most nonsense SNPs have not yet been reported. Interestingly, each of the eight cases that matched known pathological variants was predicted to cause NMD (Table 7).

**Table 7:** Nonsense SNPs with known pathological effects.

Acc#	Chr	Gene symbol	SNP	Variation	OMIM	Biological effects
M60092	1	*AMPD1*	rs17602729	Gln12Ter	102770	AMPD deficiency
M12272	4	*ADH1C*	rs283413	Gly78Ter	103730	Parkinson disease
BC073741	7	*PGAM2*	rs10250779	Trp78Ter	261670	Myopathy
AF000571	11	*KCNQ1*	rs17215500	Arg518Ter	607542	Long QT syndrome 1
AY358222	11	*CASP12*	rs497116	Arg125Ter	608633	Sepsis susceptibility
M86407	11	*ACTN3*	rs2228325	Arg577Ter	102574	Athletic performance
L41870	13	*RB1*	rs3092891	Arg445Ter	180200	Bilateral retinoblastoma
AF068760	15	*BUB1B*	rs28989186	Arg194Ter	602860	Premature chromatid separation trait and mosaic variegated aneuploidy syndrome

### SNPs that cause read-though of the original termination codon

Among the 247 SNPs at termination codon sites, 119 SNP-mRNA pairs were found to be read-through mutations. If the allele having the stop codon is the ancestral type, the SNP is regarded as a change causing elongation of the polypeptide. However, an extended polypeptide would be expected only if there is an additional termination codon downstream. For 108 SNP-mRNA pairs, an additional termination codon was found in the 3′UTR region. The average extension was estimated to be 29 amino acids. Interestingly, we found five SNP-mRNA pairs that have no stop codons in the 3′UTR at all (The remaining six SNP-mRNA pairs do not have 3′UTR regions). For example, the T-to-C substitution (rs15941) in the *DDR2* gene (X74764) is predicted to be a read-through mutation (from TAG to CGA), and the transcript has no other stop codon in the 3′UTR region. The frequency of this SNP is unknown (it is monomorphic in the four populations in HapMap project [4]). However, if this polymorphism really exists, transcripts having this read-through mutation would not produce a protein. Another example is the T-to-C substitution (rs17850833) in the *MFSD3* gene (CR620962), which causes a change from TGA to CGA resulting in a change to arginine.

### Functional bias of genes having nonsense SNPs

To see whether there is any functional bias in genes having nonsense SNPs, we examined the frequent biological terms in the genes having nonsense SNPs. We classified the genes having nonsense SNPs into two categories: genes with nonsense SNPs that are predicted to cause NMD and genes with nonsense SNPs that are not predicted to cause NMD. For genes having nonsense SNPs that would cause NMD (Table 8), the molecular functions that are most overrepresented included phosphorylation, ATP binding, iron/calcium ion binding, nucleotide/RNA binding and transporter activity. The localization of these genes was also biased to the cell membrane and the proteinaceous extracellular matrix. On the other hand, the genes having nonsense SNPs predicted to not cause NMD showed less bias in biological function (Table 9).

**Table 8:** Functional bias of genes having nonsense SNPs causing NMD.

Top level	Gene Ontology no.	Gene Ontology	Observed gene no.^a^	Expected gene no.^b^	Ratio of enrichment	P value^c^
Biological process	0006118	electron transport	15	4.23	3.55	5.03×10^−5^
	0006468	protein amino acid phosphorylation	16	7.28	2.20	4.98×10^−3^
Cellular component	0016020	membrane	41	22.55	1.82	5.57×10^−4^
	0005578	proteinaceous extracellular matrix	8	1.21	6.62	2.17×10^−6^
Molecular function	0005524	ATP binding	35	17.15	2.04	1.79×10^−4^
	0004713	protein tyrosine kinase activity	16	6.46	2.48	1.56×10^−3^
	0004674	protein serine/threonine kinase activity	16	6.78	2.36	2.51×10^−3^
	0000166	nucleotide binding	14	5.61	2.50	2.79×10^−3^
	0004672	protein kinase activity	16	7.15	2.24	4.21×10^−3^
	0003723	RNA binding	10	3.11	3.22	1.82×10^−3^
	0005506	iron ion binding	8	2.00	4.00	1.32×10^−3^
	0005509	calcium ion binding	16	7.65	2.09	7.89×10^−3^
	0005215	transporter activity	10	3.44	2.91	3.76×10^−3^
	0016491	oxidoreductase activity	11	4.24	2.59	5.76×10^−3^
	0003779	actin binding	6	1.27	4.74	2.24×10^−3^
	0004759	carboxylesterase activity	5	0.24	20.44	4.19×10^−6^

a Number of genes with a molecular function in the 581 genes in which nonsense SNPs causing NMD were found.

b Expected number of genes that have a biological function in a sample of 581 genes, assuming a proportion of genes with a molecular function in all human genes.

c Enrichment of a biological term in the genes for nonsense SNPs was statistically evaluated as a upper probability in a hypergeometric distribution.

**Table 9:** Functional bias of genes having nonsense SNPs not causing NMD.

Top level	Gene Ontology no.	Gene Ontology	Observed gene no.^a^	Expected gene no.^b^	Ratio of enrichment	P value^c^
Biological process	0007156	homophilic cell adhesion	6	1.42	4.23	3.05×10^−3^
	0006310	DNA recombination	3	0.19	15.50	8.25×10^−4^
	0006414	translational elongation	3	0.34	8.85	4.48×10^−3^
	0042254	ribosome biogenesis and assembly	2	0.15	13.77	8.68×10^−3^
Cellular component	0005853	eukaryotic translation elongation factor 1 complex	2	0.13	15.50	6.82×10^−3^
Molecular function	0004194	pepsin A activity	2	0.18	11.27	1.30×10^−2^
	0003746	translation elongation factor activity	2	0.29	6.89	3.35×10^−2^

a Number of genes with a molecular function in the 602 genes in which nonsense SNPs causing NMD were found.

b Expected number of genes that have a biological function in a sample of 602 genes, assuming a proportion of genes with a molecular function in all human genes.

c Enrichment of a biological term in the genes for nonsense SNPs was statistically evaluated as a upper probability in a hypergeometric distribution.

## Discussion

In this study, we conducted an extensive analysis of human genome polymorphisms with a comprehensive catalogue of human genes, and detected more than 50,000 polymorphisms that affect proteins. The distribution of polymorphisms showed different densities of polymorphisms among the 5′UTR, ORF and 3′UTR. The density of SNPs was lower in ORFs than in the 5′UTR and 3′UTR. The density of synonymous SNPs in the ORFs was higher than the densities of SNPs in the UTR regions. The reduction in density of SNPs in the UTR regions is consistent that there are functional constraints on nucleotide changes in UTRs related to the transcriptional and translational efficiency[22]. The density of nonsynonymous SNPs was much lower than the densities of other types of SNPs, possibly due to that the nucleotide changes with alteration of amino acids changes are under strong negative selection [36]. It was not known how nonsense SNPs are distributed in protein-coding regions. Here we showed that the density of nonsense SNPs is much lower than that of nonsynonymous SNPs. Although the biological effects of nonsense mutations appear to vary widely depending on their positions and the genes, the low density of nonsense SNPs that we found suggests that nonsense mutations have more disadvantageous effects than nonsynonymous mutations.

While nonsense mutations that cause NMD result in ‘loss of function’, nonsense mutations that do not cause NMD produce truncated proteins which could have the dominant effects. The proportion of predicted nonsense SNPs causing NMD in this study is in agreement with a previous study which showed that dbSNP (build 125) has 1301 nonsense SNPs, about half of which were predicted to result in NMD [37]. In order to understand the biological effects of nonsense SNPs, it is important to know whether they do or do not cause NMD, because premature stop codons in a gene can have distinct disease phenotypes depending on the positions of mutations [27], [38].

The molecular functions that were overrepresented in the genes having nonsense SNPs included several molecular functions that were observed in human-specific pseudogenes[39], such as ATP binding, actin binding, calcium ion binding, extracellular matrix, nucleic acid binding and oxidoreductase. This is in accord with that nonsense mutations contribute to ‘pseudogenization’. It is interesting that nonsense SNPs causing NMD were frequently found in genes that encode proteins involved in phosphorylation, cell-cell interaction, signal transduction and transport. This may be because changes in the length of polypeptides caused by nonsense mutations are under strong negative selection in the genes involved in signal transduction or transportation because abnormal translation products could cause dominant effects. Therefore, inactivation of translation by nonsense mutations in those genes could have milder effects than changes of the length of polypeptides.

Our results showed a low proportion of matches of nonsense SNPs with known pathological variants in OMIM, suggesting that the effects of most nonsense polymorphisms are unknown or not reported. Furthermore, the correspondence of the nonsense SNPs to the OMIM allelic variants (Table 6, Table 7) suggests that nonsense polymorphisms that are subject to NMD are more likely to be involved in phenotypic variations.

There is a possibility that the nonsense SNPs detected here have pathological effects, in particular, if non-dispensable genes have nonsense mutations. First, a defect in one gene by a nonsense mutation or a frame-shifting indels causing a premature termination codon could be a cause of genetic diseases including complex diseases[40]. Second, there is a possibility that nonsense mutations cause recessive lethal alleles that would not be detected as causative variant of diseases. Probably, focusing on nonsense polymorphisms observed in specific populations would be a good way of selection for finding variants with deleterious effects.

The effect of single nonsense SNPs can be compensated by the products of other genes having similar functions[41] and the other splicing isoforms of the gene [42]. Thus, single nonsense SNPs may not always cause severe phenotypic effects. In fact, some nonsense SNPs with high allele frequencies were found across populations[43]. There is a report of fixation of an inactive form of caspase 12 by a nonsense mutation (rs497116) in non-African populations[43], and this is an example supporting the ‘less is more hypothesis’[44]. This example suggests that some of nonsense mutations are not disadvantageous and that the increase of frequency of a nonsense allele could be driven by positive selection.

Elongation of polypeptides by read-through mutations can affect protein folding and aggregation of proteins, which could affect phenotypic variations. Furthermore, a read-through mutation can cause more severe effects on translation when no additional stop codon follows. Such mutations are subject to ‘non-stop decay’ [32], [33], and would result in no gene product. It has been suggested that non-stop decay and NMD serve to remove toxic, aberrant proteins [29]. It is unclear how frequently such mutations prevent mRNA from producing proteins. Therefore, it would be quite useful to be able to predict the effects of various types of genetic changes on mRNA.

Although the present results are based on representative transcripts (one transcript for one gene), the total number of SNPs causing changes between amino acids and stop codons in all the splicing isoforms was much larger (2,234). These variations, which cause changes in the length of a polypeptide or which determine whether a protein is translated, may include pathological variants that have yet not been reported. Therefore, it is important to examine their presence in human populations.

## Materials and Methods

### Data of human genetic polymorphisms

As data of genetic polymorphisms of human genome, single nucleotide polymorphisms (SNPs) and insertions and deletions (indels) in dbSNP [1] were used in this study. The whole data of human SNPs and indels were downloaded from dbSNP (build 125). We used all SNPs and indels that were mapped on single position in the genome, except for ‘large insertions’ in dbSNP.

### Data of human genes

The data of human gene structure were obtained from H-InvDB ver3.8 (http://www.h-invitational.jp/), created by the annotation project of human genes (H-Invitational project) [23], [45]. Our analysis of all human genes that corresponds to H-InvDB (ver 3.8) predicted 36,712 protein coding loci. All protein-coding genes were annotated and classified based on similarity to known genes as follows; Category I, Identical to known human protein; Category II, Similar to known protein; Category III, IPR domain containing protein; Category IV, Conserved hypothetical protein; Category V, Hypothetical protein; Category VI, Hypothetical short protein; Category VII, pseudogene candidate. We used the following three kinds of data of the gene structure: 1) genomic location of exons to the human genome (build 35), 2) predicted ORF regions in transcripts, and 3) original and curated cDNA sequences.

### Analysis

#### 1. Analysis of polymorphism with exons and predicted ORFs

**Selection of polymorphisms on exon regions.** We selected polymorphisms in exon regions by comparing the genomic positions of polymorphisms and the start and end positions of exons that were obtained from mapping cDNA sequences to the human genome (Figure 1). Polymorphisms in introns were also selected in a same way.

**Conversion of genomic position of polymorphism into nucleotide position in cDNA sequence.** To analyze polymorphisms with a predicted ORF, nucleotide positions of polymorphisms in the human genome sequences were converted into the nucleotide positions in cDNA sequences. Because there could be gaps in the alignment of cDNA sequence and the human genome sequence, the nucleotide position was converted considering possible gaps in the alignment. When the cDNA sequence was corrected in ORF prediction because of frame-shifting and remaining intron, the nucleotide position of SNP was modified based on addition or deletion of nucleotides. For a quality control of polymorphism data used for classification, we conformed that one of the nucleotides in each pair of SNP alleles was the same nucleotide at the corresponding position in the cDNA sequence.

**Classification of polymorphisms with predicted ORF.** Polymorphisms within ORF were classified according to their effect on ORF. For SNPs with two alleles, alleles in nucleotide were converted into ‘alleles in codon’ by adding two other nucleotides in the codon from cDNA sequence. When a cDNA sequence was corrected in the annotation process by removing a remaining intron or by correcting a frameshift error, the corrected cDNA sequence was used. If these alleles in codon do not contain any stop codon, the alleles were classified into synonymous and nonsynonymous. In case a stop codon is included in the alleles in codon, they were classified into 1) premature termination (nonsense) codon, 2) read-through of original stop codon, and 3) synonymous at stop codon site, by assuming that the cDNA sequence has an ancestral allele. Indels were classified based on whether they are located in ORF. The indels within ORF were further classified by whether the insertion or deletion causes frame shifting in translation.

**Inference of direction of nonsense and read-through mutations.** Ancestral alleles were obtained from dbSNP (build 128) to check direction of mutations for SNPs causing changes between amino acids and stop codons. For nonsense SNPs in protein-coding regions, we checked whether the ancestral allele codes amino acids. In case that the ancestral allele codes stop codon, we do not regard this SNP as nonsense SNP, but is a read-through mutation assuming that there was a variant having a shorter ORF. For read-though SNPs at termination codon site, we checked whether the ancestral allele codes stop codon. In case that the ancestral allele codes amino acids, we regard this SNP not as a read-through mutation, but as a nonsense mutation in a variant having a longer ORF.

**Number of sites for synonymous, nonsynonymous and nonsense mutations.** To estimate densities of synonymous, nonsynonymous and nonsense SNPs, the numbers of potential synonymous, nonsynonymous and nonsense sites by single nucleotide changes were estimated for the ORF sequences. This is an extension of estimation of the numbers of synonymous and nonsynonymous sites[46]; the number of synonymous sites is calculated as the number of four-fold degenerate sites plus one-third of the number of two-fold degenerate sites. For 61 codons encoding amino acids, the numbers of nucleotide sites that would cause synonymous, nonsynonymous and nonsense mutations by a single nucleotide change were estimated with a model of nucleotide change. Here, the relative occurrence of a transitional mutation versus a transversional mutation (*r*) was set to be 4.0 (the expected ratio in the numbers of transitional and transversional mutations was 2.0). For example of the TTA codon for leucine, the number of nonsense sites was estimated to be 2.0/(*r*+2.0), because two types of transversional mutations at the second position cause nonsense mutations.

#### 2. Correspondence to known pathological variants

To check whether the polymorphisms that alter proteins are known pathological variants with phenotypic effect, we examined correspondence of SNPs with data of known pathological variants. We used data of ‘allelic variant’ in the Online Mendelian Inheritance in Man (OMIM) database [18] as information of variants with phenotypic effect. For nonsynonymous and nonsense SNPs, their effects on translation and positions in ORF were compared with the ‘list of alleles’ in OMIM (e.g. described as “TRP324TER” or “ALA279THR” for the *NGAS* gene).

#### 3. Prediction of nonsense SNPs causing NMD

Some of nonsense mutations cause nonsense-mediated decay (NMD), resulting in prevention of translation. It has been reported that mRNA would be destroyed if a stop codon occurs in the 5′ side of the boundary, which is 50–55 nucleotides upstream from the end of the second to last exon [30], [31]. To predict whether a nonsense SNP causes NMD, we examined whether a nonsense SNP is located in the 3′ side of the boundary, which was set at 50 nucleotides upstream from the end of the second to last exon, in the exon-intron structure. This method is the same as the method in SNP2NMD [37] when ‘NMD distance’ is 50 nucleotides.

#### 5. Functional bias of genes with nonsense SNPs

For each biological term from Gene Ontology (www.geneontology.org), a proportion of genes with the biological function in the genes having nonsense SNPs was compared with that in all human genes (representative transcripts in all human genes in H-InvDB ver 5.0), and the significance of over representation of a molecular function in the genes having nonsense SNPs was evaluated as the upper probability of the hypergeometric distribution.

## Supporting Information

Results S1 Supplementary results and a table for analyses of nonsynonymous SNPs.
(0.70 MB DOC)

Table S1 Nonsense SNPs and read-through SNPs on representative transcripts.
(4.24 MB DOC)
