# Supplementary material for: Distribution and Effects of Nonsense Polymorphisms in Human Genes
Source: PLoS One. 2008 Oct 14;3(10):e3393. doi: 10.1371/journal.pone.0003393 (PMC2561068; doi:10.1371/journal.pone.0003393)
Supplement: Results S1 — Supplementary results and a table for analyses of nonsynonymous SNPs. (0.70 MB DOC) [file pone.0003393.s001.doc]

| **Supplementary Text for** |
| --- |
| **Distribution and Effects of Nonsense Polymorphisms in Human Genes** |
| Yumi Yamaguchi-Kabata, Makoto K. Shimada, Yosuke Hayakawa, Shinsei Minoshima, Ranajit Chakraborty,  Takashi Gojobori, and Tadashi Imanishi |

**Contents**

**I. Supplementary Results for Analyses of Nonsynonymous SNPs**

**II. Supplementary Table**

**Nonsynonymous SNPs affecting domain detection**

**I. Supplementary Results for analyses of nonsynonymous SNPs**

**Prediction of nonsynonymous SNPs that disrupts functional domains**

To predict how amino acid change affect protein function, we examined whether amino acid polymorphism affect detection of functional domain. First, original ORFs in cDNA sequences were examined for functional domain in Pfam using InterPro (www.ebi.ac.uk/interpro/). For each nonsynonymous SNP, modified ORF sequence with another amino acid was generated, and was examined for functional domains with InterPro. Presence and absence of functional domains were compared between original ORF sequence and mutated ORF.

Among 96,164 polymorphisms in ORFs, 53,754 were nonsynonymous SNPs. Based on allelic variants in the OMIM database, 913 of nonsynonymous SNPs are known variants with phenotypic effects, and for the remaining larger part of them, their effects on phenotype have not been reported. Of the nonsynonymous SNPs, 370 were predicted to change functional domains that were defined by InterProscan (II. Supplementary Table, below). These nonsynonymous SNPs are candidate SNPs that may cause functional changes of proteins. Among these SNPs affecting functional domains, five SNPs corresponded to the allelic variants in OMIM. For example, a SNP rs1065852 in *CYP2D6*, which corresponds to Pro34Ser polymorphism in *CYP2D6,* affects catalytic activity and thermal stability of the enzyme by the amino acid change at 34th position between proline and serine, and this is associated with debrisoquine poor metabolizer phenotype.

**II. Supplementary Table. Nonsynonymous SNPs affecting domain detection**

| **No.** | | **SNP ID** | | **Transcript** | | **Variation** | | **Domain ID** | | **Domain name** | |  |
| --- | --- | --- | --- | --- | --- | --- | --- | --- | --- | --- | --- | --- |
| 1 | | rs947345 | | AB011539 | | P499L | | IPR013111 | | EGF, extracellular | |  |
| 2 | | rs9833995 | | AB018262 | | R193C | | IPR001440 | | Tetratricopeptide TPR_1 | |  |
| 3 | | rs6006165 | | AB020652 | | P595S | | IPR010790 | | Protein of unknown function DUF1388 | |  |
| 4 | | rs13102250 | | AB020807 | | L105W | | IPR001611 | | Leucine-rich repeat | |  |
| 5 | | rs4566088 | | AB023145 | | R499T | | IPR001650 | | Helicase, C-terminal | |  |
| 6 | | rs2889109 | | AB023161 | | P1971L | | IPR011704 | | ATPase associated with various cellular activities | |  |
| 7 | | rs4546904 | | AB037857 | | S277T | | IPR013106 | | Immunoglobulin V-set | |  |
| 8 | | rs752138 | | AB047004 | | L766P | | IPR002126 | | Cadherin | |  |
| 9 | | rs7553800 | | AB049405 | | N228K | | IPR001611 | | Leucine-rich repeat | |  |
| 10 | | rs17114359 | | AB051811 | | N202D | | IPR013091 | | EGF calcium-binding | |  |
| 11 | | rs7306642 | | AB052958 | | P2039T | | IPR013111 | | EGF, extracellular | |  |
| 12 | | rs28940284 | | AB053323 | | H302Q | | IPR000719 | | Protein kinase | |  |
| 13 | | rs17352063 | | AB058751 | | V373A | | IPR001452 | | Src homology-3 | |  |
| 14 | | rs2348663 | | AB061683 | | P547L | | IPR000601 | | PKD | |  |
| 15 | | rs17131915 | | AB061683 | | D812N | | IPR002859 | | PKD/REJ-like protein | |  |
| 16 | | rs9977124 | | AB076353 | | P216S | | IPR002494 | | Keratin, high sulfur B2 protein | |  |
| 17 | | rs2020113 | | AB076364 | | S49T | | IPR002494 | | Keratin, high sulfur B2 protein | |  |
| 18 | | rs13226569 | | AB111888 | | A482T | | IPR002172 | | Low density lipoprotein-receptor, class A | |  |
| 19 | | rs17856912 | | AB160987 | | V2174G | | IPR001304 | | C-type lectin | |  |
| 20 | | rs11539417 | | AB191264 | | C1615G | | IPR006209 | | EGF-like | |  |
| **No.** | **SNP ID** | | **Transcript** | | **Variation** | | **Domain ID** | | **Domain name** | |  |  |
| 21 | rs7145775 | | AF017464 | | W12R | | IPR013106 | | Immunoglobulin V-set | |  |  |
| 22 | rs3748020 | | AF031174 | | R449C | | IPR013151 | | Immunoglobulin | |  |  |
| 23 | rs9905604 | | AF039571 | | W851R | | IPR003961 | | Fibronectin, type III | |  |  |
| 24 | rs3122723 | | AL136793 | | D59E | | IPR002110 | | Ankyrin | |  |  |
| 25 | rs3958222 | | AL832622 | | D179A | | IPR010630 | | Protein of unknown function DUF1220 | |  |  |
| 26 | rs12889670 | | AL832848 | | L442F | | IPR001440 | | Tetratricopeptide TPR_1 | |  |  |
| 27 | rs2235231 | | AL833520 | | C177R | | IPR007087 | | Zinc finger, C2H2-type | |  |  |
| 28 | rs2868049 | | AY095145 | | N17D | | IPR004020 | | Pyrin | |  |  |
| 29 | rs7038559 | | AY149237 | | H1031R | | IPR008160 | | Collagen triple helix repeat | |  |  |
| 30 | rs10404519 | | AY165863 | | E1850K | | IPR013091 | | EGF calcium-binding | |  |  |
| 31 | rs17521570 | | AY317139 | | A47T | | IPR002110 | | Ankyrin | |  |  |
| 32 | rs17153879 | | AY358154 | | P12L | | IPR003961 | | Fibronectin, type III | |  |  |
| 33 | rs9902553 | | AY386261 | | I632T | | IPR001611 | | Leucine-rich repeat | |  |  |
| 34 | rs11540195 | | BC000214 | | C286Y | | IPR001680 | | WD-40 repeat | |  |  |
| 35 | rs12854479 | | BC000730 | | I520S | | IPR000008 | | C2 | |  |  |
| 36 | rs11553051 | | BC002508 | | S228P | | IPR003697 | | Maf-like protein | |  |  |
| 37 | rs17856236 | | BC002997 | | N724Y | | IPR002015 | | Proteasome/cyclosome, regulatory subunit | |  |  |
| 38 | rs4008242 | | BC008500 | | C217G | | IPR001993 | | Mitochondrial substrate carrier | |  |  |
| 39 | rs2276650 | | BC008894 | | R140P | | IPR002913 | | Lipid-binding START | |  |  |
| 40 | rs17845138 | | BC008894 | | R140P | | IPR002913 | | Lipid-binding START | |  |  |

| **No.** | **SNP ID** | **Transcript** | **Variation** | **Domain ID** | **Domain name** |  |  |
| --- | --- | --- | --- | --- | --- | --- | --- |
| 41 | rs17856446 | BC008894 | R140P | IPR002913 | Lipid-binding START |  |  |
| 42 | rs17857941 | BC008894 | R140P | IPR002913 | Lipid-binding START |  |  |
| 43 | rs17858069 | BC008894 | R140P | IPR002913 | Lipid-binding START |  |  |
| 44 | rs17879503 | BC011447 | R210C | IPR003597 | Immunoglobulin C1 type |  |  |
| 45 | rs17214044 | BC011447 | R210C | IPR003597 | Immunoglobulin C1 type |  |  |
| 46 | rs1042059 | BC011652 | C18Y | IPR001841 | Zinc finger, RING-type |  |  |
| 47 | rs11524166 | BC011872 | V185G | IPR002048 | Calcium-binding EF-hand |  |  |
| 48 | rs5272 | BC013734 | E488G | IPR002007 | Animal haem peroxidase |  |  |
| 49 | rs17855556 | BC014374 | F116V | IPR000276 | Rhodopsin-like GPCR superfamily |  |  |
| 50 | rs13432372 | BC014374 | P177A | IPR000276 | Rhodopsin-like GPCR superfamily |  |  |
| 51 | rs28942083 | BC014514 | C667Y | IPR006209 | EGF-like |  |  |
| 52 | rs2569548 | BC014514 | P685S | IPR006209 | EGF-like |  |  |
| 53 | rs28942084 | BC014514 | P685L | IPR006209 | EGF-like |  |  |
| 54 | rs13000235 | BC015353 | C30W | IPR001841 | Zinc finger, RING-type |  |  |
| 55 | rs2234942 | BC016705 | R28L | IPR006671 | Cyclin, N-terminal |  |  |
| 56 | rs12273710 | BC017238 | S297N | IPR001440 | Tetratricopeptide TPR_1 |  |  |
| 57 | rs17812699 | BC020551 | P138L | IPR008160 | Collagen triple helix repeat |  |  |
| 58 | rs6787209 | BC021290 | T126P | IPR000504 | RNA-binding region RNP-1 |  |  |
| 59 | rs2839718 | BC022318 | P122A | IPR008160 | Collagen triple helix repeat |  |  |
| 60 | rs3135509 | BC022318 | P122A | IPR008160 | Collagen triple helix repeat |  |  |
| **No.** | **SNP ID** | **Transcript** | **Variation** | **Domain ID** | **Domain name** |  |  |
| 61 | rs17878336 | BC022318 | L123V | IPR008160 | Collagen triple helix repeat |  |  |
| 62 | rs12036186 | BC022375 | Q330E | IPR002048 | Calcium-binding EF-hand |  |  |
| 63 | rs28399430 | BC023019 | P492R | IPR001128 | Cytochrome P450 |  |  |
| 64 | rs1049624 | BC027592 | K822N | IPR008144 | Guanylate kinase |  |  |
| 65 | rs1049625 | BC027592 | N829D | IPR008144 | Guanylate kinase |  |  |
| 66 | rs6117050 | BC027720 | L148F | IPR001611 | Leucine-rich repeat |  |  |
| 67 | rs10306140 | BC029840 | R149L | IPR002007 | Animal haem peroxidase |  |  |
| 68 | rs1135866 | BC030958 | L213S | IPR001611 | Leucine-rich repeat |  |  |
| 69 | rs954440 | BC032839 | Q192H | IPR001440 | Tetratricopeptide TPR_1 |  |  |
| 70 | rs17850841 | BC034489 | H310P | IPR007087 | Zinc finger, C2H2-type |  |  |
| 71 | rs11549830 | BC035077 | R562W | IPR001214 | Nuclear protein SET |  |  |
| 72 | rs1062941 | BC035581 | H608Y | IPR007087 | Zinc finger, C2H2-type |  |  |
| 73 | rs1042559 | BC037268 | H172R | IPR002219 | Protein kinase C, phorbol ester/diacylglycerol binding |  |  |
| 74 | rs2232437 | BC041661 | H463Y | IPR007087 | Zinc finger, C2H2-type |  |  |
| 75 | rs3962575 | BC043211 | C759R | IPR000832 | GPCR, family 2, secretin-like |  |  |
| 76 | rs4151648 | BC043484 | R734C | IPR001254 | Peptidase S1 and S6, chymotrypsin/Hap |  |  |
| 77 | rs16975326 | BC044567 | G3R | IPR001849 | Pleckstrin-like |  |  |
| 78 | rs12502450 | BC051751 | I747N | IPR003959 | AAA ATPase, central region |  |  |
| 79 | rs1058697 | BC058285 | L205F | IPR013151 | Immunoglobulin |  |  |
| 80 | rs12413389 | BC062545 | A85S | IPR001763 | Rhodanese-like |  |  |
| **No.** | **SNP ID** | **Transcript** | **Variation** | **Domain ID** | **Domain name** | |  |
| 81 | rs323844 | BC067825 | C469G | IPR006652 | Kelch repeat | |  |
| 82 | rs2020916 | BC069244 | P314H | IPR001327 | Pyridine nucleotide-disulphide oxidoreductase | |  |
| 83 | rs8190997 | BC069244 | I261V | IPR001327 | Pyridine nucleotide-disulphide oxidoreductase | |  |
| 84 | rs8191004 | BC069244 | E297D | IPR001327 | Pyridine nucleotide-disulphide oxidoreductase | |  |
| 85 | rs11575797 | BX640730 | A1643S | IPR000900 | Nebulin | |  |
| 86 | rs1062962 | BX647300 | A251T | IPR001440 | Tetratricopeptide TPR_1 | |  |
| 87 | rs17302349 | BX648353 | Y781C | IPR001064 | Beta and gamma crystallin | |  |
| 88 | rs12773843 | BX648524 | C28Y | IPR000372 | Leucine-rich repeat, cysteine-rich flanking region | |  |
| 89 | rs17121745 | BX648851 | T691A | IPR001680 | WD-40 repeat | |  |
| 90 | rs28370891 | CR590236 | R50K | IPR002575 | Aminoglycoside phosphotransferase | |  |
| 91 | rs3748811 | CR590236 | A57V | IPR002575 | Aminoglycoside phosphotransferase | |  |
| 92 | rs13955 | CR590236 | L246P | IPR002575 | Aminoglycoside phosphotransferase | |  |
| 93 | rs17343090 | CR594136 | R113C | IPR005336 | Protein of unknown function UPF0041 | |  |
| 94 | rs17354836 | CR594136 | R113C | IPR005336 | Protein of unknown function UPF0041 | |  |
| 95 | rs2272902 | CR594348 | T86M | IPR002610 | Rhomboid-like protein | |  |
| 96 | rs13397243 | CR595024 | F73L | IPR001128 | Cytochrome P450 | |  |
| 97 | rs16827426 | CR595684 | G87E | IPR008160 | Collagen triple helix repeat | |  |
| 98 | rs3211003 | CR595684 | G87E | IPR008160 | Collagen triple helix repeat | |  |
| 99 | rs7022771 | CR601019 | H114P | IPR001680 | WD-40 repeat | |  |
| 100 | rs3794438 | CR604287 | H207Q | IPR002110 | Ankyrin | |  |

| **No.** | **SNP ID** | **Transcript** | **Variation** | **Domain ID** | **Domain name** |  | |
| --- | --- | --- | --- | --- | --- | --- | --- |
| 101 | rs28434537 | CR613724 | G74V | IPR002198 | Short-chain dehydrogenase/reductase SDR |  | |
| 102 | rs2228049 | CR614015 | N204D | IPR001611 | Leucine-rich repeat |  | |
| 103 | rs28605689 | CR614817 | L26P | IPR012599 | Peptidase C1, propeptide |  | |
| 104 | rs11490925 | CR614989 | G3S | IPR011759 | Cytochrome C oxidase subunit II |  | |
| 105 | rs17850593 | CR622747 | D84N | IPR003008 | Tubulin/FtsZ, GTPase |  | |
| 106 | rs2516542 | CR622747 | D84N | IPR003008 | Tubulin/FtsZ, GTPase |  | |
| 107 | rs28860303 | CR627143 | A194P | IPR000477 | RNA-directed DNA polymerase (Reverse transcriptase) |  | |
| 108 | rs13061862 | D45371 | G54V | IPR008160 | Collagen triple helix repeat |  | |
| 109 | rs238521 | D79989 | G768V | IPR002110 | Ankyrin |  | |
| 110 | rs238519 | D79989 | G791A | IPR002110 | Ankyrin |  | |
| 111 | rs13306487 | J02703 | R515Q | IPR006209 | EGF-like |  | |
| 112 | rs3026985 | AF062733 | S50R | IPR013106 | Immunoglobulin V-set |  | |
| 113 | rs16911114 | AF064105 | I302T | IPR000242 | Tyrosine specific protein phosphatase |  | |
| 114 | rs536234 | AF085243 | C1056G | IPR007087 | Zinc finger, C2H2-type |  | |
| 115 | rs6064629 | AF119843 | A174T | IPR000357 | HEAT |  | |
| 116 | rs11554575 | AF161482 | G59R | IPR010456 | Ribosomal L11 methyltransferase |  | |
| 117 | rs368468 | J03464 | V270I | IPR008160 | Collagen triple helix repeat |  | |
| 118 | rs17399833 | J03490 | K283Q | IPR001327 | Pyridine nucleotide-disulphide oxidoreductase |  | |
| 119 | rs11538949 | J03490 | C306G | IPR001327 | Pyridine nucleotide-disulphide oxidoreductase |  | |
| 120 | rs140597 | L13923 | D1113G | IPR013091 | EGF calcium-binding |  |  |

| **No.** | **SNP ID** | **Transcript** | **Variation** | **Domain ID** | **Domain name** |  | |
| --- | --- | --- | --- | --- | --- | --- | --- |
| 121 | rs140599 | L13923 | C1153Y | IPR013091 | EGF calcium-binding |  | |
| 122 | rs140627 | L13923 | C1672F | IPR013091 | EGF calcium-binding |  | |
| 123 | rs363852 | L13923 | C166R | IPR013111 | EGF, extracellular |  | |
| 124 | rs363805 | L13923 | C2053F | IPR013091 | EGF calcium-binding |  | |
| 125 | rs363853 | L13923 | C177R | IPR013111 | EGF, extracellular |  | |
| 126 | rs363821 | L13923 | C2170F | IPR013091 | EGF calcium-binding |  | |
| 127 | rs17886149 | L14848 | V165I | IPR001039 | MHC class I, alpha chain, alpha1 and alpha2 |  | |
| 128 | rs3819269 | L14848 | V165I | IPR001039 | MHC class I, alpha chain, alpha1 and alpha2 |  | |
| 129 | rs3819268 | L14848 | H179L | IPR001039 | MHC class I, alpha chain, alpha1 and alpha2 |  | |
| 130 | rs1131896 | L14848 | G198S | IPR001039 | MHC class I, alpha chain, alpha1 and alpha2 |  | |
| 131 | rs17884174 | L14848 | G198S | IPR001039 | MHC class I, alpha chain, alpha1 and alpha2 |  | |
| 132 | rs28933699 | L32137 | D471Y | IPR003367 | Thrombospondin type 3 repeat |  | |
| 133 | rs28936669 | L32137 | D473G | IPR003367 | Thrombospondin type 3 repeat |  | |
| 134 | rs17856428 | L34056 | S373A | IPR002126 | Cadherin |  | |
| 135 | rs35213 | L34056 | S373A | IPR002126 | Cadherin |  | |
| 136 | rs12629872 | L38608 | L315M | IPR013151 | Immunoglobulin |  | |
| 137 | rs11558040 | L77701 | P2L | IPR007745 | Cytochrome C oxidase copper chaperone |  | |
| 138 | rs28935204 | M14113 | G92V | IPR011707 | Multicopper oxidase, type 3 |  | |
| 139 | rs28935208 | M14113 | L117R | IPR011707 | Multicopper oxidase, type 3 |  | |
| 140 | rs13306334 | M14335 | L1397F | IPR009271 | Coagulation factor V LSPD |  |  |

| **No.** | **SNP ID** | **Transcript** | **Variation** | **Domain ID** | **Domain name** |  | |
| --- | --- | --- | --- | --- | --- | --- | --- |
| 161 | rs2290635 | X81479 | C296S | IPR013091 | EGF calcium-binding |  | |
| 162 | rs11570076 | X84075 | R382W | IPR013098 | Immunoglobulin I-set |  | |
| 163 | rs3729986 | X84075 | V158M | IPR013106 | Immunoglobulin V-set |  | |
| 164 | rs3729989 | X84075 | S236G | IPR013106 | Immunoglobulin V-set |  | |
| 165 | rs11542907 | X98253 | C299S | IPR001841 | Zinc finger, RING-type |  | |
| 166 | rs1056936 | Z21943 | H676Y | IPR007087 | Zinc finger, C2H2-type |  | |
| 167 | rs2274254 | Z33642 | R833H | IPR013151 | Immunoglobulin |  | |
| 168 | rs2232250 | AF172244 | R192Q | IPR007848 | Methyltransferase small |  | |
| 169 | rs2232251 | AF172244 | H200Q | IPR007848 | Methyltransferase small |  | |
| 170 | rs3748867 | AF176832 | R3865P | IPR006209 | EGF-like |  | |
| 171 | rs2161468 | AF177941 | R1042P | IPR008160 | Collagen triple helix repeat |  | |
| 172 | rs2287813 | AF177941 | R1207P | IPR008160 | Collagen triple helix repeat |  | |
| 173 | rs2860678 | AF217745 | T152A | IPR002126 | Cadherin |  | |
| 174 | rs11657747 | AF229060 | T878M | IPR001611 | Leucine-rich repeat |  | |
| 175 | rs2295774 | AF274863 | S332A | IPR001680 | WD-40 repeat |  | |
| 176 | rs545330 | AF277624 | C383Y | IPR007087 | Zinc finger, C2H2-type |  | |
| 177 | rs11466656 | AF296673 | R469G | IPR001611 | Leucine-rich repeat |  | |
| 178 | rs11466657 | AF296673 | I473T | IPR001611 | Leucine-rich repeat |  | |
| 179 | rs28938468 | AF359281 | A364E | IPR001440 | Tetratricopeptide TPR_1 |  | |
| 180 | rs11570814 | AF390028 | L671P | IPR000719 | Protein kinase |  | |
| **No.** | **SNP ID** | **Transcript** | **Variation** | **Domain ID** | **Domain name** |  |  |
| 181 | rs9464337 | AF414088 | A747D | IPR008160 | Collagen triple helix repeat |  |  |
| 182 | rs28654288 | AF434715 | R210G | IPR001611 | Leucine-rich repeat |  |  |
| 183 | rs12809625 | AF464877 | C49G | IPR002110 | Ankyrin |  |  |
| 184 | rs12811140 | AF464877 | H51L | IPR002110 | Ankyrin |  |  |
| 185 | rs3741280 | AF491813 | H232N | IPR013151 | Immunoglobulin |  |  |
| 186 | rs12933442 | AF497245 | W20G | IPR007884 | DREV methyltransferase |  |  |
| 187 | rs3993295 | AF512499 | F207L | IPR001440 | Tetratricopeptide TPR_1 |  |  |
| 188 | rs1132442 | AJ001402 | E1056K | IPR006208 | Cystine knot |  |  |
| 189 | rs1188729 | AJ002535 | S4642C | IPR013151 | Immunoglobulin |  |  |
| 190 | rs6958498 | AJ001057 | P540A | IPR013098 | Immunoglobulin I-set |  |  |
| 191 | rs10150925 | AJ249900 | V82M | IPR002350 | Proteinase inhibitor I1, Kazal |  |  |
| 192 | rs3750608 | AJ276171 | R127Q | IPR013517 | FG-GAP |  |  |
| 193 | rs2277800 | AJ277750 | L28F | IPR000449 | Ubiquitin-associated |  |  |
| 194 | rs16955859 | AK023970 | V33A | IPR001179 | Peptidylprolyl isomerase, FKBP-type |  |  |
| 195 | rs8069375 | AK023970 | T114I | IPR001179 | Peptidylprolyl isomerase, FKBP-type |  |  |
| 196 | rs17845301 | AK023986 | T102I | IPR011498 | Kelch |  |  |
| 197 | rs17858136 | AK023986 | T102I | IPR011498 | Kelch |  |  |
| 198 | rs2303771 | AK023986 | T102I | IPR011498 | Kelch |  |  |
| 199 | rs6128739 | AK055202 | L796F | IPR000233 | Cadherin cytoplasmic region |  |  |
| 200 | rs9912347 | AK056005 | C176R | IPR007087 | Zinc finger, C2H2-type |  |  |

| **No.** | **SNP ID** | **Transcript** | **Variation** | **Domain ID** | **Domain name** |  |  |
| --- | --- | --- | --- | --- | --- | --- | --- |
| 201 | rs1875428 | AK056459 | I50M | IPR011511 | Variant SH3 |  |  |
| 202 | rs17844659 | AK056460 | A580V | IPR002126 | Cadherin |  |  |
| 203 | rs7083033 | AK074679 | H204R | IPR007087 | Zinc finger, C2H2-type |  |  |
| 204 | rs2231070 | AK090500 | P6A | IPR003597 | Immunoglobulin C1 type |  |  |
| 205 | rs2231072 | AK090500 | K8R | IPR003597 | Immunoglobulin C1 type |  |  |
| 206 | rs1059609 | AK090500 | S52L | IPR003597 | Immunoglobulin C1 type |  |  |
| 207 | rs2231086 | AK090500 | S52L | IPR003597 | Immunoglobulin C1 type |  |  |
| 208 | rs17839906 | AK090500 | S52L | IPR003597 | Immunoglobulin C1 type |  |  |
| 209 | rs17852200 | AK093145 | A302T | IPR001440 | Tetratricopeptide TPR_1 |  |  |
| 210 | rs10013280 | AK093145 | A302T | IPR001440 | Tetratricopeptide TPR_1 |  |  |
| 211 | rs17851955 | AK093163 | H193N | IPR007087 | Zinc finger, C2H2-type |  |  |
| 212 | rs10424525 | AK097729 | H364L | IPR007087 | Zinc finger, C2H2-type |  |  |
| 213 | rs10371 | AK122960 | A403T | IPR002913 | Lipid-binding START |  |  |
| 214 | rs6664730 | AK125663 | S1162L | IPR000357 | HEAT |  |  |
| 215 | rs8041035 | AK125721 | T781M | IPR000225 | Armadillo |  |  |
| 216 | rs951618 | AK125989 | I340T | IPR011545 | DEAD/DEAH box helicase, N-terminal |  |  |
| 217 | rs9511821 | AK127263 | T154S | IPR008250 | E1-E2 ATPase-associated region |  |  |
| 218 | rs2484842 | AK127436 | V420A | IPR000008 | C2 |  |  |
| 219 | rs3752137 | AK128857 | S1519R | IPR000082 | SEA |  |  |
| 220 | rs2976654 | AK131440 | C180W | IPR007087 | Zinc finger, C2H2-type |  |  |
| **No.** | **SNP ID** | **Transcript** | **Variation** | **Domain ID** | **Domain name** | |  |
| 221 | rs17853291 | AK172819 | V257G | IPR001124 | Lipid-binding serum glycoprotein | |  |
| 222 | rs28600289 | AF540980 | G33S | IPR001855 | Beta defensin | |  |
| 223 | rs2270856 | BC042133 | P297L | IPR000357 | HEAT | |  |
| 224 | rs11762417 | U87309 | C647R | IPR000547 | 7-Fold repeat in clathrin and VPS proteins | |  |
| 225 | rs7248295 | BC020512 | Y198S | IPR003140 | Phospholipase/Carboxylesterase | |  |
| 226 | rs17406621 | BC073834 | L82Q | IPR000504 | RNA-binding region RNP-1 | |  |
| 227 | rs7191155 | AF520570 | L220P | IPR000048 | IQ calmodulin-binding region | |  |
| 228 | rs11826453 | AF359415 | T30K | IPR000276 | Rhodopsin-like GPCR superfamily | |  |
| 229 | rs2560306 | AY461732 | R173H | IPR007087 | Zinc finger, C2H2-type | |  |
| 230 | rs16881817 | BC066301 | I210T | IPR013130 | Ferric reductase-like transmembrane component | |  |
| 231 | rs8139383 | BC073834 | L78M | IPR000504 | RNA-binding region RNP-1 | |  |
| 232 | rs7852218 | AK131322 | G56E | IPR008180 | DeoxyUTP pyrophosphatase | |  |
| 233 | rs11554413 | BC041414 | S70C | IPR013151 | Immunoglobulin | |  |
| 234 | rs3803764 | AB029041 | R285W | IPR000048 | IQ calmodulin-binding region | |  |
| 235 | rs11541234 | BC007840 | T119I | IPR001041 | Ferredoxin | |  |
| 236 | rs28942113 | AF537214 | D129E | IPR002048 | Calcium-binding EF-hand | |  |
| 237 | rs17406614 | BC073834 | R80C | IPR000504 | RNA-binding region RNP-1 | |  |
| 238 | rs16881812 | BC066301 | R166M | IPR013130 | Ferric reductase-like transmembrane component | |  |
| 239 | rs2241018 | BC036215 | D24A | IPR001993 | Mitochondrial substrate carrier | |  |
| 240 | rs28942114 | AF537214 | I136T | IPR002048 | Calcium-binding EF-hand | |  |

| **No.** | **SNP ID** | **Transcript** | **Variation** | **Domain ID** | **Domain name** |  |  |
| --- | --- | --- | --- | --- | --- | --- | --- |
| 241 | rs13409084 | BC014429 | G47S | IPR000369 | Slow voltage-gated potassium channel |  |  |
| 242 | rs10973199 | AK091718 | A8V | IPR009441 | Borna disease virus P40 |  |  |
| 243 | rs9437936 | BC020512 | T304R | IPR003140 | Phospholipase/Carboxylesterase |  |  |
| 244 | rs16932833 | AF309699 | C24Y | IPR000276 | Rhodopsin-like GPCR superfamily |  |  |
| 245 | rs17853496 | AB010438 | V43I | IPR011701 | Major facilitator superfamily MFS_1 |  |  |
| 246 | rs2288792 | AB011538 | R395Q | IPR001611 | Leucine-rich repeat |  |  |
| 247 | rs947345 | AB011539 | P499L | IPR002049 | EGF-like, laminin |  |  |
| 248 | rs2821008 | AB011539 | L600P | IPR002049 | EGF-like, laminin |  |  |
| 249 | rs13089536 | AB029018 | G140V | IPR001293 | Zinc finger, TRAF-type |  |  |
| 250 | rs6643791 | AB033032 | E1156D | IPR002909 | Cell surface receptor IPT/TIG |  |  |
| 251 | rs1060742 | AB037757 | Q18R | IPR001680 | WD-40 repeat |  |  |
| 252 | rs28756978 | AB039667 | R93G | IPR003594 | ATP-binding region, ATPase-like |  |  |
| 253 | rs5005333 | AB040024 | R59H | IPR001452 | Src homology-3 |  |  |
| 254 | rs17352063 | AB058751 | V373A | IPR011511 | Variant SH3 |  |  |
| 255 | rs13070515 | AB071037 | L286P | IPR001611 | Leucine-rich repeat |  |  |
| 256 | rs9980011 | AB076347 | S160P | IPR002494 | Keratin, high sulfur B2 protein |  |  |
| 257 | rs9980010 | AB076347 | S161P | IPR002494 | Keratin, high sulfur B2 protein |  |  |
| 258 | rs233317 | AB076347 | R241Q | IPR002494 | Keratin, high sulfur B2 protein |  |  |
| 259 | rs28644341 | AB076347 | C252Y | IPR002494 | Keratin, high sulfur B2 protein |  |  |
| 260 | rs10418391 | AF025530 | R208H | IPR013151 | Immunoglobulin |  |  |
| **No.** | **SNP ID** | **Transcript** | **Variation** | **Domain ID** | **Domain name** | |  |
| 261 | rs2228601 | AF026547 | A70T | IPR013106 | Immunoglobulin V-set | |  |
| 262 | rs13161411 | AF051782 | E755V | IPR005632 | Outer membrane chaperone Skp (OmpH) | |  |
| 263 | rs17855951 | AL138578 | C99G | IPR002641 | Patatin | |  |
| 264 | rs2076213 | AL138578 | C99G | IPR002641 | Patatin | |  |
| 265 | rs2076212 | AL138578 | G115C | IPR002641 | Patatin | |  |
| 266 | rs17855952 | AL138578 | I148M | IPR002641 | Patatin | |  |
| 267 | rs738409 | AL138578 | I148M | IPR002641 | Patatin | |  |
| 268 | rs7521022 | AL834196 | M2T | IPR012486 | N1221-like | |  |
| 269 | rs7951297 | AY026938 | R5C | IPR006038 | Uteroglobin superfamily | |  |
| 270 | rs6743376 | AY029413 | A51D | IPR000975 | Interleukin-1 | |  |
| 271 | rs6500304 | AY040220 | Y1013H | IPR001140 | ABC transporter, transmembrane region | |  |
| 272 | rs10964742 | AY139834 | E721K | IPR000357 | HEAT | |  |
| 273 | rs17852785 | AY298903 | P229T | IPR008539 | Protein of unknown function DUF821, CAP10-like | |  |
| 274 | rs7627615 | AY349352 | A71T | IPR006202 | Neurotransmitter-gated ion-channel ligand-binding | |  |
| 275 | rs13214568 | AY359119 | L63M | IPR001526 | CD59 antigen | |  |
| 276 | rs28362682 | AY684332 | W94R | IPR013106 | Immunoglobulin V-set | |  |
| 277 | rs4642671 | AY746432 | R1M | IPR002366 | Defensin propeptide | |  |
| 278 | rs837550 | BC002472 | M163I | IPR002123 | Phospholipid/glycerol acyltransferase | |  |
| 279 | rs17856373 | BC011722 | N374D | IPR012000 | Thiamine pyrophosphate enzyme, central region | |  |
| 280 | rs12273710 | BC017238 | S297N | IPR013105 | Tetratricopeptide TPR_2 | |  |

| **No.** | **SNP ID** | **Transcript** | **Variation** | **Domain ID** | **Domain name** |  |  |
| --- | --- | --- | --- | --- | --- | --- | --- |
| 281 | rs11547612 | BC031301 | P89H | IPR001611 | Leucine-rich repeat |  |  |
| 282 | rs1476703 | BC036045 | Y112N | IPR001357 | BRCT |  |  |
| 283 | rs11051523 | BC039107 | L195V | IPR010456 | Ribosomal L11 methyltransferase |  |  |
| 284 | rs10239197 | BC040594 | R527H | IPR007087 | Zinc finger, C2H2-type |  |  |
| 285 | rs1060877 | BC050340 | S310C | IPR007087 | Zinc finger, C2H2-type |  |  |
| 286 | rs2231924 | BC051316 | S46P | IPR009441 | Borna disease virus P40 |  |  |
| 287 | rs1057090 | BC056195 | A761V | IPR001357 | BRCT |  |  |
| 288 | rs4804664 | BC057831 | R193H | IPR007087 | Zinc finger, C2H2-type |  |  |
| 289 | rs404299 | BC064925 | A170T | IPR013151 | Immunoglobulin |  |  |
| 290 | rs2556480 | BC069087 | R353K | IPR001753 | Enoyl-CoA hydratase/isomerase |  |  |
| 291 | rs2556479 | BC069087 | D358N | IPR001753 | Enoyl-CoA hydratase/isomerase |  |  |
| 292 | rs11868727 | BX537955 | R278K | IPR002083 | MATH |  |  |
| 293 | rs3207945 | BX647769 | K34N | IPR002110 | Ankyrin |  |  |
| 294 | rs11556959 | BX648631 | H265R | IPR001012 | UBX |  |  |
| 295 | rs28370891 | CR590236 | R50K | IPR005581 | Fructosamine kinase |  |  |
| 296 | rs3748811 | CR590236 | A57V | IPR005581 | Fructosamine kinase |  |  |
| 297 | rs13955 | CR590236 | L246P | IPR005581 | Fructosamine kinase |  |  |
| 298 | rs17855477 | CR598763 | P42T | IPR013753 | Ras |  |  |
| 299 | rs11545400 | CR609307 | T62S | IPR002942 | RNA-binding S4 |  |  |
| 300 | rs13195509 | CR609907 | V55M | IPR013162 | CD80-like C2-set immunoglobulin |  |  |
| **No.** | **SNP ID** | **Transcript** | **Variation** | **Domain ID** | **Domain name** | |  |
| 301 | rs2258790 | CR618409 | Q5P | IPR003953 | Fumarate reductase/succinate dehydrogenase flavoprotein | |  |
| 302 | rs1051270 | D21267 | Q66H | IPR000727 | Target SNARE coiled-coil region | |  |
| 303 | rs1051276 | D21267 | T79K | IPR000727 | Target SNARE coiled-coil region | |  |
| 304 | rs2273426 | D38163 | A352G | IPR008160 | Collagen triple helix repeat | |  |
| 305 | rs13306487 | J02703 | R515Q | IPR013111 | EGF, extracellular | |  |
| 306 | rs12466181 | AF069307 | S283I | IPR001734 | Na+/solute symporter | |  |
| 307 | rs1275523 | AF069307 | I311T | IPR001734 | Na+/solute symporter | |  |
| 308 | rs2556480 | AF080598 | R353K | IPR001753 | Enoyl-CoA hydratase/isomerase | |  |
| 309 | rs2556479 | AF080598 | D358N | IPR001753 | Enoyl-CoA hydratase/isomerase | |  |
| 310 | rs17844498 | AF152501 | G569S | IPR002126 | Cadherin | |  |
| 311 | rs2860678 | AF152501 | A152T | IPR002126 | Cadherin | |  |
| 312 | rs17844484 | AF152501 | A152V | IPR002126 | Cadherin | |  |
| 313 | rs12976550 | L11672 | F520C | IPR007087 | Zinc finger, C2H2-type | |  |
| 314 | rs3732791 | L20469 | R327C | IPR000276 | Rhodopsin-like GPCR superfamily | |  |
| 315 | rs2075663 | L25286 | M204V | IPR012680 | Laminin G, subdomain 2 | |  |
| 316 | rs4742754 | L25286 | M204V | IPR012680 | Laminin G, subdomain 2 | |  |
| 317 | rs7869444 | L38929 | E1078D | IPR003961 | Fibronectin, type III | |  |
| 318 | rs11538762 | M13899 | P28H | IPR000817 | Prion protein | |  |
| 319 | rs2156928 | M77640 | Y1070D | IPR003961 | Fibronectin, type III | |  |
| 320 | rs4562591 | M85289 | V2074G | IPR013151 | Immunoglobulin | |  |
| **No.** | **SNP ID** | **Transcript** | **Variation** | **Domain ID** | **Domain name** | |  |
| 321 | rs755793 | M87770 | M205T | IPR013098 | Immunoglobulin I-set | |  |
| 322 | rs12722877 | M95610 | L321V | IPR008160 | Collagen triple helix repeat | |  |
| 323 | rs7533166 | U19769 | S2140L | IPR003900 | RepA / Rep+ protein KID | |  |
| 324 | rs10458342 | X53416 | R1959C | IPR001298 | Filamin/ABP280 repeat | |  |
| 325 | rs10030475 | X78416 | A117V | IPR001588 | Casein, alpha/beta | |  |
| 326 | rs11677877 | X80031 | H451R | IPR008160 | Collagen triple helix repeat | |  |
| 327 | rs11570076 | X84075 | R382W | IPR013151 | Immunoglobulin | |  |
| 328 | rs3729986 | X84075 | V158M | IPR013098 | Immunoglobulin I-set | |  |
| 329 | rs3729989 | X84075 | S236G | IPR013098 | Immunoglobulin I-set | |  |
| 330 | rs2466613 | X89669 | G146R | IPR000276 | Rhodopsin-like GPCR superfamily | |  |
| 331 | rs8104515 | X89673 | C14R | IPR000276 | Rhodopsin-like GPCR superfamily | |  |
| 332 | rs2274254 | Z33642 | R833H | IPR013106 | Immunoglobulin V-set | |  |
| 333 | rs11540480 | Z34289 | P16R | IPR013720 | LisH | |  |
| 334 | rs2232250 | AF172244 | R192Q | IPR013216 | Methyltransferase type 11 | |  |
| 335 | rs2232251 | AF172244 | H200Q | IPR013217 | Methyltransferase type 12 | |  |
| 336 | rs11539696 | AF209198 | V364M | IPR007087 | Zinc finger, C2H2-type | |  |
| 337 | rs17844469 | AF217750 | P575L | IPR002126 | Cadherin | |  |
| 338 | rs13189280 | AF217750 | P575L | IPR002126 | Cadherin | |  |
| 339 | rs4823561 | AF231024 | C1126R | IPR002126 | Cadherin | |  |
| 340 | rs3764656 | AF268610 | D353N | IPR012466 | Adaptin ear-binding coat-associated protein 1 NECAP-1 | |  |

| **No.** | **SNP ID** | **Transcript** | **Variation** | **Domain ID** | **Domain name** |  | |
| --- | --- | --- | --- | --- | --- | --- | --- |
| 341 | rs3743315 | AF322916 | I814T | IPR007596 | Viral A-type inclusion protein repeat |  | |
| 342 | rs221823 | AF332976 | F1096C | IPR003328 | Trypsin inhibitor-like, cysteine-rich TILa region |  | |
| 343 | rs3171439 | AF448439 | D632G | IPR004046 | Glutathione S-transferase, C-terminal |  | |
| 344 | rs3993298 | AF512499 | V124A | IPR001440 | Tetratricopeptide TPR_1 |  | |
| 345 | rs3993295 | AF512499 | F207L | IPR013105 | Tetratricopeptide TPR_2 |  | |
| 346 | rs1188729 | AJ002535 | S4642C | IPR013098 | Immunoglobulin I-set |  | |
| 347 | rs10150925 | AJ249900 | V82M | IPR011497 | Protease inhibitor, Kazal-type |  | |
| 348 | rs7280841 | AJ566387 | V206I | IPR002494 | Keratin, high sulfur B2 protein |  | |
| 349 | rs13046903 | AJ566389 | A16T | IPR007951 | PMG |  | |
| 350 | rs7275298 | AJ566389 | A16V | IPR007951 | PMG |  | |
| 351 | rs2274542 | AK021762 | E111K | IPR000980 | SH2 motif |  | |
| 352 | rs17159388 | AK022151 | R119Q | IPR001356 | Homeobox |  | |
| 353 | rs17845301 | AK023986 | T102I | IPR006652 | Kelch repeat |  | |
| 354 | rs17858136 | AK023986 | T102I | IPR006652 | Kelch repeat |  | |
| 355 | rs2303771 | AK023986 | T102I | IPR006652 | Kelch repeat |  | |
| 356 | rs17102066 | AK056186 | V504I | IPR004139 | Glycosyl transferase, family 13 |  | |
| 357 | rs1875428 | AK056459 | I50M | IPR001452 | Src homology-3 |  | |
| 358 | rs16845107 | AK056472 | K284N | IPR001680 | WD-40 repeat |  | |
| 359 | rs2258790 | AK074764 | Q5P | IPR003953 | Fumarate reductase/succinate dehydrogenase flavoprotein |  | |
| 360 | rs6767658 | AK092228 | T102A | IPR012619 | Myoactive tetradecapeptides |  |  |
| **No.** | **SNP ID** | **Transcript** | **Variation** | **Domain ID** | **Domain name** |  |  |
| 361 | rs16898958 | AK092633 | C1S | IPR001780 | Ribosomal protein L35Ae |  |  |
| 362 | rs6871453 | AK095496 | A526T | IPR002126 | Cadherin |  |  |
| 363 | rs1441618 | AK123850 | R188W | IPR013106 | Immunoglobulin V-set |  |  |
| 364 | rs1063792 | AK125568 | I355V | IPR013101 | Leucine-rich repeat 2 |  |  |
| 365 | rs12717784 | AK126106 | R40H | IPR000583 | Glutamine amidotransferase, class-II |  |  |
| 366 | rs2306265 | AK127168 | V529I | IPR001638 | Bacterial extracellular solute-binding protein, family 3 |  |  |
| 367 | rs3735786 | AK127625 | R227H | IPR007087 | Zinc finger, C2H2-type |  |  |
| 368 | rs3000859 | AK127828 | M1L | IPR013094 | Alpha/beta hydrolase fold-3 |  |  |
| 369 | rs8109273 | AK128700 | Y604H | IPR007087 | Zinc finger, C2H2-type |  |  |
| 370 | rs3895133 | AL049681 | F323V | IPR013101 | Leucine-rich repeat 2 |  |  |
