# Supplementary material for: Distribution and Effects of Nonsense Polymorphisms in Human Genes
Source: PLoS One. 2008 Oct 14;3(10):e3393. doi: 10.1371/journal.pone.0003393 (PMC2561068; doi:10.1371/journal.pone.0003393)
Supplement: Table S1 — Nonsense SNPs and read-through SNPs on representative transcripts. (4.24 MB DOC) [file pone.0003393.s002.doc]

**Table S1. Nonsense SNPs and read-through SNPs on representative transcripts**

| Chr | Position | SNP ID | Accession no. | H-Inv ID | cDNA Pos. | Codon change | AA change | Effect | Inferred direction | NMD |
| --- | --- | --- | --- | --- | --- | --- | --- | --- | --- | --- |
| 1 | 1315348 | rs12735892 | AF006011 | HIT000061809 | 905,- | tTg/tAg | L301* | PTC | NA | Yes |
| 1 | 1693116 | rs2377215 | BC073900 | HIT000265124 | 1010,- | tgA/tgG | *120W | Read-through | TER->AA | NA |
| 1 | 1919181 | rs28548017 | AB051538 | HIT000001467 | 2115,- | Cag/Tag | Q705* | PTC | AA->TER | No |
| 1 | 2105737 | rs3128302 | BC028413 | HIT000091597 | 3061,- | Gag/Tag | E104* | PTC | NA | No |
| 1 | 3342694 | rs12049194 | AB051462 | HIT000001391 | 891,+ | Gag/Tag | E287* | PTC | NA | Yes |
| 1 | 6409853 | rs1054273 | AK023754 | HIT000007028 | 290,- | tCg/tAg | S59* | PTC | TER->AA | No |
| 1 | 8859727 | rs11544512 | CR591792 | HIT000270987 | 898,+ | Gag/Tag | E250* | PTC | AA->TER | Yes |
| 1 | 11016513 | rs17849488 | BC071657 | HIT000264482 | 866,+ | Gaa/Taa | E261* | PTC | AA->TER | No |
| 1 | 11840334 | rs17413194 | BC005893 | HIT000032503 | 553,+ | Cga/Tga | R152* | PTC | NA | No |
| 1 | 13780214 | rs2495058 | BX538219 | HIT000055001 | 718,- | tCa/tAa | S34* | PTC | AA->TER | No |
| 1 | 14801945 | rs10927523 | AK000924 | HIT000003398 | 700,+ | tgG/tgA | W76* | PTC | AA->TER | No |
| 1 | 15260966 | rs12562474 | BX649098 | HIT000057745 | 795,+ | Cga/Tga | R94* | PTC | AA->TER | No |
| 1 | 15799590 | rs11557211 | BC040441 | HIT000096166 | 1947,+ | Gga/Tga | G573* | PTC | AA->TER | Yes |
| 1 | 16280690 | rs12048007 | BC040640 | HIT000052437 | 303,- | Gag/Tag | E56* | PTC | AA->TER | Yes |
| 1 | 16607415 | rs7551089 | AK125737 | HIT000045610 | 1375,- | Caa/Taa | Q135* | PTC | AA->TER | Yes |
| 1 | 16721459 | rs1057292 | BC044862 | HIT000097957 | 3757,+ | tgA/tgG | *126W | Read-through | NA | NA |
| 1 | 16768258 | rs557690 | AL035288 | HIT000250040 | 1912,- | Gag/Tag | E600* | PTC | TER->AA | No |
| 1 | 16829122 | rs11260920 | U28055 | HIT000218906 | 772,- | Cga/Tga | R258* | PTC | NA | No |

| Chr | Position | SNP ID | Accession no. | H-Inv ID | cDNA Pos. | Codon change | AA change | Effect | Inferred direction | NMD |
| --- | --- | --- | --- | --- | --- | --- | --- | --- | --- | --- |
| 1 | 20246888 | rs12139100 | ENST00000247992 | HIT000105820 | 67,- | Cga/Tga | R23* | PTC | AA->TER | No |
| 1 | 21980200 | rs11805009 | ENST00000334328 | HIT000128893 | 265,+ | Cga/Tga | R89* | PTC | AA->TER | No |
| 1 | 22213521 | rs3820282 | AF086347 | HIT000067226 | 91,+ | tgG/tgA | W9* | PTC | AA->TER | No |
| 1 | 22733066 | rs14340 | CR595684 | HIT000274879 | 732,+ | Aag/Tag | K215* | PTC | AA->TER | No |
| 1 | 23867801 | rs16828732 | CR611350 | HIT000290545 | 1131,+ | Gag/Tag | E331* | PTC | AA->TER | No |
| 1 | 25303951 | rs11544666 | BC010862 | HIT000035081 | 108,+ | Cag/Tag | Q28* | PTC | AA->TER | Yes |
| 1 | 25911660 | rs1051170 | J04991 | HIT000191296 | 542,+ | Gaa/Taa | E147* | PTC | AA->TER | No |
| 1 | 26033901 | rs12070889 | U67191 | HIT000221010 | 1488,+ | Cga/Tga | R208* | PTC | AA->TER | Yes |
| 1 | 27834743 | rs8985 | CR609308 | HIT000288503 | 209,- | tTa/tAa | L60* | PTC | TER->AA | No |
| 1 | 28075213 | rs12038130 | AF083121 | HIT000066551 | 162,- | Cag/Tag | Q54* | PTC | AA->TER | No |
| 1 | 32950210 | rs6677618 | AK127879 | HIT000047752 | 1108,+ | Caa/Taa | Q3* | PTC | TER->AA | No |
| 1 | 33023641 | rs11554957 | BC001777 | HIT000030642 | 89,+ | Cga/Tga | R17* | PTC | AA->TER | Yes |
| 1 | 33441892 | rs11812052 | ENST00000330379 | HIT000126645 | 570,- | tgC/tgA | C190* | PTC | NA | No |
| 1 | 33667902 | rs4448486 | AY210418 | HIT000085277 | 9609,- | Gag/Tag | E3194* | PTC | AA->TER | Yes |
| 1 | 34997338 | rs4653109 | AK091069 | HIT000015936 | 1163,- | Taa/Caa | *47Q | Read-through | TER->AA | NA |
| 1 | 39127789 | rs2889682 | AL117451 | HIT000024613 | 1012,- | tTg/tAg | L8* | PTC | AA->TER | No |
| 1 | 39340932 | rs591994 | BC045188 | HIT000098021 | 2935,- | taA/taC | *82Y | Read-through | AA->TER | NA |
| 1 | 39522794 | rs687425 | AF317696 | HIT000077229 | 10627,+ | Gag/Tag | E3503* | PTC | AA->TER | Yes |
| 1 | 39522911 | rs687895 | AF317696 | HIT000077229 | 10744,+ | Gaa/Taa | E3542* | PTC | AA->TER | Yes |
| 1 | 39904481 | rs17852727 | AY013700 | HIT000083184 | 1633,- | Cag/Tag | Q514* | PTC | NA | No |
| Chr | Position | SNP ID | Accession no. | H-Inv ID | cDNA Pos. | Codon change | AA change | Effect | Inferred direction | NMD |
| 1 | 40442243 | rs12077871 | M95610 | HIT000196900 | 935,- | Cag/Tag | Q312* | PTC | NA | Yes |
| 1 | 40608488 | rs4562610 | CR605357 | HIT000284552 | 669,+ | taT/taG | Y34* | PTC | AA->TER | No |
| 1 | 44915336 | rs11211032 | ENST00000308498 | HIT000118004 | 145,- | Gaa/Taa | E49* | PTC | AA->TER | No |
| 1 | 44940366 | rs11549457 | AJ293866 | HIT000246647 | 2103,- | Cag/Tag | Q622* | PTC | AA->TER | No |
| 1 | 45148755 | rs11541961 | AF104421 | HIT000069424 | 574,+ | Cag/Tag | Q186* | PTC | AA->TER | Yes |
| 1 | 48420248 | rs850763 | BX648549 | HIT000057196 | 1858,- | Gag/Tag | E618* | PTC | AA->TER | Yes |
| 1 | 52201342 | rs2794990 | AK094262 | HIT000019117 | 993,- | tCa/tAa | S84* | PTC | AA->TER | No |
| 1 | 54205441 | rs6699257 | AK057652 | HIT000014261 | 1065,+ | tgA/tgG | *81W | Read-through | AA->TER | NA |
| 1 | 55250091 | rs1927330 | AB028980 | HIT000000774 | 2681,- | Gaa/Taa | E894* | PTC | AA->TER | Yes |
| 1 | 63368165 | rs12128444 | BC040309 | HIT000096112 | 787,- | Tga/Cga | *93R | Read-through | AA->TER | NA |
| 1 | 67882031 | rs11209142 | AK092014 | HIT000016881 | 2785,+ | tTa/tAa | L20* | PTC | AA->TER | No |
| 1 | 67882276 | rs9549 | AK092014 | HIT000016881 | 3029,- | tgC/tgA | C101* | PTC | AA->TER | No |
| 1 | 74916440 | rs1055553 | AK098237 | HIT000023067 | 390,+ | Gaa/Taa | E101* | PTC | AA->TER | Yes |
| 1 | 78670468 | rs1123153 | BC035694 | HIT000051476 | 214,- | Aaa/Taa | K7* | PTC | AA->TER | Yes |
| 1 | 86538195 | rs17854439 | AB033055 | HIT000000945 | 1471,- | Gaa/Taa | E397* | PTC | AA->TER | Yes |
| 1 | 86813396 | rs2292830 | AF043976 | HIT000063923 | 270,- | taC/taG | Y84* | PTC | TER->AA | Yes |
| 1 | 89188610 | rs12087251 | CR936755 | HIT000307028 | 1566,- | Cag/Tag | Q454* | PTC | AA->TER | Yes |
| 1 | 89293671 | rs4656097 | AK002150 | HIT000004624 | 1309,+ | taT/taA | Y61* | PTC | AA->TER | No |
| 1 | 90892311 | rs1335726 | AY338397 | HIT000251938 | 691,- | Gag/Tag | E217* | PTC | AA->TER | Yes |

| Chr | Position | SNP ID | Accession no. | H-Inv ID | cDNA Pos. | Codon change | AA change | Effect | Inferred direction | NMD |
| --- | --- | --- | --- | --- | --- | --- | --- | --- | --- | --- |
| 1 | 93012432 | rs11540831 | CR612523 | HIT000291718 | 327,+ | Aag/Tag | K89* | PTC | AA->TER | Yes |
| 1 | 94912040 | rs2640046 | AK025017 | HIT000008291 | 1857,+ | tgA/tgT | *88C | Read-through | TER->AA | NA |
| 1 | 103165218 | rs12735019 | J04177 | HIT000191251 | 2655,- | Gga/Tga | G832* | PTC | AA->TER | Yes |
| 1 | 109762876 | rs7349096 | BC040879 | HIT000052457 | 1051,- | tCa/tGa | S234* | PTC | AA->TER | No |
| 1 | 111109058 | rs12748133 | ENST00000361218 | HIT000314487 | 96,+ | tgG/tgA | W32* | PTC | AA->TER | No |
| 1 | 114050073 | rs12071676 | AK001115 | HIT000003589 | 1711,+ | Cga/Tga | R43* | PTC | AA->TER | No |
| 1 | 114948099 | rs17602729 | M60092 | HIT000195920 | 118,- | Caa/Taa | Q12* | PTC | AA->TER | Yes |
| 1 | 116843442 | rs4081622 | AF031174 | HIT000063055 | 2521,- | Gag/Tag | E806* | PTC | NA | Yes |
| 1 | 120096234 | rs6656217 | ENST00000357046 | HIT000310952 | 481,- | Cga/Tga | R104* | PTC | AA->TER | Yes |
| 1 | 120745363 | rs12133377 | BC017972 | HIT000089497 | 348,- | taC/taG | Y56* | PTC | NA | No |
| 1 | 120745392 | rs6677164 | BC017972 | HIT000089497 | 377,- | tTg/tAg | L66* | PTC | NA | No |
| 1 | 142106220 | rs11261229 | AK130052 | HIT000049323 | 373,+ | Cag/Tag | Q48* | PTC | AA->TER | Yes |
| 1 | 142592991 | rs2794062 | BC001364 | HIT000030350 | 234,- | Caa/Taa | Q39* | PTC | NA | Yes |
| 1 | 142601464 | rs2794041 | BC001364 | HIT000030350 | 513,- | Cga/Tga | R132* | PTC | AA->TER | Yes |
| 1 | 142601545 | rs3871984 | BC001364 | HIT000030350 | 594,- | Cga/Tga | R159* | PTC | AA->TER | No |
| 1 | 142777153 | rs765921 | AK022008 | HIT000005282 | 1227,- | tCa/tAa | S20* | PTC | NA | No |
| 1 | 142777322 | rs3872110 | AK000887 | HIT000003362 | 238,+ | Caa/Taa | Q6* | PTC | NA | No |
| 1 | 143562916 | rs2896830 | BC033999 | HIT000093327 | 212,- | taT/taA | Y68* | PTC | AA->TER | Yes |
| 1 | 144867764 | rs2999707 | AK096567 | HIT000021422 | 222,- | tGg/tAg | W10* | PTC | AA->TER | Yes |

| Chr | Position | SNP ID | Accession no. | H-Inv ID | cDNA Pos. | Codon change | AA change | Effect | Inferred direction | NMD |
| --- | --- | --- | --- | --- | --- | --- | --- | --- | --- | --- |
| 1 | 145038311 | rs28536572 | AK026418 | HIT000009692 | 170,- | Gag/Tag | E55* | PTC | NA | No |
| 1 | 145712477 | rs3188210 | ENST00000271699 | HIT000110763 | 100,+ | Cag/Tag | Q34* | PTC | NA | No |
| 1 | 146011975 | rs2787838 | BC062745 | HIT000260636 | 906,+ | Cag/Tag | Q21* | PTC | TER->AA | No |
| 1 | 146596823 | rs2457472 | ENST00000356530 | HIT000310511 | 129,- | taC/taG | Y43* | PTC | NA | No |
| 1 | 147299016 | rs3209760 | CR603901 | HIT000283096 | 1775,+ | tgA/tgG | *541W | Read-through | TER->AA | NA |
| 1 | 147411240 | rs17847055 | AK001981 | HIT000004455 | 427,+ | Cag/Tag | Q101* | PTC | AA->TER | No |
| 1 | 148188674 | rs6694569 | CR619323 | HIT000298518 | 373,+ | tCa/tGa | S2* | PTC | AA->TER | No |
| 1 | 148191412 | rs12023222 | BX537838 | HIT000054622 | 3488,- | Gga/Tga | G1058* | PTC | AA->TER | No |
| 1 | 148617286 | rs17582155 | BC031554 | HIT000041307 | 54,- | Cga/Tga | R10* | PTC | AA->TER | Yes |
| 1 | 149297318 | rs2282298 | NM_178438 | HIT000318088 | 411,- | Cga/Tga | R79* | PTC | NA | No |
| 1 | 149670269 | rs11549986 | BC014593 | HIT000036795 | 443,+ | Caa/Taa | Q100* | PTC | AA->TER | No |
| 1 | 150718539 | rs1122186 | AB007945 | HIT000000193 | 3732,+ | tCg/tAg | S1055* | PTC | AA->TER | Yes |
| 1 | 153293397 | rs10159180 | BC036686 | HIT000094562 | 679,+ | Cga/Tga | R72* | PTC | AA->TER | No |
| 1 | 153483852 | rs11549891 | BC001109 | HIT000030141 | 259,+ | Cag/Tag | Q46* | PTC | AA->TER | Yes |
| 1 | 154915183 | rs927663 | BC029599 | HIT000091865 | 558,- | tCa/tAa | S52* | PTC | TER->AA | No |
| 1 | 154960577 | rs859000 | ENST00000335586 | HIT000129694 | 17,- | tCg/tAg | S6* | PTC | AA->TER | Yes |
| 1 | 155362565 | rs863362 | NM_001004477 | HIT000315929 | 198,- | tgG/tgA | W66* | PTC | AA->TER | No |
| 1 | 155989313 | rs3027022 | U01839 | HIT000217426 | 1192,+ | taG/taC | *339Y | Read-through | TER->AA | NA |
| 1 | 156223413 | rs12409540 | BC069150 | HIT000263322 | 792,+ | tgT/tgA | C264* | PTC | AA->TER | No |

| Chr | Position | SNP ID | Accession no. | H-Inv ID | cDNA Pos. | Codon change | AA change | Effect | Inferred direction | NMD |
| --- | --- | --- | --- | --- | --- | --- | --- | --- | --- | --- |
| 1 | 156598443 | rs4301626 | AK131201 | HIT000249489 | 1326,+ | Tga/Cga | *414R | Read-through | TER->AA | NA |
| 1 | 156612881 | rs10430458 | AF146761 | HIT000071366 | 281,+ | Cga/Tga | R65* | PTC | NA | Yes |
| 1 | 157045579 | rs947496 | AK026213 | HIT000009487 | 980,- | Aaa/Taa | K62* | PTC | AA->TER | Yes |
| 1 | 157582807 | rs12049314 | AF244129 | HIT000074871 | 346,+ | Cga/Tga | R106* | PTC | AA->TER | Yes |
| 1 | 158289259 | rs9427397 | M31932 | HIT000195446 | 194,+ | Cag/Tag | Q63* | PTC | AA->TER | Yes |
| 1 | 158316553 | rs3856218 | AK092435 | HIT000017302 | 349,- | tTg/tAg | L66* | PTC | AA->TER | No |
| 1 | 158325922 | rs1042207 | CR604666 | HIT000283861 | 805,+ | Cga/Tga | R234* | PTC | NA | No |
| 1 | 158372884 | rs10917661 | AF543826 | HIT000243781 | 249,+ | Cag/Tag | Q57* | PTC | AA->TER | Yes |
| 1 | 159481692 | rs15941 | X74764 | HIT000323468 | 2919,+ | Tga/Cga | *856R | Read-through | TER->AA | NA |
| 1 | 163321704 | rs488982 | BC014341 | HIT000036650 | 326,- | tCa/tAa | S60* | PTC | AA->TER | Yes |
| 1 | 164756859 | rs1050297 | AY533031 | HIT000255201 | 316,+ | Gaa/Taa | E106* | PTC | AA->TER | No |
| 1 | 164947233 | rs2205697 | BC007072 | HIT000033030 | 1007,+ | Caa/Taa | Q36* | PTC | AA->TER | No |
| 1 | 165429794 | rs998689 | CR596260 | HIT000275455 | 293,+ | Gag/Tag | E93* | PTC | AA->TER | Yes |
| 1 | 167904779 | rs2020866 | BC005894 | HIT000032504 | 809,+ | Cga/Tga | R249* | PTC | AA->TER | Yes |
| 1 | 167909748 | rs6661174 | BC005894 | HIT000032504 | 1478,+ | Tag/Cag | *472Q | Read-through | AA->TER | NA |
| 1 | 176257971 | rs12568913 | AJ279254 | HIT000246492 | 655,- | Cga/Tga | R196* | PTC | AA->TER | Yes |
| 1 | 181163211 | rs1547606 | AF361250 | HIT000078377 | 380,- | Tga/Cga | *34R | Read-through | AA->TER | NA |
| 1 | 182818326 | rs12135336 | AF156100 | HIT000071961 | 11994,+ | tCg/tAg | S3922* | PTC | AA->TER | Yes |
| 1 | 199196432 | rs3817223 | AF324888 | HIT000077464 | 2557,+ | Cga/Tga | R805* | PTC | AA->TER | Yes |

| Chr | Position | SNP ID | Accession no. | H-Inv ID | cDNA Pos. | Codon change | AA change | Effect | Inferred direction | NMD |
| --- | --- | --- | --- | --- | --- | --- | --- | --- | --- | --- |
| 1 | 200738204 | rs11803208 | AK097662 | HIT000022516 | 474,- | Cag/Tag | Q115* | PTC | AA->TER | Yes |
| 1 | 206966302 | rs2228897 | AK001586 | HIT000004060 | 764,+ | Cag/Tag | Q190* | PTC | AA->TER | Yes |
| 1 | 207019668 | rs3765836 | AK128135 | HIT000048008 | 740,- | Cag/Tag | Q136* | PTC | TER->AA | No |
| 1 | 207769117 | rs12083429 | CR608515 | HIT000287710 | 292,+ | Cag/Tag | Q48* | PTC | AA->TER | No |
| 1 | 208613275 | rs9701867 | ENST00000294823 | HIT000113972 | 469,- | Gaa/Taa | E157* | PTC | AA->TER | No |
| 1 | 209559008 | rs17857328 | AL079275 | HIT000250114 | 1527,- | tCa/tAa | S457* | PTC | AA->TER | Yes |
| 1 | 216594243 | rs10987 | X54326 | HIT000321887 | 251,+ | Cag/Tag | Q65* | PTC | NA | Yes |
| 1 | 222326321 | rs4986931 | BC003567 | HIT000031605 | 315,+ | tGg/tAg | W97* | PTC | AA->TER | Yes |
| 1 | 224776638 | rs3795786 | AJ002535 | HIT000243960 | 8538,+ | Aga/Tga | R2823* | PTC | AA->TER | Yes |
| 1 | 224833382 | rs12740026 | AJ002535 | HIT000243960 | 17249,+ | tgC/tgA | C5726* | PTC | AA->TER | Yes |
| 1 | 226723685 | rs11800118 | CR608732 | HIT000287927 | 893,- | tGg/tAg | W2* | PTC | AA->TER | No |
| 1 | 227153175 | rs5039 | K02215 | HIT000191381 | 196,+ | Cag/Tag | Q53* | PTC | AA->TER | Yes |
| 1 | 227971783 | rs11559026 | X95073 | HIT000324888 | 224,+ | Caa/Taa | Q22* | PTC | AA->TER | Yes |
| 1 | 228405582 | rs12754734 | AK128283 | HIT000048156 | 2069,+ | Cga/Tga | R116* | PTC | AA->TER | No |
| 1 | 233032341 | rs2273865 | AF074001 | HIT000065977 | 1015,+ | tTg/tAg | L212* | PTC | AA->TER | Yes |
| 1 | 237998003 | rs11545658 | BC003108 | HIT000031436 | 704,+ | Cag/Tag | Q227* | PTC | AA->TER | Yes |
| 1 | 238081494 | rs652619 | AF056032 | HIT000064679 | 1505,+ | Tga/Cga | *487R | Read-through | TER->AA | NA |
| 1 | 239546311 | rs4001216 | AL137733 | HIT000026021 | 3180,+ | tgG/tgA | W102* | PTC | NA | No |
| 1 | 239967420 | rs12563956 | AK024613 | HIT000007887 | 506,+ | taG/taT | *57Y | Read-through | TER->AA | NA |

| Chr | Position | SNP ID | Accession no. | H-Inv ID | cDNA Pos. | Codon change | AA change | Effect | Inferred direction | NMD |
| --- | --- | --- | --- | --- | --- | --- | --- | --- | --- | --- |
| 1 | 243745550 | rs1778540 | NM_173858 | HIT000215130 | 136,+ | Caa/Taa | Q46* | PTC | AA->TER | No |
| 1 | 244264179 | rs6699571 | AY358215 | HIT000252259 | 1687,- | tGg/tAg | W31* | PTC | AA->TER | No |
| 1 | 244264949 | rs6587482 | ENST00000319427 | HIT000122059 | 637,+ | Tag/Cag | *213Q | Read-through | AA->TER | NA |
| 1 | 244439067 | rs10888281 | NM_001001963 | HIT000315572 | 867,+ | taT/taA | Y289* | PTC | AA->TER | No |
| 2 | 6063114 | rs11691189 | AK091363 | HIT000016230 | 1427,+ | Caa/Taa | Q154* | PTC | AA->TER | No |
| 2 | 6074090 | rs2709437 | AK123041 | HIT000042914 | 1158,+ | Cag/Tag | Q143* | PTC | TER->AA | No |
| 2 | 9678164 | rs4722 | BC056867 | HIT000259592 | 787,+ | Cag/Tag | Q219* | PTC | NA | No |
| 2 | 10220079 | rs15516 | X59618 | HIT000322329 | 1332,+ | Gag/Tag | E440* | PTC | AA->TER | No |
| 2 | 10534922 | rs11538369 | CR614398 | HIT000293593 | 829,+ | Caa/Taa | Q116* | PTC | AA->TER | Yes |
| 2 | 11442702 | rs12993982 | ENST00000357907 | HIT000311693 | 382,+ | Aag/Tag | K118* | PTC | AA->TER | No |
| 2 | 12114787 | rs4668745 | ENST00000323501 | HIT000123517 | 502,+ | Cga/Tga | R168* | PTC | AA->TER | Yes |
| 2 | 12114844 | rs4669797 | ENST00000323501 | HIT000123517 | 559,+ | Cga/Tga | R187* | PTC | AA->TER | Yes |
| 2 | 25171500 | rs6737356 | CR933670 | HIT000306851 | 1945,+ | Cag/Tag | Q70* | PTC | AA->TER | No |
| 2 | 27575369 | rs11539699 | AK122664 | HIT000042537 | 2072,+ | Cag/Tag | Q414* | PTC | AA->TER | Yes |
| 2 | 29518318 | rs4363989 | U62540 | HIT000220727 | 2124,- | Gaa/Taa | E405* | PTC | AA->TER | Yes |
| 2 | 30293959 | rs1060243 | CR615384 | HIT000294579 | 1042,- | Tga/Gga | *105G | Read-through | AA->TER | NA |
| 2 | 31717605 | rs9332960 | M74047 | HIT000196297 | 43,+ | Cag/Tag | Q6* | PTC | NA | Yes |
| 2 | 38888853 | rs11547149 | BC022328 | HIT000039475 | 266,+ | Gaa/Taa | E55* | PTC | AA->TER | Yes |
| 2 | 40393275 | rs2514 | BC043380 | HIT000097640 | 250,+ | taC/taG | Y76* | PTC | NA | No |

| Chr | Position | SNP ID | Accession no. | H-Inv ID | cDNA Pos. | Codon change | AA change | Effect | Inferred direction | NMD |
| --- | --- | --- | --- | --- | --- | --- | --- | --- | --- | --- |
| 2 | 42490105 | rs11547561 | AK092911 | HIT000017778 | 1080,+ | Caa/Taa | Q84* | PTC | AA->TER | No |
| 2 | 42750049 | rs1534760 | AK126724 | HIT000046597 | 616,- | Tag/Cag | *132Q | Read-through | TER->AA | NA |
| 2 | 45147648 | rs11540435 | AF332197 | HIT000077699 | 546,+ | Cag/Tag | Q85* | PTC | AA->TER | Yes |
| 2 | 46513506 | rs12464760 | AK023572 | HIT000006846 | 1281,+ | tCa/tAa | S69* | PTC | AA->TER | No |
| 2 | 47095643 | rs10166433 | AK056464 | HIT000013078 | 694,+ | tgT/tgA | C87* | PTC | AA->TER | Yes |
| 2 | 61282352 | rs1055499 | AK023856 | HIT000007130 | 558,+ | tTa/tAa | L93* | PTC | NA | No |
| 2 | 64751635 | rs6546090 | BC041356 | HIT000096636 | 148,+ | Gag/Tag | E29* | PTC | AA->TER | Yes |
| 2 | 69979767 | rs3771532 | BC007420 | HIT000033208 | 731,- | tTg/tAg | L175* | PTC | AA->TER | Yes |
| 2 | 73839586 | rs4852974 | AF185571 | HIT000072892 | 537,- | Cag/Tag | Q168* | PTC | AA->TER | No |
| 2 | 74281106 | rs17854186 | BC042036 | HIT000052637 | 240,- | tAa/tCa | *69S | Read-through | TER->AA | NA |
| 2 | 74554546 | rs1055238 | ENST00000290390 | HIT000113274 | 128,- | tCa/tGa | S43* | PTC | AA->TER | Yes |
| 2 | 74600059 | rs1051283 | X87237 | HIT000324275 | 2641,+ | Tga/Aga | *837R | Read-through | TER->AA | NA |
| 2 | 85453626 | rs6757590 | AK098125 | HIT000022963 | 1666,+ | tgG/tgA | W10* | PTC | AA->TER | No |
| 2 | 86349387 | rs1052055 | BC020651 | HIT000038872 | 539,+ | Cga/Tga | R169* | PTC | AA->TER | No |
| 2 | 86629666 | rs17853822 | BX647752 | HIT000056399 | 3734,+ | tCa/tAa | S1193* | PTC | AA->TER | Yes |
| 2 | 86742806 | rs11695337 | D76444 | HIT000101730 | 2798,- | Gag/Tag | E626* | PTC | AA->TER | No |
| 2 | 88765766 | rs13034488 | AF110146 | HIT000069623 | 358,- | Gag/Tag | E96* | PTC | AA->TER | Yes |
| 2 | 88949523 | rs7572174 | CR623415 | HIT000302610 | 1106,- | tCa/tGa | S34* | PTC | AA->TER | No |
| 2 | 91056707 | rs638276 | ENST00000359143 | HIT000312730 | 663,+ | tgG/tgA | W221* | PTC | NA | No |

| Chr | Position | SNP ID | Accession no. | H-Inv ID | cDNA Pos. | Codon change | AA change | Effect | Inferred direction | NMD |
| --- | --- | --- | --- | --- | --- | --- | --- | --- | --- | --- |
| 2 | 91199717 | rs2647770 | ENST00000343942 | HIT000136572 | 304,- | Cag/Tag | Q102* | PTC | NA | Yes |
| 2 | 91199727 | rs2599165 | ENST00000343942 | HIT000136572 | 314,+ | tGg/tAg | W105* | PTC | NA | Yes |
| 2 | 91212932 | rs2557974 | ENST00000343942 | HIT000136572 | 582,- | tgG/tgA | W194* | PTC | TER->AA | Yes |
| 2 | 91231276 | rs4244681 | ENST00000343942 | HIT000136572 | 1797,- | taT/taG | Y599* | PTC | NA | No |
| 2 | 95941462 | rs4063025 | ENST00000357042 | HIT000310948 | 251,- | Caa/Taa | Q84* | PTC | NA | Yes |
| 2 | 96682282 | rs28362544 | CR602875 | HIT000282070 | 1471,- | tGg/tAg | W38* | PTC | AA->TER | No |
| 2 | 96948662 | rs12471298 | AY358842 | HIT000252886 | 2210,- | Gag/Tag | E693* | PTC | AA->TER | No |
| 2 | 98684921 | rs11550070 | AL080115 | HIT000024236 | 337,+ | Cag/Tag | Q61* | PTC | AA->TER | Yes |
| 2 | 101080985 | rs11551487 | BC070373 | HIT000264328 | 303,+ | Gag/Tag | E94* | PTC | AA->TER | Yes |
| 2 | 101095755 | rs13398805 | AK093543 | HIT000018410 | 384,- | Caa/Taa | Q9* | PTC | AA->TER | Yes |
| 2 | 108570429 | rs2581003 | AB002334 | HIT000000042 | 4562,+ | Cag/Tag | Q1437* | PTC | NA | Yes |
| 2 | 108760906 | rs700878 | AK025661 | HIT000008935 | 1342,+ | tgG/tgA | W57* | PTC | AA->TER | No |
| 2 | 111709595 | rs17041941 | AK023908 | HIT000007182 | 1060,+ | tGg/tAg | W39* | PTC | AA->TER | No |
| 2 | 114085812 | rs3982542 | BC000596 | HIT000029763 | 416,- | tgA/tgG | *82W | Read-through | NA | NA |
| 2 | 118333628 | rs2570189 | ENST00000245790 | HIT000105565 | 395,+ | tGg/tAg | W132* | PTC | NA | Yes |
| 2 | 130502186 | rs10211003 | AK125921 | HIT000045794 | 2913,- | taC/taG | Y105* | PTC | NA | Yes |
| 2 | 131423217 | rs7423073 | AK091000 | HIT000015867 | 1890,- | tgG/tgA | W64* | PTC | AA->TER | No |
| 2 | 132944625 | rs6756492 | BC045732 | HIT000098162 | 630,+ | tAg/tGg | *210W | Read-through | AA->TER | NA |
| 2 | 138879719 | rs13033503 | CR608093 | HIT000287288 | 460,- | tGg/tAg | W107* | PTC | AA->TER | No |

| Chr | Position | SNP ID | Accession no. | H-Inv ID | cDNA Pos. | Codon change | AA change | Effect | Inferred direction | NMD |
| --- | --- | --- | --- | --- | --- | --- | --- | --- | --- | --- |
| 2 | 156703282 | rs6748811 | BC032407 | HIT000092641 | 1019,- | Taa/Gaa | *43E | Read-through | AA->TER | NA |
| 2 | 158062329 | rs16841815 | ENST00000355813 | HIT000309900 | 106,- | Cga/Tga | R36* | PTC | NA | No |
| 2 | 158452509 | rs17419471 | L02911 | HIT000191501 | 1009,+ | tgG/tgA | W223* | PTC | NA | Yes |
| 2 | 159912078 | rs6755758 | BX648327 | HIT000056974 | 4168,+ | tCg/tAg | S1389* | PTC | AA->TER | No |
| 2 | 162656269 | rs12991239 | BC051190 | HIT000099284 | 3254,+ | Gaa/Taa | E1025* | PTC | AA->TER | Yes |
| 2 | 167126930 | rs2091544 | M91556 | HIT000196807 | 1614,- | tTa/tGa | L492* | PTC | AA->TER | Yes |
| 2 | 170370864 | rs13009265 | AK001460 | HIT000003934 | 192,- | Cga/Tga | R4* | PTC | AA->TER | No |
| 2 | 170385331 | rs1374466 | AK021962 | HIT000005236 | 1473,- | tgC/tgA | C43* | PTC | TER->AA | No |
| 2 | 172006278 | rs10205459 | AK124092 | HIT000043965 | 1449,- | Cga/Tga | R379* | PTC | AA->TER | No |
| 2 | 174645546 | rs1804452 | AY441957 | HIT000254712 | 1732,+ | Caa/Taa | Q401* | PTC | AA->TER | Yes |
| 2 | 182817713 | rs16822667 | AK056136 | HIT000012750 | 544,+ | Caa/Taa | Q16* | PTC | AA->TER | No |
| 2 | 182843986 | rs17366198 | AK130643 | HIT000049914 | 1003,- | taC/taA | Y10* | PTC | AA->TER | No |
| 2 | 198082670 | rs12992563 | AF054284 | HIT000064546 | 3778,- | Aaa/Taa | K1260* | PTC | AA->TER | No |
| 2 | 201170779 | rs11899656 | AK021816 | HIT000005090 | 492,+ | tTa/tGa | L53* | PTC | AA->TER | No |
| 2 | 201792891 | rs4673041 | AK130593 | HIT000049864 | 792,+ | tTa/tGa | L29* | PTC | AA->TER | No |
| 2 | 201970865 | rs13422553 | AK055262 | HIT000011876 | 593,- | tgG/tgA | W78* | PTC | AA->TER | Yes |
| 2 | 202805759 | rs2882485 | BC040292 | HIT000096099 | 1535,+ | Cga/Tga | R39* | PTC | TER->AA | Yes |
| 2 | 202897156 | rs11545248 | CR623467 | HIT000302662 | 393,- | Gaa/Taa | E9* | PTC | AA->TER | No |
| 2 | 207305208 | rs6732127 | AK091800 | HIT000016667 | 2976,- | tgG/tgA | W34* | PTC | AA->TER | No |

| Chr | Position | SNP ID | Accession no. | H-Inv ID | cDNA Pos. | Codon change | AA change | Effect | Inferred direction | NMD |
| --- | --- | --- | --- | --- | --- | --- | --- | --- | --- | --- |
| 2 | 208927309 | rs3778 | CR608733 | HIT000287928 | 1491,+ | taA/taC | *415Y | Read-through | TER->AA | NA |
| 2 | 210722929 | rs13017647 | AK093026 | HIT000017893 | 1311,+ | taT/taA | Y3* | PTC | AA->TER | Yes |
| 2 | 213398686 | rs3820708 | AK026761 | HIT000010035 | 351,+ | tAa/tCa | *101S | Read-through | TER->AA | NA |
| 2 | 213968137 | rs13000409 | AK124960 | HIT000044833 | 372,- | tCa/tGa | S82* | PTC | TER->AA | No |
| 2 | 217105502 | rs4338942 | AF432223 | HIT000079609 | 709,+ | tTa/tGa | L190* | PTC | AA->TER | Yes |
| 2 | 217105551 | rs5014981 | AF432223 | HIT000079609 | 758,+ | taC/taG | Y206* | PTC | AA->TER | Yes |
| 2 | 220230224 | rs6722853 | AB095813 | HIT000242118 | 1950,- | Gag/Tag | E572* | PTC | AA->TER | No |
| 2 | 220376401 | rs11678220 | AK056734 | HIT000013348 | 1170,- | Gag/Tag | E48* | PTC | AA->TER | Yes |
| 2 | 234166698 | rs9941591 | BC047714 | HIT000098760 | 559,+ | tGg/tAg | W79* | PTC | AA->TER | No |
| 2 | 236683480 | rs13403732 | AK127068 | HIT000046941 | 2232,- | tgT/tgA | C37* | PTC | AA->TER | No |
| 2 | 237780538 | rs11553715 | BC036499 | HIT000094446 | 890,+ | Gga/Tga | G78* | PTC | AA->TER | Yes |
| 2 | 241118490 | rs11558615 | X54232 | HIT000321883 | 441,+ | Gag/Tag | E74* | PTC | AA->TER | Yes |
| 2 | 241813006 | rs11678888 | CR609492 | HIT000288687 | 184,+ | Gaa/Taa | E60* | PTC | AA->TER | Yes |
| 2 | 242012269 | rs5022214 | CR608761 | HIT000287956 | 1142,+ | Tga/Gga | *102G | Read-through | TER->AA | NA |
| 2 | 242012558 | rs12620226 | CR608761 | HIT000287956 | 853,- | tgG/tgA | W5* | PTC | AA->TER | No |
| 3 | 8589542 | rs17851386 | AF086709 | HIT000067452 | 652,+ | tTa/tGa | L29* | PTC | NA | Yes |
| 3 | 13953212 | rs9647360 | AK056482 | HIT000013096 | 3141,+ | tgA/tgT | *214C | Read-through | TER->AA | NA |
| 3 | 17213576 | rs17043178 | AK022411 | HIT000005685 | 1310,+ | tGg/tAg | W33* | PTC | AA->TER | No |
| 3 | 23917485 | rs11537715 | AK027749 | HIT000011021 | 533,+ | Gga/Tga | G52* | PTC | AA->TER | Yes |

| Chr | Position | SNP ID | Accession no. | H-Inv ID | cDNA Pos. | Codon change | AA change | Effect | Inferred direction | NMD |
| --- | --- | --- | --- | --- | --- | --- | --- | --- | --- | --- |
| 3 | 25810987 | rs9851096 | AK000611 | HIT000003086 | 1477,+ | Tag/Cag | *460Q | Read-through | TER->AA | NA |
| 3 | 27848092 | rs11924141 | BC047650 | HIT000098732 | 2063,- | Cag/Tag | Q9* | PTC | AA->TER | No |
| 3 | 30688682 | rs17854016 | M85079 | HIT000196604 | 1338,+ | Gag/Tag | E335* | PTC | AA->TER | Yes |
| 3 | 37033015 | rs4647274 | CR607691 | HIT000286886 | 501,- | taG/taT | *156Y | Read-through | AA->TER | NA |
| 3 | 38023037 | rs2364781 | BC004300 | HIT000031851 | 2415,+ | Gag/Tag | E753* | PTC | AA->TER | Yes |
| 3 | 38238123 | rs2936810 | AB017642 | HIT000058528 | 883,- | Aaa/Taa | K181* | PTC | AA->TER | Yes |
| 3 | 44675385 | rs3732518 | X07289 | HIT000321250 | 715,- | Caa/Taa | Q176* | PTC | AA->TER | Yes |
| 3 | 47444038 | rs2046376 | D83782 | HIT000042334 | 779,- | tgG/tgA | W178* | PTC | AA->TER | Yes |
| 3 | 49370761 | rs1800668 | Y00433 | HIT000325288 | 273,+ | Cag/Tag | Q10* | PTC | NA | Yes |
| 3 | 49824429 | rs13071187 | L13852 | HIT000192228 | 981,- | Cag/Tag | Q273* | PTC | AA->TER | Yes |
| 3 | 49910507 | rs9819888 | X70040 | HIT000323081 | 1889,- | Aag/Tag | K621* | PTC | AA->TER | Yes |
| 3 | 50126410 | rs17849691 | AF091263 | HIT000068321 | 1789,+ | tgG/tgA | W547* | PTC | AA->TER | Yes |
| 3 | 51401559 | rs11538558 | BC007282 | HIT000033117 | 550,+ | tAg/tGg | *183W | Read-through | TER->AA | NA |
| 3 | 51883266 | rs1505403 | AK097471 | HIT000022325 | 532,+ | Cag/Tag | Q118* | PTC | AA->TER | Yes |
| 3 | 53874316 | rs1043261 | BC000980 | HIT000030042 | 1459,+ | Cag/Tag | Q484* | PTC | AA->TER | No |
| 3 | 54905813 | rs17253119 | AK092143 | HIT000017010 | 310,- | Cga/Tga | R61* | PTC | TER->AA | Yes |
| 3 | 57977925 | rs839239 | AK098160 | HIT000022992 | 229,+ | Cag/Tag | Q12* | PTC | TER->AA | No |
| 3 | 68767991 | rs11557331 | CR457044 | HIT000267894 | 446,+ | tCg/tAg | S149* | PTC | NA | No |
| 3 | 70749319 | rs4974307 | AK125942 | HIT000045815 | 184,- | Cga/Tga | R37* | PTC | AA->TER | No |

| Chr | Position | SNP ID | Accession no. | H-Inv ID | cDNA Pos. | Codon change | AA change | Effect | Inferred direction | NMD |
| --- | --- | --- | --- | --- | --- | --- | --- | --- | --- | --- |
| 3 | 73194414 | rs2231925 | BC051316 | HIT000099308 | 915,+ | taT/taG | Y164* | PTC | AA->TER | No |
| 3 | 73644866 | rs2241513 | AK130896 | HIT000050167 | 482,+ | tCg/tAg | S22* | PTC | AA->TER | No |
| 3 | 74131732 | rs1405404 | BC040672 | HIT000096321 | 87,+ | taC/taA | Y20* | PTC | TER->AA | No |
| 3 | 74136342 | rs6766876 | BC040672 | HIT000096321 | 263,+ | tAg/tTg | *79L | Read-through | TER->AA | NA |
| 3 | 78749727 | rs1065217 | AF040990 | HIT000063633 | 4030,+ | Aga/Tga | R1344* | PTC | AA->TER | Yes |
| 3 | 95086403 | rs5017717 | Y00692 | HIT000325306 | 1474,+ | Cga/Tga | R451* | PTC | AA->TER | Yes |
| 3 | 96856998 | rs9844012 | AK092228 | HIT000017095 | 1553,- | Aag/Tag | K60* | PTC | AA->TER | No |
| 3 | 97552228 | rs13096522 | ENST00000330649 | HIT000126791 | 750,+ | taT/taA | Y250* | PTC | AA->TER | No |
| 3 | 97552338 | rs6769771 | ENST00000330649 | HIT000126791 | 860,+ | tCg/tAg | S287* | PTC | AA->TER | No |
| 3 | 101135806 | rs768527 | AF090939 | HIT000068271 | 1151,- | tGg/tAg | W30* | PTC | AA->TER | No |
| 3 | 101915259 | rs11557850 | Y07968 | HIT000325374 | 58,+ | Aaa/Taa | K14* | PTC | AA->TER | Yes |
| 3 | 113183407 | rs11551651 | AK002204 | HIT000004678 | 239,+ | Gga/Tga | G77* | PTC | AA->TER | Yes |
| 3 | 113263361 | rs340142 | AK131211 | HIT000249499 | 1253,+ | Cga/Tga | R179* | PTC | AA->TER | Yes |
| 3 | 115437877 | rs3732781 | X65233 | HIT000322738 | 1247,+ | taT/taG | Y323* | PTC | AA->TER | Yes |
| 3 | 123773702 | rs4678198 | AK056406 | HIT000013020 | 2264,- | Cga/Tga | R113* | PTC | AA->TER | No |
| 3 | 127359968 | rs13088615 | CR749807 | HIT000306737 | 588,- | Cga/Tga | R124* | PTC | AA->TER | Yes |
| 3 | 127411525 | rs10804575 | AK097508 | HIT000022362 | 2050,+ | tGg/tAg | W13* | PTC | AA->TER | No |
| 3 | 130481349 | rs11550360 | AF201934 | HIT000002221 | 177,+ | Cag/Tag | Q26* | PTC | AA->TER | Yes |
| 3 | 131107330 | rs6772099 | AK127796 | HIT000047669 | 374,+ | Cga/Tga | R38* | PTC | AA->TER | No |

| Chr | Position | SNP ID | Accession no. | H-Inv ID | cDNA Pos. | Codon change | AA change | Effect | Inferred direction | NMD |
| --- | --- | --- | --- | --- | --- | --- | --- | --- | --- | --- |
| 3 | 131141403 | rs12489576 | BC033956 | HIT000093288 | 431,- | tGg/tAg | W79* | PTC | AA->TER | No |
| 3 | 131642028 | rs2201717 | AK123718 | HIT000043591 | 453,+ | Caa/Taa | Q89* | PTC | AA->TER | Yes |
| 3 | 132564913 | rs3749394 | AK000532 | HIT000003007 | 461,- | Tga/Cga | *123R | Read-through | TER->AA | NA |
| 3 | 134562175 | rs1849505 | BC040919 | HIT000096454 | 1272,- | tgT/tgA | C37* | PTC | TER->AA | Yes |
| 3 | 134674013 | rs899457 | U48224 | HIT000220048 | 1150,- | Cag/Tag | Q384* | PTC | NA | Yes |
| 3 | 135764977 | rs9834981 | AK123837 | HIT000043710 | 1615,+ | tTa/tGa | L20* | PTC | AA->TER | No |
| 3 | 140881118 | rs3922941 | BX640693 | HIT000055242 | 1962,- | tCa/tAa | S4* | PTC | AA->TER | No |
| 3 | 144031268 | rs17850059 | AK075478 | HIT000082511 | 1133,- | Gaa/Taa | E277* | PTC | AA->TER | Yes |
| 3 | 147706818 | rs13077712 | AK095172 | HIT000020027 | 228,- | tTa/tAa | L51* | PTC | AA->TER | No |
| 3 | 150857987 | rs6783790 | AK130903 | HIT000050174 | 429,+ | tgG/tgA | W38* | PTC | AA->TER | Yes |
| 3 | 168518065 | rs7612489 | BC034229 | HIT000042075 | 1291,- | Aaa/Taa | K307* | PTC | AA->TER | Yes |
| 3 | 171023380 | rs13062420 | ENST00000340806 | HIT000133436 | 978,+ | tgT/tgA | C326* | PTC | AA->TER | Yes |
| 3 | 180598270 | rs9873206 | AK091332 | HIT000016199 | 398,- | Cga/Tga | R81* | PTC | AA->TER | No |
| 3 | 180824529 | rs17849958 | BC005271 | HIT000032338 | 589,+ | tAg/tGg | *190W | Read-through | TER->AA | NA |
| 3 | 181004637 | rs1442495 | AK124818 | HIT000044691 | 1272,+ | Cga/Tga | R13* | PTC | NA | No |
| 3 | 185143305 | rs1053351 | AF104942 | HIT000069457 | 3731,+ | taC/taA | Y1202* | PTC | AA->TER | Yes |
| 3 | 186155214 | rs7628886 | AF244570 | HIT000074879 | 1023,- | Tag/Aag | *44K | Read-through | AA->TER | NA |
| 3 | 188278342 | rs1801380 | CR619308 | HIT000298503 | 675,- | Cag/Tag | Q91* | PTC | TER->AA | No |
| 3 | 189930193 | rs13071197 | AK128426 | HIT000048299 | 3012,- | tgT/tgA | C81* | PTC | AA->TER | No |

| Chr | Position | SNP ID | Accession no. | H-Inv ID | cDNA Pos. | Codon change | AA change | Effect | Inferred direction | NMD |
| --- | --- | --- | --- | --- | --- | --- | --- | --- | --- | --- |
| 3 | 192475746 | rs16866426 | AK090630 | HIT000015497 | 1117,- | Cga/Tga | R111* | PTC | AA->TER | No |
| 3 | 195043512 | rs13319746 | AK091265 | HIT000016132 | 1915,+ | Cga/Tga | R70* | PTC | TER->AA | Yes |
| 3 | 195543609 | rs4974538 | BC031569 | HIT000092405 | 1564,- | tGg/tAg | W509* | PTC | AA->TER | No |
| 3 | 197166198 | rs4927806 | AK126102 | HIT000045975 | 1829,+ | tgT/tgA | C41* | PTC | NA | No |
| 3 | 197455625 | rs6795511 | AF086176 | HIT000067055 | 507,+ | tgG/tgA | W49* | PTC | AA->TER | No |
| 3 | 197577775 | rs17854451 | AK026451 | HIT000009725 | 956,- | Cag/Tag | Q319* | PTC | AA->TER | Yes |
| 3 | 198850254 | rs11918438 | AJ271448 | HIT000246174 | 1414,+ | Gaa/Taa | E333* | PTC | NA | Yes |
| 3 | 199102430 | rs9883425 | BC008625 | HIT000087113 | 200,+ | Tag/Cag | *36Q | Read-through | TER->AA | NA |
| 4 | 145675 | rs7692722 | AK096662 | HIT000021517 | 2050,+ | tgT/tgA | C346* | PTC | AA->TER | No |
| 4 | 238055 | rs9994989 | BC046475 | HIT000098370 | 859,+ | taC/taA | Y52* | PTC | AA->TER | No |
| 4 | 820431 | rs2127168 | BC032898 | HIT000051092 | 413,- | tGg/tAg | W96* | PTC | AA->TER | No |
| 4 | 3261179 | rs3025816 | L12392 | HIT000192172 | 7753,- | Cag/Tag | Q2480* | PTC | AA->TER | Yes |
| 4 | 3801551 | rs28379753 | BX648772 | HIT000057419 | 536,- | tgC/tgA | C43* | PTC | AA->TER | Yes |
| 4 | 4194780 | rs6446338 | BC042823 | HIT000097358 | 1145,+ | tCa/tAa | S98* | PTC | TER->AA | No |
| 4 | 4194822 | rs13146943 | BC042823 | HIT000097358 | 1187,+ | tGa/tCa | *112S | Read-through | TER->AA | NA |
| 4 | 4973152 | rs6446693 | AK056311 | HIT000012925 | 505,- | tAg/tGg | *119W | Read-through | AA->TER | NA |
| 4 | 6322921 | rs11947696 | AK125859 | HIT000045732 | 594,+ | Cga/Tga | R103* | PTC | AA->TER | No |
| 4 | 7001586 | rs3822268 | CR613368 | HIT000292563 | 1552,- | Taa/Caa | *155Q | Read-through | TER->AA | NA |
| 4 | 7550260 | rs6854053 | BC063708 | HIT000261161 | 1117,- | tGg/tAg | W75* | PTC | AA->TER | No |

| Chr | Position | SNP ID | Accession no. | H-Inv ID | cDNA Pos. | Codon change | AA change | Effect | Inferred direction | NMD |
| --- | --- | --- | --- | --- | --- | --- | --- | --- | --- | --- |
| 4 | 7680243 | rs13146469 | AK126053 | HIT000045926 | 3301,- | Cga/Tga | R73* | PTC | NA | No |
| 4 | 7893591 | rs10026941 | BC043614 | HIT000097797 | 1660,+ | Cga/Tga | R89* | PTC | TER->AA | No |
| 4 | 9081495 | rs11727979 | ENST00000344638 | HIT000137268 | 157,+ | Cga/Tga | R53* | PTC | NA | No |
| 4 | 13335712 | rs6811186 | AK091889 | HIT000016756 | 246,+ | taC/taG | Y74* | PTC | TER->AA | Yes |
| 4 | 17164802 | rs1049582 | CR595211 | HIT000274406 | 245,- | Caa/Taa | Q12* | PTC | TER->AA | No |
| 4 | 21542915 | rs4697244 | AK057907 | HIT000014514 | 304,- | tAg/tGg | *83W | Read-through | TER->AA | NA |
| 4 | 22496768 | rs3209013 | AF323990 | HIT000077452 | 1412,+ | Gaa/Taa | E455* | PTC | AA->TER | No |
| 4 | 22496773 | rs17845885 | AF323990 | HIT000077452 | 1417,+ | taT/taA | Y456* | PTC | AA->TER | No |
| 4 | 24484587 | rs2270224 | AF086111 | HIT000066990 | 394,- | Cag/Tag | Q41* | PTC | AA->TER | No |
| 4 | 25355478 | rs17672707 | AK075045 | HIT000082080 | 1161,- | Gaa/Taa | E89* | PTC | TER->AA | No |
| 4 | 26102560 | rs5007634 | L07872 | HIT000191868 | 790,+ | Cga/Tga | R238* | PTC | AA->TER | Yes |
| 4 | 26108629 | rs1064382 | L07872 | HIT000191868 | 1312,+ | Cga/Tga | R412* | PTC | AA->TER | No |
| 4 | 26108645 | rs1064383 | L07872 | HIT000191868 | 1328,+ | tGg/tAg | W417* | PTC | AA->TER | No |
| 4 | 37511434 | rs28671096 | AK056064 | HIT000012678 | 1445,- | Aaa/Taa | K11* | PTC | NA | No |
| 4 | 39111874 | rs17754 | L24783 | HIT000192600 | 240,+ | taG/taC | *73Y | Read-through | TER->AA | NA |
| 4 | 44560184 | rs6818617 | AF247786 | HIT000074966 | 440,- | Aag/Tag | K95* | PTC | AA->TER | Yes |
| 4 | 49022944 | rs4235165 | BC022881 | HIT000090703 | 1574,+ | tGg/tAg | W78* | PTC | NA | No |
| 4 | 49332232 | rs4540670 | AK090412 | HIT000015279 | 2061,- | Gaa/Taa | E266* | PTC | NA | Yes |
| 4 | 49335462 | rs4253934 | BC035915 | HIT000094168 | 306,+ | Caa/Taa | Q60* | PTC | AA->TER | No |

| Chr | Position | SNP ID | Accession no. | H-Inv ID | cDNA Pos. | Codon change | AA change | Effect | Inferred direction | NMD |
| --- | --- | --- | --- | --- | --- | --- | --- | --- | --- | --- |
| 4 | 49345187 | rs28394139 | AK090412 | HIT000015279 | 1600,- | tTa/tAa | L112* | PTC | NA | Yes |
| 4 | 49423932 | rs2841697 | AK056396 | HIT000013010 | 1689,+ | Cga/Tga | R61* | PTC | AA->TER | No |
| 4 | 49429131 | rs3969392 | AK023528 | HIT000006802 | 472,- | tgG/tgA | W22* | PTC | AA->TER | Yes |
| 4 | 57102538 | rs2660744 | D13757 | HIT000100514 | 1583,- | Caa/Taa | Q488* | PTC | AA->TER | No |
| 4 | 57638290 | rs1059742 | U22314 | HIT000218675 | 2387,+ | Gag/Tag | E780* | PTC | AA->TER | No |
| 4 | 57756425 | rs11941013 | AK001802 | HIT000004276 | 1261,+ | tgT/tgA | C19* | PTC | TER->AA | No |
| 4 | 69806136 | rs2271513 | AF242527 | HIT000074849 | 94,- | Cag/Tag | Q32* | PTC | AA->TER | No |
| 4 | 71079682 | rs17147990 | M26665 | HIT000195075 | 212,+ | taT/taA | Y47* | PTC | AA->TER | No |
| 4 | 77393742 | rs17850938 | BC012559 | HIT000035868 | 1518,- | Tga/Gga | *494G | Read-through | TER->AA | NA |
| 4 | 80139758 | rs17857084 | AL137661 | HIT000025949 | 1270,+ | tgA/tgG | *356W | Read-through | TER->AA | NA |
| 4 | 83709213 | rs11546516 | AF113125 | HIT000001906 | 273,+ | Gaa/Taa | E18* | PTC | AA->TER | Yes |
| 4 | 86955754 | rs7680209 | AK055572 | HIT000012186 | 1052,+ | tgG/tgA | W57* | PTC | AA->TER | No |
| 4 | 87980082 | rs2287149 | D21209 | HIT000101090 | 1207,- | Cga/Tga | R382* | PTC | AA->TER | Yes |
| 4 | 89391828 | rs3201997 | AF103796 | HIT000069389 | 1204,+ | Gag/Tag | E334* | PTC | AA->TER | Yes |
| 4 | 89720782 | rs4413373 | AF336798 | HIT000242883 | 3244,+ | Cag/Tag | Q1072* | PTC | TER->AA | No |
| 4 | 90044734 | rs2972039 | AK130910 | HIT000050181 | 2239,- | Gaa/Taa | E37* | PTC | AA->TER | No |
| 4 | 92117249 | rs11544907 | CR621963 | HIT000301158 | 182,+ | Caa/Taa | Q40* | PTC | AA->TER | No |
| 4 | 92117258 | rs11559255 | CR621963 | HIT000301158 | 173,+ | Cag/Tag | Q37* | PTC | AA->TER | No |
| 4 | 100625368 | rs283413 | M12272 | HIT000194152 | 312,+ | Gga/Tga | G78* | PTC | AA->TER | Yes |

| Chr | Position | SNP ID | Accession no. | H-Inv ID | cDNA Pos. | Codon change | AA change | Effect | Inferred direction | NMD |
| --- | --- | --- | --- | --- | --- | --- | --- | --- | --- | --- |
| 4 | 102443558 | rs931871 | BX538142 | HIT000054924 | 3912,+ | tCa/tGa | S41* | PTC | AA->TER | No |
| 4 | 104341578 | rs7675837 | AK096208 | HIT000021063 | 735,- | Cag/Tag | Q43* | PTC | AA->TER | No |
| 4 | 104439966 | rs2720466 | Z15005 | HIT000326516 | 2102,- | tTa/tAa | L671* | PTC | AA->TER | Yes |
| 4 | 110790812 | rs17040495 | AJ131245 | HIT000244634 | 2027,+ | tgG/tgA | W624* | PTC | AA->TER | Yes |
| 4 | 113547116 | rs11723375 | BX647702 | HIT000056349 | 1162,+ | Gaa/Taa | E286* | PTC | AA->TER | No |
| 4 | 115181294 | rs1045883 | AY358647 | HIT000252691 | 2430,+ | Gga/Tga | G514* | PTC | AA->TER | Yes |
| 4 | 130388368 | rs10009430 | AK091022 | HIT000015889 | 783,+ | Cga/Tga | R144* | PTC | AA->TER | Yes |
| 4 | 152570208 | rs2407221 | ENST00000309632 | HIT000118413 | 1015,+ | Taa/Gaa | *339E | Read-through | AA->TER | NA |
| 4 | 153247696 | rs493840 | BC035179 | HIT000093757 | 2215,+ | Gaa/Taa | E90* | PTC | AA->TER | No |
| 4 | 154983809 | rs5743707 | BC033756 | HIT000041939 | 2295,+ | taT/taG | Y715* | PTC | AA->TER | No |
| 4 | 156975781 | rs11554025 | Y15723 | HIT000325900 | 680,+ | Aag/Tag | K53* | PTC | AA->TER | Yes |
| 4 | 163113644 | rs7685695 | BX648713 | HIT000057360 | 2092,- | taC/taA | Y49* | PTC | AA->TER | No |
| 4 | 166319454 | rs950268 | AK093201 | HIT000018068 | 796,- | Gag/Tag | E209* | PTC | AA->TER | No |
| 4 | 174628005 | rs17849374 | CR600021 | HIT000279216 | 1460,- | Gaa/Taa | E196* | PTC | AA->TER | No |
| 4 | 183827917 | rs11730298 | AK022024 | HIT000005298 | 1924,- | tCa/tAa | S56* | PTC | AA->TER | No |
| 4 | 184793942 | rs11939152 | BC038563 | HIT000095272 | 370,- | Gaa/Taa | E46* | PTC | AA->TER | Yes |
| 4 | 185029366 | rs6821075 | BC041419 | HIT000096689 | 1086,- | Cga/Tga | R68* | PTC | TER->AA | No |
| 4 | 187497469 | rs17855071 | AK126473 | HIT000046346 | 784,+ | Gaa/Taa | E249* | PTC | AA->TER | Yes |
| 5 | 532091 | rs3777226 | AL137723 | HIT000026011 | 771,+ | tgG/tgA | W151* | PTC | AA->TER | No |

| Chr | Position | SNP ID | Accession no. | H-Inv ID | cDNA Pos. | Codon change | AA change | Effect | Inferred direction | NMD |
| --- | --- | --- | --- | --- | --- | --- | --- | --- | --- | --- |
| 5 | 1293757 | rs7447815 | AK055798 | HIT000012412 | 1080,+ | taC/taG | Y319* | PTC | AA->TER | Yes |
| 5 | 8387214 | rs7723214 | BC043282 | HIT000097600 | 967,- | Aag/Tag | K88* | PTC | TER->AA | No |
| 5 | 9239073 | rs3797936 | AF009276 | HIT000061976 | 104,+ | Gaa/Taa | E34* | PTC | AA->TER | No |
| 5 | 24206761 | rs3812094 | CR620376 | HIT000299571 | 335,- | tgG/tgA | W111* | PTC | AA->TER | No |
| 5 | 29991700 | rs9885059 | AK000016 | HIT000002491 | 395,+ | tGg/tAg | W8* | PTC | AA->TER | No |
| 5 | 32109808 | rs384728 | AB002298 | HIT000000007 | 3227,- | Gga/Tga | G947* | PTC | AA->TER | Yes |
| 5 | 32185000 | rs642354 | AF090909 | HIT000068242 | 146,- | tGg/tAg | W17* | PTC | AA->TER | No |
| 5 | 34947657 | rs2308956 | AF030933 | HIT000063046 | 762,+ | Cga/Tga | R109* | PTC | AA->TER | Yes |
| 5 | 38294701 | rs4869581 | BX647551 | HIT000056198 | 284,+ | Cga/Tga | R30* | PTC | AA->TER | Yes |
| 5 | 38566479 | rs3729732 | X61615 | HIT000322465 | 206,+ | Cga/Tga | R10* | PTC | AA->TER | Yes |
| 5 | 38919913 | rs17855842 | U60805 | HIT000220647 | 1013,+ | Gag/Tag | E216* | PTC | AA->TER | Yes |
| 5 | 52815876 | rs17404433 | BC004107 | HIT000031709 | 922,+ | tgT/tgA | C239* | PTC | AA->TER | Yes |
| 5 | 52817573 | rs11551586 | BC004107 | HIT000031709 | 1193,- | Gaa/Taa | E330* | PTC | AA->TER | No |
| 5 | 55797209 | rs16885508 | AK098579 | HIT000023409 | 670,- | taT/taG | Y123* | PTC | AA->TER | No |
| 5 | 68631690 | rs12654159 | AB024691 | HIT000058794 | 829,- | Gag/Tag | E241* | PTC | AA->TER | Yes |
| 5 | 68651835 | rs10471773 | AB024691 | HIT000058794 | 397,- | Cga/Tga | R97* | PTC | AA->TER | Yes |
| 5 | 71775699 | rs13154895 | BX647592 | HIT000056239 | 1951,- | tgC/tgA | C625* | PTC | AA->TER | No |
| 5 | 73654575 | rs12521529 | BC043537 | HIT000097732 | 509,+ | Tga/Cga | *56R | Read-through | TER->AA | NA |
| 5 | 74908475 | rs3094265 | AF163570 | HIT000072224 | 827,+ | Aga/Tga | R219* | PTC | AA->TER | Yes |

| Chr | Position | SNP ID | Accession no. | H-Inv ID | cDNA Pos. | Codon change | AA change | Effect | Inferred direction | NMD |
| --- | --- | --- | --- | --- | --- | --- | --- | --- | --- | --- |
| 5 | 75000878 | rs34358 | ENST00000344149 | HIT000136779 | 653,+ | tGg/tAg | W218* | PTC | AA->TER | Yes |
| 5 | 76394811 | rs17852651 | AY500994 | HIT000255016 | 2424,+ | tGg/tAg | W708* | PTC | AA->TER | No |
| 5 | 80947083 | rs1058210 | BC017020 | HIT000037790 | 306,+ | Gaa/Taa | E83* | PTC | AA->TER | Yes |
| 5 | 81609374 | rs11554520 | BC070221 | HIT000264179 | 95,+ | Cag/Tag | Q20* | PTC | AA->TER | Yes |
| 5 | 88054191 | rs17856763 | L08895 | HIT000191923 | 1809,- | Gga/Tga | G470* | PTC | AA->TER | No |
| 5 | 90714653 | rs3205096 | AB037797 | HIT000001091 | 225,+ | Aag/Tag | K5* | PTC | AA->TER | Yes |
| 5 | 94011015 | rs385640 | ENST00000357138 | HIT000311028 | 103,+ | Aga/Tga | R35* | PTC | AA->TER | No |
| 5 | 94846219 | rs1062020 | AB002370 | HIT000000078 | 4315,+ | Gga/Tga | G1344* | PTC | AA->TER | Yes |
| 5 | 95220349 | rs17085193 | AK096140 | HIT000020995 | 572,+ | Gaa/Taa | E54* | PTC | AA->TER | No |
| 5 | 112266057 | rs11961 | BX648005 | HIT000056652 | 395,+ | tgG/tgA | W90* | PTC | AA->TER | Yes |
| 5 | 112956678 | rs17135753 | AK095368 | HIT000020223 | 2201,- | tCa/tGa | S17* | PTC | AA->TER | No |
| 5 | 117646266 | rs11241453 | BC036254 | HIT000094321 | 99,+ | Cga/Tga | R22* | PTC | NA | Yes |
| 5 | 122067841 | rs4473766 | BC043373 | HIT000097633 | 506,- | tTa/tGa | L4* | PTC | TER->AA | Yes |
| 5 | 122163342 | rs11551069 | AK023581 | HIT000006855 | 319,+ | Gaa/Taa | E95* | PTC | AA->TER | Yes |
| 5 | 129098617 | rs2840016 | AJ311904 | HIT000247103 | 3388,+ | Gaa/Taa | E1130* | PTC | NA | Yes |
| 5 | 131614497 | rs4594848 | BC030525 | HIT000041028 | 507,- | Gag/Tag | E8* | PTC | AA->TER | No |
| 5 | 131690888 | rs11568503 | BC028313 | HIT000040520 | 1009,+ | Cga/Tga | R282* | PTC | AA->TER | Yes |
| 5 | 134810349 | rs12520799 | AK131088 | HIT000050359 | 3281,- | Aga/Tga | R117* | PTC | TER->AA | No |
| 5 | 135013292 | rs801561 | BC040891 | HIT000096428 | 1021,+ | Cag/Tag | Q81* | PTC | AA->TER | No |

| Chr | Position | SNP ID | Accession no. | H-Inv ID | cDNA Pos. | Codon change | AA change | Effect | Inferred direction | NMD |
| --- | --- | --- | --- | --- | --- | --- | --- | --- | --- | --- |
| 5 | 140011906 | rs17854679 | AJ005579 | HIT000244038 | 226,+ | Aaa/Taa | K76* | PTC | AA->TER | Yes |
| 5 | 140534494 | rs17844473 | AF217750 | HIT000074191 | 1894,+ | Gag/Tag | E632* | PTC | AA->TER | No |
| 5 | 140544105 | rs17844661 | AK056460 | HIT000013074 | 1962,+ | tCg/tAg | S596* | PTC | NA | No |
| 5 | 140552340 | rs3733689 | BC031837 | HIT000041380 | 205,- | Caa/Taa | Q11* | PTC | AA->TER | No |
| 5 | 140552728 | rs13361112 | BC031837 | HIT000041380 | 593,+ | tTa/tAa | L140* | PTC | AA->TER | No |
| 5 | 140576271 | rs584459 | AF217745 | HIT000074186 | 2392,- | Cag/Tag | Q798* | PTC | AA->TER | No |
| 5 | 140607184 | rs17844633 | AF217742 | HIT000074183 | 1854,+ | tgG/tgA | W618* | PTC | AA->TER | No |
| 5 | 142513682 | rs17287634 | AL833022 | HIT000027934 | 1209,+ | Taa/Aaa | *43K | Read-through | TER->AA | NA |
| 5 | 142579635 | rs9324909 | AL832685 | HIT000027597 | 1781,+ | tCg/tAg | S7* | PTC | TER->AA | No |
| 5 | 143180259 | rs2287660 | AF103884 | HIT000069398 | 166,+ | tCg/tAg | S33* | PTC | AA->TER | No |
| 5 | 145457953 | rs6892393 | ENST00000311450 | HIT000119061 | 215,- | tGg/tAg | W72* | PTC | AA->TER | Yes |
| 5 | 146753874 | rs17853868 | D78014 | HIT000101734 | 1740,- | Caa/Taa | Q544* | PTC | AA->TER | No |
| 5 | 149753260 | rs11541809 | U40847 | HIT000219748 | 3595,+ | Cag/Tag | Q1168* | PTC | AA->TER | Yes |
| 5 | 150387256 | rs11544082 | CR622704 | HIT000301899 | 522,+ | Aaa/Taa | K71* | PTC | AA->TER | Yes |
| 5 | 150637296 | rs13155272 | AK127978 | HIT000047851 | 1676,- | Gag/Tag | E422* | PTC | AA->TER | No |
| 5 | 156998299 | rs11952354 | AB022083 | HIT000058720 | 1692,- | tCa/tGa | S466* | PTC | AA->TER | Yes |
| 5 | 160003753 | rs3749670 | AB018258 | HIT000000440 | 1512,+ | Gag/Tag | E280* | PTC | AA->TER | Yes |
| 5 | 168948056 | rs1047000 | BC012568 | HIT000035876 | 203,+ | Cga/Tga | R20* | PTC | AA->TER | Yes |
| 5 | 175546554 | rs6556245 | BC040332 | HIT000096133 | 3275,+ | Cag/Tag | Q74* | PTC | AA->TER | No |

| Chr | Position | SNP ID | Accession no. | H-Inv ID | cDNA Pos. | Codon change | AA change | Effect | Inferred direction | NMD |
| --- | --- | --- | --- | --- | --- | --- | --- | --- | --- | --- |
| 5 | 177331574 | rs7703216 | ENST00000332215 | HIT000127657 | 196,+ | Aag/Tag | K66* | PTC | NA | No |
| 5 | 178300000 | rs13182347 | AK055745 | HIT000012359 | 691,- | tGa/tCa | *78S | Read-through | AA->TER | NA |
| 5 | 178552732 | rs2052472 | AK172763 | HIT000249926 | 557,+ | tAg/tCg | *131S | Read-through | AA->TER | NA |
| 5 | 178552784 | rs2052470 | AK172763 | HIT000249926 | 505,+ | Cga/Tga | R114* | PTC | AA->TER | No |
| 5 | 178552857 | rs4700787 | AK172763 | HIT000249926 | 432,- | tgG/tgA | W89* | PTC | AA->TER | No |
| 6 | 2726799 | rs17135709 | AK093082 | HIT000017949 | 1801,- | taC/taA | Y33* | PTC | TER->AA | Yes |
| 6 | 2779143 | rs11551131 | CR626605 | HIT000305800 | 878,+ | Gag/Tag | E280* | PTC | AA->TER | No |
| 6 | 2832944 | rs318480 | AL049245 | HIT000023691 | 298,+ | tgA/tgG | *43W | Read-through | AA->TER | NA |
| 6 | 3232117 | rs6910086 | AK096219 | HIT000021074 | 333,+ | Cga/Tga | R35* | PTC | AA->TER | No |
| 6 | 7461832 | rs2798161 | ENST00000305820 | HIT000117143 | 279,- | taC/taA | Y93* | PTC | TER->AA | No |
| 6 | 10901929 | rs6899565 | BC069277 | HIT000263430 | 791,+ | Tag/Cag | *58Q | Read-through | TER->AA | NA |
| 6 | 16504297 | rs1092076 | AK129547 | HIT000048818 | 184,+ | tTg/tAg | L38* | PTC | AA->TER | No |
| 6 | 18573689 | rs480936 | CR593698 | HIT000272893 | 765,- | Cag/Tag | Q50* | PTC | TER->AA | No |
| 6 | 22398525 | rs6238 | BC015850 | HIT000037361 | 871,+ | Cga/Tga | R117* | PTC | AA->TER | Yes |
| 6 | 25886161 | rs2328894 | AK024903 | HIT000008177 | 1416,+ | Caa/Taa | Q433* | PTC | AA->TER | Yes |
| 6 | 26794919 | rs3926558 | CR590573 | HIT000269768 | 1099,- | tgA/tgT | *72C | Read-through | AA->TER | NA |
| 6 | 27478025 | rs6456778 | BC035154 | HIT000093735 | 385,+ | Gga/Tga | G19* | PTC | TER->AA | No |
| 6 | 27969289 | rs13218959 | BX647290 | HIT000055937 | 108,+ | Aaa/Taa | K24* | PTC | AA->TER | No |
| 6 | 29177278 | rs2394517 | ENST00000358688 | HIT000312355 | 580,+ | Cag/Tag | Q194* | PTC | AA->TER | No |

| Chr | Position | SNP ID | Accession no. | H-Inv ID | cDNA Pos. | Codon change | AA change | Effect | Inferred direction | NMD |
| --- | --- | --- | --- | --- | --- | --- | --- | --- | --- | --- |
| 6 | 29515934 | rs17184009 | NM_013941 | HIT000317114 | 163,+ | Cag/Tag | Q55* | PTC | AA->TER | No |
| 6 | 29607516 | rs909967 | AK126097 | HIT000045970 | 1801,+ | tgG/tgA | W200* | PTC | AA->TER | No |
| 6 | 29608270 | rs6920668 | AK097713 | HIT000022567 | 1723,+ | tGg/tAg | W88* | PTC | AA->TER | No |
| 6 | 29868502 | rs1611212 | AF036977 | HIT000063353 | 328,- | tGg/tAg | W2* | PTC | AA->TER | No |
| 6 | 29965207 | rs2231083 | AK090500 | HIT000015367 | 1361,+ | Cag/Tag | Q46* | PTC | AA->TER | Yes |
| 6 | 30230163 | rs2517647 | AF220123 | HIT000074330 | 1082,+ | taC/taA | Y337* | PTC | AA->TER | Yes |
| 6 | 30337442 | rs2023477 | AK127349 | HIT000047222 | 846,- | tGg/tAg | W144* | PTC | AA->TER | Yes |
| 6 | 30764671 | rs11539189 | AK075509 | HIT000082542 | 889,+ | Gag/Tag | E179* | PTC | AA->TER | No |
| 6 | 30800031 | rs3873305 | BC007605 | HIT000033326 | 1349,+ | Gag/Tag | E405* | PTC | AA->TER | No |
| 6 | 30800153 | rs3873310 | BC007605 | HIT000033326 | 1471,+ | taA/taT | *445Y | Read-through | TER->AA | NA |
| 6 | 30944963 | rs3130656 | AL137624 | HIT000025912 | 1767,- | tCa/tGa | S54* | PTC | TER->AA | No |
| 6 | 31224225 | rs130068 | AK000204 | HIT000002679 | 1328,+ | Cag/Tag | Q417* | PTC | NA | Yes |
| 6 | 31471069 | rs2523465 | BC035656 | HIT000094007 | 183,+ | taC/taG | Y9* | PTC | NA | No |
| 6 | 31486338 | rs17206518 | L14848 | HIT000192269 | 149,+ | tGg/tAg | W37* | PTC | AA->TER | Yes |
| 6 | 31582150 | rs3087326 | BC044218 | HIT000052850 | 556,+ | Cga/Tga | R150* | PTC | NA | Yes |
| 6 | 32289000 | rs3131289 | U95299 | HIT000222776 | 2418,- | tgC/tgA | C776* | PTC | AA->TER | Yes |
| 6 | 32407800 | rs1003878 | U60665 | HIT000220642 | 669,+ | Cag/Tag | Q145* | PTC | AA->TER | Yes |
| 6 | 33042068 | rs11751239 | AK055186 | HIT000011800 | 570,+ | Tga/Cga | *51R | Read-through | TER->AA | NA |
| 6 | 33053680 | rs12822 | D42040 | HIT000101461 | 3199,+ | Gag/Tag | E500* | PTC | NA | Yes |

| Chr | Position | SNP ID | Accession no. | H-Inv ID | cDNA Pos. | Codon change | AA change | Effect | Inferred direction | NMD |
| --- | --- | --- | --- | --- | --- | --- | --- | --- | --- | --- |
| 6 | 33156444 | rs17418872 | CR602959 | HIT000282154 | 147,+ | Gga/Tga | G40* | PTC | NA | Yes |
| 6 | 33377520 | rs9469396 | AF029750 | HIT000062990 | 1380,- | Gag/Tag | E448* | PTC | AA->TER | No |
| 6 | 33493304 | rs11543059 | CR613274 | HIT000292469 | 214,+ | taC/taG | Y71* | PTC | AA->TER | Yes |
| 6 | 33707185 | rs11755257 | BC044947 | HIT000097993 | 1857,- | Gaa/Taa | E115* | PTC | AA->TER | No |
| 6 | 35322036 | rs28472570 | AF452494 | HIT000079868 | 2834,+ | tTa/tAa | L943* | PTC | AA->TER | No |
| 6 | 35371655 | rs8205 | CR614742 | HIT000293937 | 84,- | Aaa/Taa | K17* | PTC | AA->TER | No |
| 6 | 35394020 | rs17856543 | BC007702 | HIT000086894 | 962,+ | Gag/Tag | E321* | PTC | AA->TER | Yes |
| 6 | 35554034 | rs2235382 | AF142482 | HIT000071208 | 716,+ | Cag/Tag | Q239* | PTC | AA->TER | Yes |
| 6 | 36214735 | rs3179150 | CR603684 | HIT000282879 | 996,+ | Gag/Tag | E315* | PTC | AA->TER | Yes |
| 6 | 37643562 | rs6457999 | AK128448 | HIT000048321 | 3653,- | Cag/Tag | Q25* | PTC | AA->TER | No |
| 6 | 39464614 | rs9380853 | BC045180 | HIT000098013 | 1536,+ | Tag/Cag | *139Q | Read-through | TER->AA | NA |
| 6 | 42063307 | rs4714528 | AK097051 | HIT000021906 | 309,- | Cag/Tag | Q94* | PTC | AA->TER | Yes |
| 6 | 42168813 | rs9471759 | AK123786 | HIT000043659 | 860,+ | Cga/Tga | R61* | PTC | AA->TER | No |
| 6 | 43591343 | rs1063820 | CR590752 | HIT000269947 | 532,+ | taC/taA | Y121* | PTC | NA | Yes |
| 6 | 46003793 | rs3777562 | AK130747 | HIT000050018 | 1649,+ | tGg/tAg | W41* | PTC | AA->TER | No |
| 6 | 46298837 | rs17853843 | D83407 | HIT000101799 | 783,- | tgA/tgG | *198W | Read-through | AA->TER | NA |
| 6 | 52396865 | rs11552772 | BC012921 | HIT000087967 | 265,+ | Caa/Taa | Q76* | PTC | AA->TER | Yes |
| 6 | 53877145 | rs17425243 | AF332199 | HIT000077700 | 1140,+ | Aaa/Taa | K306* | PTC | AA->TER | Yes |
| 6 | 57575059 | rs17855928 | BC064931 | HIT000261639 | 1120,+ | taC/taG | Y347* | PTC | AA->TER | Yes |

| Chr | Position | SNP ID | Accession no. | H-Inv ID | cDNA Pos. | Codon change | AA change | Effect | Inferred direction | NMD |
| --- | --- | --- | --- | --- | --- | --- | --- | --- | --- | --- |
| 6 | 64248161 | rs6903050 | ENST00000340712 | HIT000133342 | 164,+ | tGg/tAg | W55* | PTC | AA->TER | Yes |
| 6 | 72141561 | rs9351808 | AK127397 | HIT000047270 | 260,- | taT/taA | Y12* | PTC | NA | No |
| 6 | 74139659 | rs15657 | ENST00000324538 | HIT000123879 | 49,+ | Caa/Taa | Q17* | PTC | AA->TER | No |
| 6 | 82518121 | rs1046635 | AF350451 | HIT000078211 | 736,- | Gag/Tag | E148* | PTC | AA->TER | Yes |
| 6 | 84961399 | rs2875398 | AB023226 | HIT000000726 | 971,- | Gaa/Taa | E241* | PTC | AA->TER | Yes |
| 6 | 86444607 | rs1059307 | CR617391 | HIT000296586 | 555,+ | tCa/tAa | S34* | PTC | AA->TER | Yes |
| 6 | 88124437 | rs6919193 | AK057638 | HIT000014250 | 606,- | Aaa/Taa | K61* | PTC | AA->TER | No |
| 6 | 88372398 | rs11544141 | AF125507 | HIT000070516 | 402,+ | Gag/Tag | E123* | PTC | NA | Yes |
| 6 | 88448096 | rs11666 | CR599380 | HIT000278575 | 822,+ | Cag/Tag | Q129* | PTC | AA->TER | Yes |
| 6 | 100117475 | rs11546519 | M74091 | HIT000196302 | 191,+ | Gaa/Taa | E55* | PTC | AA->TER | Yes |
| 6 | 109796883 | rs11542733 | D14043 | HIT000100533 | 537,+ | tCa/tAa | S153* | PTC | AA->TER | Yes |
| 6 | 114288245 | rs13210099 | D10522 | HIT000100429 | 1165,+ | Gag/Tag | E266* | PTC | AA->TER | No |
| 6 | 114377067 | rs1042903 | U31814 | HIT000219162 | 894,+ | tgT/tgA | C230* | PTC | TER->AA | Yes |
| 6 | 117108503 | rs1742332 | AK091073 | HIT000015940 | 113,- | Gag/Tag | E36* | PTC | AA->TER | No |
| 6 | 117256701 | rs6907580 | AF502962 | HIT000080961 | 191,- | Cga/Tga | R57* | PTC | AA->TER | Yes |
| 6 | 118930228 | rs2496353 | AF167351 | HIT000072299 | 407,+ | Tga/Aga | *91R | Read-through | TER->AA | NA |
| 6 | 119271261 | rs9372516 | CR610395 | HIT000289590 | 492,+ | Caa/Taa | Q47* | PTC | TER->AA | No |
| 6 | 132980535 | rs8192646 | AY703480 | HIT000257306 | 503,- | tGg/tAg | W168* | PTC | AA->TER | No |
| 6 | 136640702 | rs7773226 | D79986 | HIT000042301 | 1263,- | tTa/tAa | L337* | PTC | AA->TER | Yes |

| Chr | Position | SNP ID | Accession no. | H-Inv ID | cDNA Pos. | Codon change | AA change | Effect | Inferred direction | NMD |
| --- | --- | --- | --- | --- | --- | --- | --- | --- | --- | --- |
| 6 | 138775908 | rs1053656 | AK057928 | HIT000014535 | 830,+ | tgA/tgG | *206W | Read-through | TER->AA | NA |
| 6 | 139736654 | rs11541550 | U65093 | HIT000220874 | 320,+ | Cag/Tag | Q41* | PTC | AA->TER | Yes |
| 6 | 143140768 | rs11966687 | AJ227883 | HIT000245032 | 462,- | tCa/tGa | S38* | PTC | AA->TER | No |
| 6 | 144123449 | rs2207426 | AB014580 | HIT000000405 | 687,+ | Cga/Tga | R214* | PTC | AA->TER | Yes |
| 6 | 147679081 | rs28546347 | AL834152 | HIT000028883 | 1808,+ | taT/taG | Y549* | PTC | AA->TER | Yes |
| 6 | 153384423 | rs3178259 | AF129535 | HIT000070622 | 1394,+ | Cga/Tga | R445* | PTC | AA->TER | No |
| 6 | 160171926 | rs11538354 | CR613792 | HIT000292987 | 1240,+ | Cag/Tag | Q353* | PTC | AA->TER | Yes |
| 6 | 167680113 | rs2235197 | AK091987 | HIT000016854 | 627,+ | tGg/tAg | W151* | PTC | AA->TER | Yes |
| 6 | 167766826 | rs17855832 | U03399 | HIT000217498 | 105,- | Gag/Tag | E10* | PTC | AA->TER | Yes |
| 6 | 170018833 | rs17860653 | AK023224 | HIT000006498 | 550,+ | Cag/Tag | Q58* | PTC | AA->TER | No |
| 6 | 170634235 | rs17860751 | AL832504 | HIT000027416 | 1761,- | tCa/tGa | S31* | PTC | TER->AA | No |
| 7 | 1400873 | rs7800178 | BC036405 | HIT000094386 | 2038,+ | Cga/Tga | R124* | PTC | AA->TER | No |
| 7 | 2167828 | rs11551169 | BC001173 | HIT000030194 | 62,+ | Gag/Tag | E11* | PTC | AA->TER | Yes |
| 7 | 5341198 | rs11546925 | CR597103 | HIT000276298 | 1010,+ | Gag/Tag | E253* | PTC | AA->TER | Yes |
| 7 | 7091963 | rs6463665 | AK094662 | HIT000019517 | 1718,+ | taT/taG | Y30* | PTC | AA->TER | No |
| 7 | 9539908 | rs13238888 | ENST00000361014 | HIT000314305 | 799,+ | Gag/Tag | E267* | PTC | AA->TER | Yes |
| 7 | 12501612 | rs4027603 | CR614654 | HIT000293849 | 864,+ | Cag/Tag | Q165* | PTC | AA->TER | No |
| 7 | 13719365 | rs28565271 | BC045776 | HIT000098199 | 1525,- | tgA/tgC | *368C | Read-through | AA->TER | NA |
| 7 | 19972051 | rs2108292 | AK131400 | HIT000249688 | 1482,+ | tgT/tgA | C391* | PTC | AA->TER | Yes |

| Chr | Position | SNP ID | Accession no. | H-Inv ID | cDNA Pos. | Codon change | AA change | Effect | Inferred direction | NMD |
| --- | --- | --- | --- | --- | --- | --- | --- | --- | --- | --- |
| 7 | 21356203 | rs2285943 | AJ320497 | HIT000247214 | 131,+ | Gag/Tag | E34* | PTC | AA->TER | Yes |
| 7 | 23087063 | rs11537976 | X76534 | HIT000323585 | 1754,- | Gaa/Taa | E555* | PTC | AA->TER | No |
| 7 | 23334669 | rs1054421 | AB097048 | HIT000242185 | 225,+ | Gga/Tga | G23* | PTC | AA->TER | Yes |
| 7 | 24936903 | rs11548802 | AL713681 | HIT000026606 | 136,+ | Aag/Tag | K26* | PTC | AA->TER | Yes |
| 7 | 26368669 | rs740187 | BX537871 | HIT000054655 | 1391,- | Caa/Taa | Q50* | PTC | TER->AA | No |
| 7 | 30466402 | rs8192492 | AF011406 | HIT000062099 | 1301,+ | Cga/Tga | R411* | PTC | AA->TER | No |
| 7 | 30568282 | rs6966017 | AF128846 | HIT000070597 | 383,+ | Cga/Tga | R123* | PTC | AA->TER | No |
| 7 | 38052225 | rs13309540 | AK026401 | HIT000009675 | 140,- | tgG/tgA | W44* | PTC | AA->TER | No |
| 7 | 43441748 | rs7803483 | AK024255 | HIT000007529 | 1712,- | Gaa/Taa | E14* | PTC | AA->TER | No |
| 7 | 43712967 | rs2528388 | AK025360 | HIT000008634 | 971,+ | tCa/tGa | S22* | PTC | AA->TER | No |
| 7 | 43878136 | rs10250779 | BC073741 | HIT000264972 | 287,- | tGg/tAg | W78* | PTC | AA->TER | Yes |
| 7 | 55314916 | rs815957 | AK075391 | HIT000082426 | 490,- | Cga/Tga | R83* | PTC | AA->TER | No |
| 7 | 55896304 | rs11555468 | AF385084 | HIT000078722 | 290,+ | Aag/Tag | K79* | PTC | AA->TER | Yes |
| 7 | 56882922 | rs10241271 | ENST00000340814 | HIT000133444 | 129,+ | tgG/tgA | W43* | PTC | NA | Yes |
| 7 | 57081896 | rs7777285 | ENST00000297383 | HIT000114795 | 34,- | Cag/Tag | Q12* | PTC | NA | Yes |
| 7 | 63882817 | rs1404453 | M55422 | HIT000195786 | 2567,- | Tga/Cga | *428R | Read-through | AA->TER | NA |
| 7 | 65608685 | rs11766262 | AK024599 | HIT000007873 | 1168,+ | Cga/Tga | R29* | PTC | AA->TER | No |
| 7 | 65714372 | rs13308600 | AJ250042 | HIT000245962 | 993,+ | Aag/Tag | K306* | PTC | AA->TER | Yes |
| 7 | 71638515 | rs3015858 | BC068520 | HIT000263077 | 1242,- | tGg/tAg | W63* | PTC | NA | Yes |

| Chr | Position | SNP ID | Accession no. | H-Inv ID | cDNA Pos. | Codon change | AA change | Effect | Inferred direction | NMD |
| --- | --- | --- | --- | --- | --- | --- | --- | --- | --- | --- |
| 7 | 71863659 | rs17145837 | AF416611 | HIT000079299 | 1476,- | tCa/tGa | S264* | PTC | NA | No |
| 7 | 75266478 | rs17856838 | BC051850 | HIT000053711 | 219,- | Cag/Tag | Q44* | PTC | AA->TER | Yes |
| 7 | 75581700 | rs2908197 | AK092550 | HIT000017417 | 1442,- | tGg/tAg | W67* | PTC | AA->TER | No |
| 7 | 77632361 | rs17856775 | BC027852 | HIT000040313 | 730,+ | Cga/Tga | R98* | PTC | AA->TER | No |
| 7 | 79945100 | rs3211938 | M98399 | HIT000197016 | 1229,+ | taT/taG | Y325* | PTC | AA->TER | Yes |
| 7 | 79948001 | rs1803256 | M98399 | HIT000197016 | 1560,+ | Gga/Tga | G436* | PTC | AA->TER | Yes |
| 7 | 80999934 | rs5745703 | M73240 | HIT000196260 | 1141,+ | tGg/tAg | W359* | PTC | AA->TER | Yes |
| 7 | 89499300 | rs28945132 | AY008443 | HIT000250650 | 440,+ | Aaa/Taa | K85* | PTC | AA->TER | Yes |
| 7 | 94582090 | rs3917594 | S64615 | HIT000216072 | 591,+ | tgG/tgA | W194* | PTC | AA->TER | Yes |
| 7 | 97007734 | rs11554502 | U37529 | HIT000219523 | 317,+ | Aga/Tga | R58* | PTC | AA->TER | Yes |
| 7 | 97128562 | rs17400565 | BC008723 | HIT000033923 | 1575,+ | Cga/Tga | R407* | PTC | NA | Yes |
| 7 | 97166371 | rs2394771 | AB007953 | HIT000000200 | 2449,- | Tag/Gag | *51E | Read-through | NA | NA |
| 7 | 98666090 | rs11555144 | AB014532 | HIT000000357 | 1961,+ | Cag/Tag | Q627* | PTC | AA->TER | No |
| 7 | 98879550 | rs10229552 | AK124190 | HIT000044063 | 2000,+ | tgT/tgA | C18* | PTC | TER->AA | No |
| 7 | 99855219 | rs1053507 | CR609640 | HIT000288835 | 356,+ | Cga/Tga | R52* | PTC | AA->TER | Yes |
| 7 | 100016009 | rs2293766 | AF332976 | HIT000077753 | 5814,- | tgG/tgA | W1883* | PTC | AA->TER | Yes |
| 7 | 100136201 | rs1056867 | M55040 | HIT000195763 | 460,+ | Caa/Taa | Q102* | PTC | NA | Yes |
| 7 | 101708539 | rs11157 | BC065711 | HIT000261992 | 359,+ | Gag/Tag | E103* | PTC | NA | No |
| 7 | 102496614 | rs4729909 | ENST00000257741 | HIT000107148 | 217,+ | Cag/Tag | Q73* | PTC | AA->TER | No |

| Chr | Position | SNP ID | Accession no. | H-Inv ID | cDNA Pos. | Codon change | AA change | Effect | Inferred direction | NMD |
| --- | --- | --- | --- | --- | --- | --- | --- | --- | --- | --- |
| 7 | 102864505 | rs3025966 | U79716 | HIT000221548 | 2710,- | taT/taG | Y845* | PTC | AA->TER | Yes |
| 7 | 103902174 | rs6969029 | ENST00000330383 | HIT000126649 | 28,+ | Aag/Tag | K10* | PTC | NA | Yes |
| 7 | 104786013 | rs11556987 | BC068483 | HIT000263040 | 1611,+ | tGg/tAg | W460* | PTC | AA->TER | Yes |
| 7 | 107166734 | rs3177962 | M61916 | HIT000196005 | 4345,+ | Gaa/Taa | E1410* | PTC | AA->TER | Yes |
| 7 | 122433918 | rs28364172 | AF260824 | HIT000075286 | 59,+ | Cga/Tga | R12* | PTC | AA->TER | Yes |
| 7 | 122730815 | rs17853096 | BC026173 | HIT000091209 | 1288,- | Cag/Tag | Q374* | PTC | AA->TER | Yes |
| 7 | 126845103 | rs2233584 | AF043978 | HIT000063925 | 1204,+ | tGg/tAg | W333* | PTC | AA->TER | No |
| 7 | 127888300 | rs10227728 | BC062355 | HIT000260355 | 2006,+ | Cag/Tag | Q39* | PTC | NA | No |
| 7 | 128003172 | rs11545531 | AK056338 | HIT000012952 | 1803,+ | tgA/tgG | *324W | Read-through | TER->AA | NA |
| 7 | 128427579 | rs9801442 | AK024098 | HIT000007372 | 180,+ | Aaa/Taa | K41* | PTC | NA | No |
| 7 | 130069798 | rs17165272 | CR618431 | HIT000297626 | 753,+ | Cga/Tga | R47* | PTC | AA->TER | No |
| 7 | 130625769 | rs2398788 | AK023427 | HIT000006701 | 2502,- | tCa/tGa | S72* | PTC | AA->TER | No |
| 7 | 132177527 | rs11549635 | CR601494 | HIT000280689 | 410,+ | Cga/Tga | R137* | PTC | AA->TER | No |
| 7 | 132177752 | rs11549636 | CR601494 | HIT000280689 | 185,+ | Gag/Tag | E62* | PTC | AA->TER | No |
| 7 | 136990471 | rs881505 | BC047556 | HIT000098659 | 1441,- | taC/taG | Y83* | PTC | AA->TER | No |
| 7 | 138212466 | rs10280498 | AF085913 | HIT000066792 | 237,- | tgG/tgA | W30* | PTC | AA->TER | No |
| 7 | 138534572 | rs4732371 | AF318327 | HIT000077254 | 1525,+ | tTa/tAa | L80* | PTC | AA->TER | No |
| 7 | 139938673 | rs13243144 | AJ276310 | HIT000246327 | 124,- | Gga/Tga | G42* | PTC | AA->TER | No |
| 7 | 140806049 | rs6943245 | BX640861 | HIT000055408 | 2464,- | tGg/tAg | W18* | PTC | AA->TER | No |

| Chr | Position | SNP ID | Accession no. | H-Inv ID | cDNA Pos. | Codon change | AA change | Effect | Inferred direction | NMD |
| --- | --- | --- | --- | --- | --- | --- | --- | --- | --- | --- |
| 7 | 141422844 | rs4726514 | BC047233 | HIT000098488 | 496,- | Tga/Aga | *166R | Read-through | NA | NA |
| 7 | 141793587 | rs17284 | L26054 | HIT000192655 | 150,+ | taT/taG | Y50* | PTC | AA->TER | No |
| 7 | 142027287 | rs17267 | D13086 | HIT000100480 | 163,- | Cga/Tga | R55* | PTC | AA->TER | No |
| 7 | 142604610 | rs1140426 | BC008743 | HIT000033938 | 1839,+ | Tga/Cga | *573R | Read-through | TER->AA | NA |
| 7 | 148923635 | rs893597 | AB111888 | HIT000242330 | 1976,- | tgT/tgA | C280* | PTC | AA->TER | Yes |
| 7 | 148930421 | rs28687295 | AB111888 | HIT000242330 | 4689,+ | Cga/Tga | R1185* | PTC | AA->TER | Yes |
| 7 | 148960014 | rs2074697 | AK123170 | HIT000043043 | 1006,+ | tGa/tTa | *331L | Read-through | TER->AA | NA |
| 7 | 149857345 | rs1009861 | CR612038 | HIT000291233 | 653,- | Cga/Tga | R20* | PTC | AA->TER | No |
| 7 | 151264218 | rs758683 | AK022341 | HIT000005615 | 1514,+ | Gag/Tag | E125* | PTC | AA->TER | No |
| 7 | 151358762 | rs4024337 | AY024361 | HIT000083219 | 3489,+ | tgT/tgA | C1103* | PTC | AA->TER | Yes |
| 7 | 151358797 | rs4024370 | AY024361 | HIT000083219 | 3454,+ | Cga/Tga | R1092* | PTC | NA | Yes |
| 7 | 151431787 | rs12671251 | BX538090 | HIT000054874 | 603,- | tgT/tgA | C64* | PTC | NA | No |
| 7 | 154205331 | rs1058833 | AK123600 | HIT000043473 | 979,+ | tGg/tAg | W232* | PTC | AA->TER | Yes |
| 7 | 154297488 | rs2581842 | BC031272 | HIT000092335 | 1612,+ | Caa/Taa | Q51* | PTC | AA->TER | No |
| 7 | 154527669 | rs11544449 | BC001880 | HIT000030716 | 175,+ | tGg/tAg | W9* | PTC | AA->TER | Yes |
| 7 | 157265066 | rs13311810 | ENST00000357228 | HIT000311101 | 755,+ | Taa/Gaa | *195E | Read-through | AA->TER | NA |
| 8 | 460861 | rs7009270 | AL833531 | HIT000028443 | 2164,- | tCa/tAa | S11* | PTC | AA->TER | No |
| 8 | 460889 | rs9772729 | AL833531 | HIT000028443 | 2136,- | Caa/Taa | Q2* | PTC | AA->TER | No |
| 8 | 599880 | rs12675823 | AK128318 | HIT000048191 | 1741,- | tGg/tAg | W28* | PTC | AA->TER | Yes |

| Chr | Position | SNP ID | Accession no. | H-Inv ID | cDNA Pos. | Codon change | AA change | Effect | Inferred direction | NMD |
| --- | --- | --- | --- | --- | --- | --- | --- | --- | --- | --- |
| 8 | 1695454 | rs730378 | BC025336 | HIT000091044 | 567,- | taT/taG | Y84* | PTC | AA->TER | No |
| 8 | 6250491 | rs1057187 | CR625518 | HIT000304713 | 157,- | Gaa/Taa | E40* | PTC | AA->TER | No |
| 8 | 6804222 | rs2738079 | AY746434 | HIT000257441 | 284,+ | tGa/tTa | *67L | Read-through | TER->AA | NA |
| 8 | 11656913 | rs809203 | AK055534 | HIT000012148 | 740,- | tgT/tgA | C194* | PTC | AA->TER | No |
| 8 | 11815310 | rs7464553 | ENST00000319916 | HIT000122237 | 837,- | tgA/tgG | *279W | Read-through | AA->TER | NA |
| 8 | 11910132 | rs11784716 | AK091259 | HIT000016126 | 1073,+ | tgG/tgA | W236* | PTC | NA | Yes |
| 8 | 12378899 | rs4096460 | AY461701 | HIT000254807 | 558,+ | Taa/Caa | *58Q | Read-through | TER->AA | NA |
| 8 | 12454624 | rs10107331 | AK091914 | HIT000016781 | 1054,- | Tag/Cag | *78Q | Read-through | TER->AA | NA |
| 8 | 12454773 | rs2970282 | AK091914 | HIT000016781 | 905,+ | tGg/tAg | W28* | PTC | NA | No |
| 8 | 12480423 | rs2681168 | AK092544 | HIT000017411 | 238,+ | tTg/tAg | L30* | PTC | NA | No |
| 8 | 12586227 | rs28455527 | ENST00000317826 | HIT000121447 | 831,- | taT/taG | Y277* | PTC | AA->TER | No |
| 8 | 18512199 | rs6988287 | CR936668 | HIT000306941 | 500,- | taC/taA | Y51* | PTC | AA->TER | No |
| 8 | 19864004 | rs328 | M15856 | HIT000194421 | 1595,+ | tCa/tGa | S474* | PTC | AA->TER | No |
| 8 | 23279414 | rs13252670 | AL833246 | HIT000028158 | 4205,+ | tGg/tAg | W125* | PTC | AA->TER | No |
| 8 | 26304780 | rs1055806 | AF452712 | HIT000079871 | 302,+ | Gga/Tga | G69* | PTC | AA->TER | Yes |
| 8 | 27735869 | rs1675236 | BC015191 | HIT000037045 | 529,- | taT/taG | Y117* | PTC | AA->TER | Yes |
| 8 | 31058190 | rs17847577 | AF091214 | HIT000068318 | 1336,+ | Cga/Tga | R369* | PTC | AA->TER | Yes |
| 8 | 31150077 | rs11574410 | AF091214 | HIT000068318 | 4447,+ | Cga/Tga | R1406* | PTC | AA->TER | No |
| 8 | 36783039 | rs4130197 | BC028701 | HIT000040612 | 591,+ | tTg/tAg | L188* | PTC | AA->TER | Yes |

| Chr | Position | SNP ID | Accession no. | H-Inv ID | cDNA Pos. | Codon change | AA change | Effect | Inferred direction | NMD |
| --- | --- | --- | --- | --- | --- | --- | --- | --- | --- | --- |
| 8 | 55212514 | rs1062907 | BC000891 | HIT000029981 | 460,+ | Aaa/Taa | K133* | PTC | AA->TER | Yes |
| 8 | 56529821 | rs2939639 | ENST00000341364 | HIT000133994 | 432,- | taT/taA | Y140* | PTC | AA->TER | No |
| 8 | 57125768 | rs6997135 | ENST00000317182 | HIT000121192 | 562,- | Cga/Tga | R185* | PTC | AA->TER | No |
| 8 | 64161128 | rs13257608 | BC041784 | HIT000096856 | 25,- | Gag/Tag | E3* | PTC | AA->TER | Yes |
| 8 | 67504098 | rs11556131 | BC013043 | HIT000036098 | 277,+ | Gag/Tag | E60* | PTC | AA->TER | No |
| 8 | 71178905 | rs12056878 | ENST00000334932 | HIT000129260 | 64,- | Cag/Tag | Q22* | PTC | AA->TER | No |
| 8 | 89115675 | rs7837474 | AL136588 | HIT000025184 | 232,- | tTg/tAg | L58* | PTC | AA->TER | No |
| 8 | 91698054 | rs12548741 | AL833064 | HIT000027976 | 1210,- | Tag/Cag | *91Q | Read-through | TER->AA | NA |
| 8 | 95973419 | rs28399560 | ENST00000319012 | HIT000121901 | 465,- | Caa/Taa | Q94* | PTC | AA->TER | No |
| 8 | 98788085 | rs3211326 | AF501310 | HIT000080935 | 1462,+ | tgG/tgA | W401* | PTC | AA->TER | Yes |
| 8 | 103909979 | rs3207798 | BC013420 | HIT000036265 | 2051,+ | Gaa/Taa | E447* | PTC | AA->TER | No |
| 8 | 113370774 | rs7001415 | AB114605 | HIT000242379 | 9195,- | tgT/tgA | C3008* | PTC | AA->TER | Yes |
| 8 | 124582502 | rs13252106 | AK023391 | HIT000006665 | 794,- | Cag/Tag | Q28* | PTC | AA->TER | No |
| 8 | 125597417 | rs11542671 | BC007066 | HIT000033025 | 280,+ | Aga/Tga | R80* | PTC | AA->TER | Yes |
| 8 | 131165286 | rs7816255 | AK054840 | HIT000011454 | 489,- | Cga/Tga | R108* | PTC | TER->AA | No |
| 8 | 133969581 | rs12543299 | X05615 | HIT000321143 | 2388,+ | Gag/Tag | E783* | PTC | AA->TER | Yes |
| 8 | 136315840 | rs7008933 | BC045817 | HIT000098236 | 285,+ | Gag/Tag | E53* | PTC | AA->TER | Yes |
| 8 | 142320549 | rs12543658 | AL832938 | HIT000027850 | 1001,- | Cga/Tga | R97* | PTC | TER->AA | No |
| 8 | 142527235 | rs6578185 | AK125848 | HIT000045721 | 2854,- | Cag/Tag | Q925* | PTC | AA->TER | Yes |

| Chr | Position | SNP ID | Accession no. | H-Inv ID | cDNA Pos. | Codon change | AA change | Effect | Inferred direction | NMD |
| --- | --- | --- | --- | --- | --- | --- | --- | --- | --- | --- |
| 8 | 143308760 | rs11988455 | AK055726 | HIT000012340 | 1648,- | Cga/Tga | R512* | PTC | AA->TER | No |
| 8 | 144946412 | rs7465554 | D63481 | HIT000042284 | 4482,- | Gag/Tag | E1494* | PTC | AA->TER | Yes |
| 8 | 145610616 | rs4319139 | AK025537 | HIT000008811 | 1087,- | taC/taG | Y329* | PTC | AA->TER | Yes |
| 8 | 145612200 | rs4355816 | AK025537 | HIT000008811 | 376,- | taC/taA | Y92* | PTC | AA->TER | Yes |
| 8 | 145707353 | rs17850833 | CR620962 | HIT000300157 | 1491,+ | Tga/Cga | *413R | Read-through | TER->AA | NA |
| 9 | 594856 | rs903219 | AK022398 | HIT000005672 | 445,+ | tTa/tGa | L47* | PTC | AA->TER | No |
| 9 | 961066 | rs604973 | BC029932 | HIT000091952 | 631,- | Cga/Tga | R75* | PTC | TER->AA | No |
| 9 | 2106029 | rs10137 | X72889 | HIT000323354 | 3874,+ | Gag/Tag | E1218* | PTC | AA->TER | Yes |
| 9 | 2164729 | rs7023098 | AK130216 | HIT000049487 | 196,- | tAg/tCg | *21S | Read-through | AA->TER | NA |
| 9 | 5116414 | rs3925597 | AF005216 | HIT000061792 | 3753,- | Aga/Tga | R1087* | PTC | AA->TER | No |
| 9 | 5290316 | rs3174295 | X00948 | HIT000320881 | 340,- | Caa/Taa | Q114* | PTC | AA->TER | No |
| 9 | 21197037 | rs10119910 | V00551 | HIT000320831 | 106,- | tgT/tgA | C20* | PTC | NA | No |
| 9 | 21471483 | rs2039381 | AY358570 | HIT000252614 | 831,+ | Cag/Tag | Q71* | PTC | AA->TER | No |
| 9 | 27368301 | rs1330920 | AK074231 | HIT000015011 | 642,+ | Cga/Tga | R145* | PTC | TER->AA | No |
| 9 | 33497865 | rs7861182 | NM_001007550 | HIT000316466 | 260,- | Cag/Tag | Q85* | PTC | AA->TER | Yes |
| 9 | 34362873 | rs4879782 | BC070098 | HIT000264056 | 205,- | taC/taG | Y23* | PTC | AA->TER | No |
| 9 | 35173423 | rs13290189 | AK128829 | HIT000048702 | 541,+ | Gga/Tga | G152* | PTC | NA | No |
| 9 | 35173801 | rs10120995 | AK128829 | HIT000048702 | 919,+ | Cga/Tga | R278* | PTC | NA | No |
| 9 | 35174125 | rs4879881 | AK128829 | HIT000048702 | 1243,+ | Tga/Aga | *386R | Read-through | NA | NA |

| Chr | Position | SNP ID | Accession no. | H-Inv ID | cDNA Pos. | Codon change | AA change | Effect | Inferred direction | NMD |
| --- | --- | --- | --- | --- | --- | --- | --- | --- | --- | --- |
| 9 | 35726556 | rs11996 | AF211848 | HIT000074029 | 1387,+ | Cag/Tag | Q317* | PTC | AA->TER | No |
| 9 | 36073430 | rs1046658 | D50406 | HIT000101564 | 617,+ | Cga/Tga | R170* | PTC | AA->TER | Yes |
| 9 | 36573668 | rs13440297 | D79997 | HIT000042312 | 273,+ | Gag/Tag | E35* | PTC | AA->TER | Yes |
| 9 | 36661008 | rs2068826 | D79997 | HIT000042312 | 1689,- | Gaa/Taa | E507* | PTC | AA->TER | Yes |
| 9 | 37755816 | rs12343460 | AK095214 | HIT000020069 | 1695,- | Caa/Taa | Q304* | PTC | NA | No |
| 9 | 37877700 | rs1054566 | BC008500 | HIT000033834 | 1059,+ | tGg/tAg | W283* | PTC | AA->TER | Yes |
| 9 | 43009203 | rs2809007 | BX538241 | HIT000055023 | 2243,+ | Cag/Tag | Q86* | PTC | NA | No |
| 9 | 44371554 | rs2658377 | AK124122 | HIT000043995 | 1460,+ | tgG/tgA | W43* | PTC | AA->TER | No |
| 9 | 64138291 | rs7040398 | BC065527 | HIT000261934 | 1445,- | Cga/Tga | R222* | PTC | NA | No |
| 9 | 64138881 | rs12380199 | BC065527 | HIT000261934 | 855,- | tCg/tAg | S25* | PTC | NA | No |
| 9 | 64234839 | rs7018660 | AK000451 | HIT000002926 | 345,- | tGg/tAg | W25* | PTC | NA | Yes |
| 9 | 65011990 | rs28671132 | BC084570 | HIT000266078 | 288,- | Cga/Tga | R44* | PTC | NA | No |
| 9 | 67941425 | rs11263209 | AK092175 | HIT000017042 | 1924,- | Cga/Tga | R65* | PTC | AA->TER | No |
| 9 | 68147743 | rs7034645 | AY344641 | HIT000252030 | 407,+ | Aag/Tag | K108* | PTC | NA | No |
| 9 | 68148201 | rs7021123 | AY344641 | HIT000252030 | 865,+ | taC/taA | Y260* | PTC | NA | No |
| 9 | 69097360 | rs10122717 | BC027592 | HIT000040277 | 3606,+ | Cag/Tag | Q1133* | PTC | AA->TER | No |
| 9 | 74749128 | rs2031780 | AK126029 | HIT000045902 | 446,+ | tAg/tCg | *123S | Read-through | TER->AA | NA |
| 9 | 76347686 | rs656106 | M97347 | HIT000196956 | 1054,+ | Gaa/Taa | E279* | PTC | AA->TER | No |
| 9 | 78880895 | rs13294961 | ENST00000331400 | HIT000127211 | 1246,- | Aaa/Taa | K416* | PTC | AA->TER | No |

| Chr | Position | SNP ID | Accession no. | H-Inv ID | cDNA Pos. | Codon change | AA change | Effect | Inferred direction | NMD |
| --- | --- | --- | --- | --- | --- | --- | --- | --- | --- | --- |
| 9 | 83712307 | rs1058995 | AY237536 | HIT000251149 | 2924,- | tGg/tAg | W927* | PTC | NA | Yes |
| 9 | 84846086 | rs923559 | AK127447 | HIT000047320 | 287,- | tgG/tgA | W41* | PTC | AA->TER | No |
| 9 | 88312849 | rs11142308 | AF087864 | HIT000067492 | 559,+ | Cga/Tga | R122* | PTC | AA->TER | Yes |
| 9 | 92152461 | rs7467077 | AB105104 | HIT000242293 | 2827,- | Gaa/Taa | E842* | PTC | AA->TER | Yes |
| 9 | 92726882 | rs3957502 | AY726558 | HIT000257342 | 621,+ | Tga/Cga | *78R | Read-through | NA | NA |
| 9 | 94965616 | rs28565379 | BC034271 | HIT000093373 | 850,+ | Cga/Tga | R87* | PTC | AA->TER | No |
| 9 | 97970010 | rs12375882 | BC011690 | HIT000035377 | 1250,- | Cga/Tga | R390* | PTC | AA->TER | Yes |
| 9 | 98253510 | rs10986142 | AL359585 | HIT000026313 | 2187,- | tGg/tAg | W20* | PTC | AA->TER | No |
| 9 | 101151170 | rs3209400 | BC022465 | HIT000039560 | 640,+ | Caa/Taa | Q170* | PTC | AA->TER | Yes |
| 9 | 108885644 | rs575200 | AK022164 | HIT000005438 | 954,+ | Cag/Tag | Q47* | PTC | AA->TER | No |
| 9 | 112839074 | rs10981589 | AK095843 | HIT000020698 | 2260,- | Cga/Tga | R341* | PTC | AA->TER | No |
| 9 | 114429700 | rs11539653 | CR607789 | HIT000286984 | 56,+ | Cag/Tag | Q10* | PTC | AA->TER | Yes |
| 9 | 114632324 | rs7027251 | AF520785 | HIT000081158 | 840,- | Aaa/Taa | K240* | PTC | AA->TER | No |
| 9 | 121289281 | rs10985245 | AF378123 | HIT000078593 | 1020,- | Gag/Tag | E50* | PTC | AA->TER | No |
| 9 | 122470795 | rs1476860 | NM_001004450 | HIT000315904 | 574,- | Cga/Tga | R192* | PTC | AA->TER | No |
| 9 | 122697104 | rs1053762 | AL833177 | HIT000028089 | 2826,+ | Aga/Tga | R259* | PTC | AA->TER | Yes |
| 9 | 126647386 | rs873837 | AK092271 | HIT000017138 | 121,- | Gaa/Taa | E12* | PTC | AA->TER | No |
| 9 | 128800684 | rs17432888 | D79991 | HIT000042306 | 411,+ | tTa/tGa | L137* | PTC | AA->TER | Yes |
| 9 | 128990483 | rs17486583 | CR620812 | HIT000300007 | 269,- | Cga/Tga | R76* | PTC | AA->TER | No |

| Chr | Position | SNP ID | Accession no. | H-Inv ID | cDNA Pos. | Codon change | AA change | Effect | Inferred direction | NMD |
| --- | --- | --- | --- | --- | --- | --- | --- | --- | --- | --- |
| 9 | 132575624 | rs13287671 | AF142328 | HIT000071196 | 337,+ | Gag/Tag | E29* | PTC | AA->TER | Yes |
| 9 | 132976081 | rs13287310 | X54457 | HIT000321904 | 1728,+ | taT/taA | Y549* | PTC | NA | Yes |
| 9 | 132986671 | rs487561 | ENST00000314220 | HIT000120059 | 170,- | tCa/tAa | S57* | PTC | TER->AA | No |
| 9 | 133507605 | rs3025342 | CR592354 | HIT000271549 | 859,- | Aga/Tga | R47* | PTC | AA->TER | No |
| 9 | 133550090 | rs7859358 | AF129264 | HIT000070605 | 1311,- | Cga/Tga | R72* | PTC | NA | No |
| 9 | 134916966 | rs3811156 | AK021598 | HIT000004872 | 277,- | Cag/Tag | Q26* | PTC | TER->AA | No |
| 9 | 135693711 | rs783768 | AK094119 | HIT000018974 | 513,+ | Taa/Gaa | *119E | Read-through | TER->AA | NA |
| 9 | 137154665 | rs4880082 | BC042845 | HIT000097371 | 1053,+ | Caa/Taa | Q157* | PTC | NA | Yes |
| 9 | 137412829 | rs11545611 | AK026594 | HIT000009868 | 396,+ | Gaa/Taa | E108* | PTC | AA->TER | No |
| 9 | 137437571 | rs11531963 | AK091056 | HIT000015923 | 1484,- | taC/taG | Y427* | PTC | AA->TER | Yes |
| 9 | 137465682 | rs28722654 | BC027323 | HIT000091323 | 329,- | tAg/tCg | *86S | Read-through | TER->AA | NA |
| 9 | 137648402 | rs7034073 | AK125060 | HIT000044933 | 2915,- | Cga/Tga | R910* | PTC | NA | Yes |
| 10 | 1053165 | rs11541699 | BC038975 | HIT000052100 | 1935,+ | Tag/Cag | *635Q | Read-through | TER->AA | NA |
| 10 | 1055710 | rs1044261 | AK056950 | HIT000013564 | 496,- | tGg/tAg | W144* | PTC | AA->TER | No |
| 10 | 1127916 | rs10903363 | AK093745 | HIT000018612 | 1822,+ | Cga/Tga | R106* | PTC | TER->AA | No |
| 10 | 3160982 | rs2279206 | AK126153 | HIT000046026 | 229,- | tGg/tAg | W30* | PTC | AA->TER | No |
| 10 | 3812317 | rs17855372 | BC000311 | HIT000029508 | 967,- | Aaa/Taa | K261* | PTC | AA->TER | No |
| 10 | 4199856 | rs7069837 | AL137435 | HIT000025723 | 672,+ | Caa/Taa | Q79* | PTC | TER->AA | No |
| 10 | 6126159 | rs12722511 | CR623776 | HIT000302971 | 407,- | Cga/Tga | R5* | PTC | AA->TER | No |

| Chr | Position | SNP ID | Accession no. | H-Inv ID | cDNA Pos. | Codon change | AA change | Effect | Inferred direction | NMD |
| --- | --- | --- | --- | --- | --- | --- | --- | --- | --- | --- |
| 10 | 10145364 | rs2657519 | BC032914 | HIT000092870 | 468,- | Caa/Taa | Q27* | PTC | AA->TER | No |
| 10 | 11131194 | rs6602474 | AK094292 | HIT000019147 | 1553,+ | tTa/tAa | L34* | PTC | NA | No |
| 10 | 13799982 | rs3814667 | AK024406 | HIT000007680 | 339,+ | Cga/Tga | R41* | PTC | TER->AA | No |
| 10 | 14077484 | rs4750451 | AK130427 | HIT000049698 | 257,- | Tag/Cag | *69Q | Read-through | TER->AA | NA |
| 10 | 15186945 | rs4262621 | AK025065 | HIT000008339 | 1768,- | Cga/Tga | R41* | PTC | TER->AA | No |
| 10 | 16603004 | rs613731 | ENST00000298943 | HIT000115228 | 67,+ | Gag/Tag | E23* | PTC | AA->TER | Yes |
| 10 | 17432281 | rs973091 | AK127986 | HIT000047859 | 348,+ | Cga/Tga | R6* | PTC | AA->TER | No |
| 10 | 27727231 | rs17560874 | AK126025 | HIT000045898 | 2420,- | Caa/Taa | Q768* | PTC | AA->TER | No |
| 10 | 29228116 | rs1621761 | ENST00000277685 | HIT000111603 | 307,- | Cga/Tga | R103* | PTC | NA | No |
| 10 | 30350707 | rs2105330 | BC018690 | HIT000089682 | 503,- | tAg/tGg | *82W | Read-through | AA->TER | NA |
| 10 | 30764590 | rs8176958 | BC022398 | HIT000090598 | 279,+ | Cga/Tga | R54* | PTC | AA->TER | No |
| 10 | 44627619 | rs17157049 | AK056518 | HIT000013132 | 2160,- | Cga/Tga | R10* | PTC | AA->TER | No |
| 10 | 46382101 | rs481271 | AK127436 | HIT000047309 | 1888,+ | Cga/Tga | R434* | PTC | NA | No |
| 10 | 51488882 | rs2338057 | AK056375 | HIT000012989 | 1555,- | Aag/Tag | K81* | PTC | NA | Yes |
| 10 | 52111604 | rs12241969 | ENST00000340638 | HIT000133268 | 946,+ | Cga/Tga | R301* | PTC | NA | Yes |
| 10 | 52113281 | rs7097507 | ENST00000340638 | HIT000133268 | 1209,+ | taC/taA | Y388* | PTC | NA | Yes |
| 10 | 60664157 | rs7907875 | CR749429 | HIT000306460 | 489,+ | tCa/tAa | S19* | PTC | AA->TER | Yes |
| 10 | 63806188 | rs11545586 | AJ505149 | HIT000248316 | 507,+ | tCa/tAa | S77* | PTC | AA->TER | Yes |
| 10 | 69342349 | rs1063114 | AF235040 | HIT000074724 | 1518,+ | tgT/tgA | C490* | PTC | AA->TER | Yes |

| Chr | Position | SNP ID | Accession no. | H-Inv ID | cDNA Pos. | Codon change | AA change | Effect | Inferred direction | NMD |
| --- | --- | --- | --- | --- | --- | --- | --- | --- | --- | --- |
| 10 | 73634249 | rs11000217 | AK094170 | HIT000019025 | 335,- | tCa/tGa | S78* | PTC | AA->TER | Yes |
| 10 | 76155652 | rs839702 | AK055114 | HIT000011728 | 770,+ | Cga/Tga | R3* | PTC | AA->TER | No |
| 10 | 78331981 | rs16934000 | BC030624 | HIT000092177 | 657,+ | tgC/tgA | C9* | PTC | NA | No |
| 10 | 82200359 | rs1870146 | BC011242 | HIT000087613 | 250,- | tgG/tgA | W5* | PTC | NA | No |
| 10 | 85982478 | rs12249354 | AB031547 | HIT000059036 | 1079,- | Gaa/Taa | E353* | PTC | AA->TER | No |
| 10 | 90685020 | rs11202904 | CR590615 | HIT000269810 | 1506,- | tgG/tgA | W358* | PTC | AA->TER | No |
| 10 | 91057112 | rs17856959 | BC032839 | HIT000051072 | 1497,+ | tgG/tgA | W473* | PTC | TER->AA | No |
| 10 | 91495654 | rs12771753 | AB033337 | HIT000059087 | 4099,+ | Aaa/Taa | K1352* | PTC | AA->TER | Yes |
| 10 | 91728805 | rs11812465 | ENST00000277860 | HIT000111624 | 352,+ | Tag/Cag | *118Q | Read-through | TER->AA | NA |
| 10 | 92670013 | rs1130407 | BC018667 | HIT000038394 | 164,+ | Gaa/Taa | E34* | PTC | AA->TER | Yes |
| 10 | 94418694 | rs7914114 | ENST00000310692 | HIT000118787 | 787,- | Cag/Tag | Q263* | PTC | AA->TER | No |
| 10 | 98500341 | rs3789959 | ENST00000277974 | HIT000111648 | 307,- | Aag/Tag | K103* | PTC | AA->TER | No |
| 10 | 98903618 | rs2784920 | AK097919 | HIT000022773 | 827,- | Gag/Tag | E35* | PTC | TER->AA | No |
| 10 | 101581375 | rs17222547 | U63970 | HIT000220793 | 2938,+ | taC/taA | Y967* | PTC | AA->TER | Yes |
| 10 | 102046006 | rs7904983 | BC025665 | HIT000039945 | 1299,- | Cga/Tga | R407* | PTC | AA->TER | Yes |
| 10 | 103444696 | rs12773760 | AF281859 | HIT000076129 | 311,- | Gga/Tga | G79* | PTC | AA->TER | Yes |
| 10 | 104173442 | rs17855346 | BC000262 | HIT000029470 | 760,- | Tag/Gag | *227E | Read-through | TER->AA | NA |
| 10 | 104404926 | rs1063241 | AF281046 | HIT000076100 | 775,+ | Aag/Tag | K256* | PTC | AA->TER | Yes |
| 10 | 118387874 | rs17856158 | BC005989 | HIT000032574 | 1071,+ | tgG/tgA | W357* | PTC | AA->TER | Yes |

| Chr | Position | SNP ID | Accession no. | H-Inv ID | cDNA Pos. | Codon change | AA change | Effect | Inferred direction | NMD |
| --- | --- | --- | --- | --- | --- | --- | --- | --- | --- | --- |
| 10 | 118419753 | rs7903750 | BC034627 | HIT000093514 | 150,+ | Tag/Cag | *50Q | Read-through | TER->AA | NA |
| 10 | 124204345 | rs2736911 | BC066349 | HIT000262240 | 177,+ | Cga/Tga | R38* | PTC | AA->TER | Yes |
| 10 | 124239094 | rs17852904 | CR597732 | HIT000276927 | 805,+ | Gag/Tag | E247* | PTC | AA->TER | Yes |
| 10 | 126216761 | rs897284 | AK127935 | HIT000047808 | 774,- | tGg/tAg | W155* | PTC | AA->TER | No |
| 10 | 126717592 | rs3208568 | AL833398 | HIT000028310 | 422,+ | Aaa/Taa | K8* | PTC | AA->TER | Yes |
| 10 | 127921373 | rs2242388 | CR621992 | HIT000301187 | 1139,+ | tgA/tgC | *85C | Read-through | TER->AA | NA |
| 10 | 134638301 | rs12241497 | AK125849 | HIT000045722 | 4083,+ | Cga/Tga | R73* | PTC | AA->TER | No |
| 11 | 782659 | rs12806684 | BC019033 | HIT000038512 | 663,- | Gag/Tag | E161* | PTC | NA | Yes |
| 11 | 978657 | rs11538724 | AB020706 | HIT000000616 | 1237,+ | Gag/Tag | E413* | PTC | AA->TER | Yes |
| 11 | 2746687 | rs17215500 | AF000571 | HIT000061586 | 1662,+ | Cga/Tga | R518* | PTC | AA->TER | Yes |
| 11 | 3392787 | rs7118203 | ENST00000328202 | HIT000125419 | 677,- | Cga/Tga | R143* | PTC | AA->TER | No |
| 11 | 5400712 | rs2647574 | NM_001004757 | HIT000316013 | 706,- | Cga/Tga | R236* | PTC | AA->TER | No |
| 11 | 5419278 | rs16930998 | NM_001005288 | HIT000316112 | 43,- | Cag/Tag | Q15* | PTC | AA->TER | No |
| 11 | 5733060 | rs4910844 | NM_001005175 | HIT000316034 | 514,+ | Aga/Tga | R172* | PTC | AA->TER | No |
| 11 | 5834555 | rs12419602 | NM_001005168 | HIT000316027 | 954,- | taA/taT | *318Y | Read-through | AA->TER | NA |
| 11 | 6409098 | rs1042547 | CR611907 | HIT000291102 | 1319,+ | taC/taA | Y436* | PTC | AA->TER | No |
| 11 | 6870116 | rs16919417 | NM_003700 | HIT000316974 | 192,- | tgC/tgA | C64* | PTC | NA | No |
| 11 | 7669047 | rs4509745 | NM_198185 | HIT000319195 | 1668,- | tgG/tgA | W556* | PTC | AA->TER | No |
| 11 | 9757226 | rs360125 | CR611690 | HIT000290885 | 274,- | Taa/Gaa | *64E | Read-through | AA->TER | NA |

| Chr | Position | SNP ID | Accession no. | H-Inv ID | cDNA Pos. | Codon change | AA change | Effect | Inferred direction | NMD |
| --- | --- | --- | --- | --- | --- | --- | --- | --- | --- | --- |
| 11 | 11330729 | rs1056934 | AY112721 | HIT000084452 | 752,+ | Cga/Tga | R172* | PTC | NA | No |
| 11 | 12355793 | rs12791954 | AF237771 | HIT000074751 | 115,+ | tCg/tAg | S8* | PTC | NA | Yes |
| 11 | 15052227 | rs3812726 | AK127720 | HIT000047593 | 540,- | Cag/Tag | Q127* | PTC | AA->TER | No |
| 11 | 16722755 | rs11549934 | BC007103 | HIT000033051 | 227,+ | tTg/tAg | L32* | PTC | AA->TER | Yes |
| 11 | 18240837 | rs2896524 | AF512499 | HIT000081080 | 806,- | Cga/Tga | R245* | PTC | NA | No |
| 11 | 19130378 | rs11025035 | BC056152 | HIT000054134 | 787,+ | Gga/Tga | G228* | PTC | AA->TER | Yes |
| 11 | 26539189 | rs17851845 | BC020912 | HIT000090299 | 1350,- | tGg/tAg | W362* | PTC | TER->AA | No |
| 11 | 31864040 | rs623312 | BX648962 | HIT000057609 | 533,- | tgG/tgA | W3* | PTC | TER->AA | No |
| 11 | 34334855 | rs12792007 | AK095632 | HIT000020487 | 524,- | taT/taA | Y98* | PTC | AA->TER | Yes |
| 11 | 46353142 | rs11557399 | U94905 | HIT000222744 | 2370,+ | Gag/Tag | E749* | PTC | AA->TER | Yes |
| 11 | 46407120 | rs3961051 | AK025353 | HIT000008627 | 2785,- | Aag/Tag | K53* | PTC | AA->TER | No |
| 11 | 46407129 | rs11539340 | AK025353 | HIT000008627 | 2776,+ | Cag/Tag | Q50* | PTC | AA->TER | No |
| 11 | 47329450 | rs11570045 | X84075 | HIT000324022 | 240,+ | Gaa/Taa | E70* | PTC | AA->TER | Yes |
| 11 | 48223312 | rs7120775 | NM_001004727 | HIT000315983 | 81,+ | taC/taG | Y27* | PTC | AA->TER | No |
| 11 | 48242807 | rs10838851 | NM_001004726 | HIT000315982 | 819,+ | taT/taA | Y273* | PTC | AA->TER | No |
| 11 | 48879272 | rs10742909 | ENST00000309333 | HIT000118310 | 559,- | Cga/Tga | R187* | PTC | AA->TER | No |
| 11 | 48957134 | rs11040198 | ENST00000327733 | HIT000125160 | 604,- | Caa/Taa | Q202* | PTC | AA->TER | Yes |
| 11 | 49871603 | rs4881654 | ENST00000329376 | HIT000126087 | 303,- | tgG/tgA | W101* | PTC | NA | Yes |
| 11 | 51268106 | rs5029499 | NM_001005272 | HIT000316096 | 866,- | tCa/tAa | S289* | PTC | AA->TER | No |

| Chr | Position | SNP ID | Accession no. | H-Inv ID | cDNA Pos. | Codon change | AA change | Effect | Inferred direction | NMD |
| --- | --- | --- | --- | --- | --- | --- | --- | --- | --- | --- |
| 11 | 55096228 | rs1459101 | NM_001004701 | HIT000315962 | 49,- | Cag/Tag | Q17* | PTC | AA->TER | No |
| 11 | 55297590 | rs11230980 | NM_001001967 | HIT000315576 | 101,+ | tTg/tAg | L34* | PTC | AA->TER | No |
| 11 | 56187792 | rs11228710 | NM_001004730 | HIT000315986 | 55,+ | Cag/Tag | Q19* | PTC | AA->TER | No |
| 11 | 56512974 | rs13343184 | NM_001005323 | HIT000316117 | 10,+ | Gga/Tga | G4* | PTC | AA->TER | No |
| 11 | 56759081 | rs12270028 | CR605634 | HIT000284829 | 1389,+ | tgC/tgA | C51* | PTC | AA->TER | No |
| 11 | 57135216 | rs2454660 | AF086318 | HIT000067197 | 414,- | Tga/Cga | *49R | Read-through | TER->AA | NA |
| 11 | 57739770 | rs7103033 | NM_001004458 | HIT000315911 | 978,+ | tgA/tgG | *326W | Read-through | TER->AA | NA |
| 11 | 58733948 | rs12791481 | AK024358 | HIT000007632 | 1143,+ | Gaa/Taa | E83* | PTC | AA->TER | No |
| 11 | 59237528 | rs499037 | NM_001005324 | HIT000316118 | 367,- | Cag/Tag | Q123* | PTC | AA->TER | No |
| 11 | 59377056 | rs17851654 | J05068 | HIT000191310 | 1345,- | Gaa/Taa | E424* | PTC | AA->TER | No |
| 11 | 59916780 | rs11123 | AK075106 | HIT000082141 | 790,- | tTa/tAa | L198* | PTC | AA->TER | Yes |
| 11 | 60021578 | rs2298553 | AK000224 | HIT000002699 | 268,+ | Caa/Taa | Q71* | PTC | AA->TER | Yes |
| 11 | 60853611 | rs3018572 | BC050530 | HIT000053478 | 486,+ | Gag/Tag | E117* | PTC | AA->TER | Yes |
| 11 | 62083785 | rs11545775 | AK130026 | HIT000049297 | 1277,- | tGg/tAg | W419* | PTC | AA->TER | No |
| 11 | 62310853 | rs17850388 | AF069735 | HIT000065672 | 1378,+ | Cag/Tag | Q460* | PTC | AA->TER | No |
| 11 | 62523015 | rs11568496 | BC022387 | HIT000039514 | 851,+ | Cag/Tag | Q239* | PTC | AA->TER | Yes |
| 11 | 64132883 | rs3802947 | BC043422 | HIT000097672 | 1027,+ | tGg/tAg | W148* | PTC | AA->TER | No |
| 11 | 65469783 | rs9795469 | AK057442 | HIT000014056 | 93,+ | Cga/Tga | R21* | PTC | AA->TER | Yes |
| 11 | 66084671 | rs2228325 | M86407 | HIT000196626 | 1747,+ | Cga/Tga | R577* | PTC | AA->TER | Yes |

| Chr | Position | SNP ID | Accession no. | H-Inv ID | cDNA Pos. | Codon change | AA change | Effect | Inferred direction | NMD |
| --- | --- | --- | --- | --- | --- | --- | --- | --- | --- | --- |
| 11 | 66192131 | rs11227567 | AK094046 | HIT000018903 | 2048,+ | Tga/Gga | *60G | Read-through | TER->AA | NA |
| 11 | 66807799 | rs1573538 | X61157 | HIT000322454 | 1481,- | tgG/tgA | W458* | PTC | AA->TER | Yes |
| 11 | 67168890 | rs2514034 | BC008689 | HIT000033902 | 832,- | Gaa/Taa | E221* | PTC | AA->TER | Yes |
| 11 | 74889549 | rs1790144 | AL137552 | HIT000025840 | 1527,- | tGg/tAg | W45* | PTC | AA->TER | No |
| 11 | 78046806 | rs1061858 | BX640737 | HIT000055286 | 4145,+ | tCa/tAa | S1382* | PTC | AA->TER | No |
| 11 | 82581470 | rs2231729 | AL359570 | HIT000026298 | 421,+ | tgA/tgG | *107W | Read-through | TER->AA | NA |
| 11 | 82637407 | rs11233538 | AL137429 | HIT000025717 | 500,- | taA/taT | *55Y | Read-through | AA->TER | NA |
| 11 | 82641394 | rs11233539 | CR595419 | HIT000274614 | 864,- | tTa/tAa | L7* | PTC | AA->TER | No |
| 11 | 82648440 | rs492710 | CR604405 | HIT000283600 | 452,- | Gaa/Taa | E10* | PTC | AA->TER | No |
| 11 | 89655807 | rs11546585 | ENST00000318973 | HIT000121883 | 334,+ | Gag/Tag | E112* | PTC | AA->TER | Yes |
| 11 | 99195586 | rs12292659 | AB013802 | HIT000058342 | 236,+ | Cga/Tga | R53* | PTC | AA->TER | Yes |
| 11 | 104268327 | rs497116 | AY358222 | HIT000252266 | 404,+ | Cga/Tga | R125* | PTC | AA->TER | Yes |
| 11 | 111287571 | rs11549440 | BC007008 | HIT000032981 | 134,+ | Gag/Tag | E30* | PTC | AA->TER | Yes |
| 11 | 116665644 | rs1061659 | AF201468 | HIT000073772 | 1808,+ | Gag/Tag | E452* | PTC | AA->TER | No |
| 11 | 117728392 | rs1792836 | X04145 | HIT000321016 | 584,+ | Tga/Gga | *183G | Read-through | TER->AA | NA |
| 11 | 118421516 | rs555551 | U65785 | HIT000220888 | 3101,- | tAa/tTa | *1000L | Read-through | AA->TER | NA |
| 11 | 118990291 | rs871800 | AK095401 | HIT000020256 | 1083,+ | taC/taG | Y104* | PTC | TER->AA | No |
| 11 | 123626409 | rs4268525 | X89672 | HIT000324371 | 403,+ | taC/taG | Y134* | PTC | AA->TER | No |
| 11 | 124183891 | rs660396 | BC002761 | HIT000086161 | 1031,+ | Cag/Tag | Q10* | PTC | AA->TER | No |

| Chr | Position | SNP ID | Accession no. | H-Inv ID | cDNA Pos. | Codon change | AA change | Effect | Inferred direction | NMD |
| --- | --- | --- | --- | --- | --- | --- | --- | --- | --- | --- |
| 11 | 125639355 | rs17850298 | BC013583 | HIT000036289 | 1813,- | Cag/Tag | Q577* | PTC | AA->TER | Yes |
| 11 | 125668792 | rs17853052 | AF410783 | HIT000079214 | 1095,+ | tgA/tgG | *222W | Read-through | TER->AA | NA |
| 11 | 134170635 | rs4996609 | ENST00000360397 | HIT000313789 | 277,- | taC/taG | Y50* | PTC | NA | Yes |
| 11 | 134170700 | rs4996611 | ENST00000360397 | HIT000313789 | 212,- | Cag/Tag | Q29* | PTC | NA | Yes |
| 11 | 134361802 | rs28445859 | AK130852 | HIT000050123 | 1347,+ | taT/taG | Y400* | PTC | AA->TER | No |
| 12 | 892830 | rs4987208 | L33262 | HIT000193092 | 1514,+ | taT/taG | Y415* | PTC | AA->TER | No |
| 12 | 893479 | rs4987207 | L33262 | HIT000193092 | 1306,+ | tCg/tAg | S346* | PTC | AA->TER | Yes |
| 12 | 1767545 | rs2286380 | CR613880 | HIT000293075 | 551,+ | tgT/tgA | C56* | PTC | AA->TER | No |
| 12 | 3591206 | rs10848892 | AY358109 | HIT000252154 | 225,- | tTg/tAg | L71* | PTC | AA->TER | No |
| 12 | 4682772 | rs4766275 | BC043558 | HIT000097751 | 454,- | Gag/Tag | E2* | PTC | AA->TER | No |
| 12 | 5965048 | rs5020485 | X04385 | HIT000321031 | 6942,- | tgC/tgA | C2281* | PTC | AA->TER | Yes |
| 12 | 6501242 | rs3180469 | BC028182 | HIT000040480 | 1790,+ | Caa/Taa | Q578* | PTC | AA->TER | Yes |
| 12 | 6517313 | rs1062442 | CR600594 | HIT000279789 | 976,+ | taC/taA | Y276* | PTC | AA->TER | Yes |
| 12 | 7366348 | rs7485773 | ENST00000340801 | HIT000133431 | 1069,+ | Cag/Tag | Q357* | PTC | AA->TER | Yes |
| 12 | 9412026 | rs4883251 | BC047234 | HIT000098489 | 700,+ | Tga/Gga | *71G | Read-through | TER->AA | NA |
| 12 | 9705443 | rs7304321 | BC040884 | HIT000096422 | 2002,+ | tgT/tgA | C42* | PTC | AA->TER | No |
| 12 | 10162354 | rs16910526 | AF313468 | HIT000077083 | 765,- | taT/taG | Y238* | PTC | AA->TER | Yes |
| 12 | 11074302 | rs3759247 | NM_176885 | HIT000215440 | 900,+ | tgG/tgA | W300* | PTC | AA->TER | No |
| 12 | 11322890 | rs7955473 | AF086199 | HIT000067078 | 283,- | tgA/tgG | *56W | Read-through | TER->AA | NA |

| Chr | Position | SNP ID | Accession no. | H-Inv ID | cDNA Pos. | Codon change | AA change | Effect | Inferred direction | NMD |
| --- | --- | --- | --- | --- | --- | --- | --- | --- | --- | --- |
| 12 | 11353069 | rs12829245 | K03207 | HIT000191423 | 151,- | Cga/Tga | R39* | PTC | AA->TER | Yes |
| 12 | 13027890 | rs735689 | AK093401 | HIT000018268 | 794,- | Caa/Taa | Q11* | PTC | TER->AA | No |
| 12 | 14926414 | rs12304 | BC005272 | HIT000032339 | 301,+ | Gaa/Taa | E80* | PTC | AA->TER | No |
| 12 | 16408058 | rs9332944 | CR618561 | HIT000297756 | 371,+ | tTg/tAg | L95* | PTC | AA->TER | No |
| 12 | 18783238 | rs15547 | AB053259 | HIT000059669 | 902,- | Cga/Tga | R257* | PTC | AA->TER | No |
| 12 | 19318841 | rs12369271 | AB051473 | HIT000001402 | 956,+ | Gaa/Taa | E318* | PTC | AA->TER | Yes |
| 12 | 23672817 | rs9300160 | AK130118 | HIT000049389 | 530,- | Cga/Tga | R109* | PTC | TER->AA | No |
| 12 | 25038459 | rs16928315 | BC035880 | HIT000051541 | 693,- | Aga/Tga | R34* | PTC | TER->AA | No |
| 12 | 31157192 | rs17403742 | BC039117 | HIT000095526 | 1195,+ | taG/taC | *87Y | Read-through | NA | NA |
| 12 | 31741962 | rs12823062 | CR598985 | HIT000278180 | 915,- | tgT/tgA | C153* | PTC | AA->TER | Yes |
| 12 | 32562594 | rs7308139 | AF087961 | HIT000067509 | 435,- | Cga/Tga | R53* | PTC | AA->TER | No |
| 12 | 39002106 | rs11564176 | AY792511 | HIT000257811 | 5173,+ | Cga/Tga | R1725* | PTC | AA->TER | Yes |
| 12 | 39121222 | rs17492404 | ENST00000340554 | HIT000133184 | 81,+ | tgC/tgA | C27* | PTC | AA->TER | Yes |
| 12 | 39159545 | rs11176811 | ENST00000328802 | HIT000125762 | 605,+ | tTa/tAa | L100* | PTC | AA->TER | Yes |
| 12 | 39160211 | rs11176815 | ENST00000328802 | HIT000125762 | 1271,+ | tCa/tGa | S322* | PTC | AA->TER | Yes |
| 12 | 39167943 | rs2452315 | ENST00000328802 | HIT000125762 | 8945,+ | tCa/tAa | S2880* | PTC | NA | No |
| 12 | 41140138 | rs3827522 | BC042722 | HIT000097327 | 705,+ | tgG/tgA | W94* | PTC | AA->TER | Yes |
| 12 | 44865655 | rs3497 | AK025900 | HIT000009174 | 854,- | Cag/Tag | Q71* | PTC | AA->TER | No |
| 12 | 47023148 | rs17851617 | AY842285 | HIT000257886 | 1908,- | Aag/Tag | K398* | PTC | AA->TER | No |

| Chr | Position | SNP ID | Accession no. | H-Inv ID | cDNA Pos. | Codon change | AA change | Effect | Inferred direction | NMD |
| --- | --- | --- | --- | --- | --- | --- | --- | --- | --- | --- |
| 12 | 47607785 | rs941193 | AK123484 | HIT000043357 | 1195,- | Tag/Cag | *114Q | Read-through | AA->TER | NA |
| 12 | 50913627 | rs11558308 | BC002700 | HIT000031109 | 322,+ | Cag/Tag | Q94* | PTC | AA->TER | Yes |
| 12 | 51113180 | rs2232389 | Y17282 | HIT000325996 | 640,+ | Cga/Tga | R208* | PTC | AA->TER | Yes |
| 12 | 51151489 | rs430612 | L42611 | HIT000193818 | 935,+ | Aga/Tga | R299* | PTC | AA->TER | Yes |
| 12 | 51411630 | rs12830331 | ENST00000321887 | HIT000122930 | 664,+ | Cga/Tga | R222* | PTC | AA->TER | No |
| 12 | 51579960 | rs11554487 | CR605272 | HIT000284467 | 938,+ | Cag/Tag | Q312* | PTC | AA->TER | Yes |
| 12 | 52863985 | rs2233919 | BC000417 | HIT000029603 | 377,+ | Cag/Tag | Q3* | PTC | AA->TER | Yes |
| 12 | 53927522 | rs4522268 | NM_001005490 | HIT000316192 | 184,+ | Cga/Tga | R62* | PTC | AA->TER | No |
| 12 | 54522869 | rs17844787 | U37791 | HIT000219537 | 94,+ | Cag/Tag | Q5* | PTC | AA->TER | Yes |
| 12 | 54641748 | rs11552309 | AK092881 | HIT000017748 | 776,+ | Caa/Taa | Q38* | PTC | AA->TER | Yes |
| 12 | 55271825 | rs2950389 | CR597503 | HIT000276698 | 156,- | taG/taT | *27Y | Read-through | AA->TER | NA |
| 12 | 55911048 | rs12319666 | BC011911 | HIT000035537 | 239,+ | Gag/Tag | E77* | PTC | AA->TER | Yes |
| 12 | 61294855 | rs12581368 | AK024262 | HIT000007536 | 2105,- | Gag/Tag | E16* | PTC | AA->TER | No |
| 12 | 61645756 | rs11539059 | CR617698 | HIT000296893 | 390,+ | Aag/Tag | K129* | PTC | AA->TER | No |
| 12 | 62827575 | rs11610419 | BC006127 | HIT000086594 | 417,+ | tgT/tgA | C21* | PTC | AA->TER | No |
| 12 | 65651757 | rs3942826 | CR593338 | HIT000272533 | 914,- | taT/taG | Y6* | PTC | AA->TER | No |
| 12 | 67233765 | rs2920025 | ENST00000361461 | HIT000314724 | 165,- | tgG/tgA | W55* | PTC | AA->TER | No |
| 12 | 67322563 | rs12817631 | ENST00000361329 | HIT000314595 | 439,- | Taa/Aaa | *147K | Read-through | AA->TER | NA |
| 12 | 67322581 | rs12818158 | ENST00000361329 | HIT000314595 | 421,- | Caa/Taa | Q141* | PTC | AA->TER | No |

| Chr | Position | SNP ID | Accession no. | H-Inv ID | cDNA Pos. | Codon change | AA change | Effect | Inferred direction | NMD |
| --- | --- | --- | --- | --- | --- | --- | --- | --- | --- | --- |
| 12 | 67355001 | rs11177329 | CR620484 | HIT000299679 | 1094,- | tCa/tGa | S8* | PTC | AA->TER | No |
| 12 | 68255001 | rs17851899 | BC021562 | HIT000039218 | 1772,+ | tTa/tGa | L509* | PTC | TER->AA | No |
| 12 | 68277268 | rs11541105 | AF026166 | HIT000062801 | 1105,+ | Gag/Tag | E350* | PTC | AA->TER | Yes |
| 12 | 74727867 | rs9836 | AF088017 | HIT000067565 | 124,+ | tgG/tgA | W25* | PTC | AA->TER | No |
| 12 | 78141942 | rs1245816 | BX538227 | HIT000055009 | 5541,+ | Caa/Taa | Q59* | PTC | AA->TER | No |
| 12 | 78192440 | rs7972950 | AK094616 | HIT000019471 | 1269,+ | Cga/Tga | R200* | PTC | AA->TER | Yes |
| 12 | 81947183 | rs421560 | AL834509 | HIT000029239 | 2617,+ | Gaa/Taa | E729* | PTC | AA->TER | Yes |
| 12 | 92400717 | rs7971966 | AK094874 | HIT000019729 | 464,+ | tGg/tAg | W123* | PTC | AA->TER | Yes |
| 12 | 93466280 | rs11107605 | CR620972 | HIT000300167 | 498,+ | Tga/Gga | *70G | Read-through | TER->AA | NA |
| 12 | 94775978 | rs11108315 | AK097408 | HIT000022262 | 1288,- | Cag/Tag | Q352* | PTC | AA->TER | Yes |
| 12 | 98991953 | rs12824013 | AB014601 | HIT000000426 | 470,- | tCa/tAa | S157* | PTC | AA->TER | Yes |
| 12 | 99239072 | rs11110344 | AK021434 | HIT000004708 | 943,+ | tgG/tgA | W26* | PTC | AA->TER | No |
| 12 | 100583948 | rs10128995 | AK024525 | HIT000007799 | 89,+ | tgG/tgA | W17* | PTC | AA->TER | No |
| 12 | 109369503 | rs12230101 | CR623239 | HIT000302434 | 522,+ | Gag/Tag | E35* | PTC | AA->TER | Yes |
| 12 | 110545037 | rs11541241 | AY332222 | HIT000251874 | 1604,+ | Gag/Tag | E489* | PTC | AA->TER | No |
| 12 | 110911816 | rs12298539 | AK021528 | HIT000004802 | 867,+ | Tag/Cag | *159Q | Read-through | TER->AA | NA |
| 12 | 111307406 | rs17856609 | BC004138 | HIT000031725 | 376,- | taT/taG | Y115* | PTC | AA->TER | Yes |
| 12 | 111308974 | rs17856608 | BC004138 | HIT000031725 | 238,- | taC/taG | Y69* | PTC | AA->TER | Yes |
| 12 | 111911008 | rs15895 | M87434 | HIT000196666 | 2178,- | tGg/tAg | W720* | PTC | TER->AA | No |

| Chr | Position | SNP ID | Accession no. | H-Inv ID | cDNA Pos. | Codon change | AA change | Effect | Inferred direction | NMD |
| --- | --- | --- | --- | --- | --- | --- | --- | --- | --- | --- |
| 12 | 115107606 | rs11611787 | AK056901 | HIT000013515 | 1596,- | tCa/tAa | S41* | PTC | AA->TER | No |
| 12 | 116043222 | rs12824193 | U79257 | HIT000221481 | 779,- | tgT/tgA | C13* | PTC | AA->TER | No |
| 12 | 118572832 | rs17400082 | BC017671 | HIT000038073 | 441,+ | Gag/Tag | E56* | PTC | AA->TER | Yes |
| 12 | 119097346 | rs11548375 | AK129823 | HIT000049094 | 980,+ | Gaa/Taa | E302* | PTC | AA->TER | No |
| 12 | 119345396 | rs11558727 | BC002638 | HIT000031056 | 257,+ | tGa/tCa | *77S | Read-through | TER->AA | NA |
| 12 | 119477124 | rs10056 | BC016622 | HIT000037603 | 2818,+ | Gga/Tga | G791* | PTC | AA->TER | No |
| 12 | 121725893 | rs3825147 | BC047891 | HIT000098820 | 332,- | tCa/tGa | S91* | PTC | NA | No |
| 12 | 122161968 | rs5007968 | X98258 | HIT000325058 | 639,+ | Caa/Taa | Q213* | PTC | AA->TER | No |
| 12 | 122418352 | rs11833601 | CR605053 | HIT000284248 | 425,- | Aag/Tag | K36* | PTC | NA | No |
| 12 | 127803084 | rs7965732 | CR609573 | HIT000288768 | 442,+ | Aga/Tga | R50* | PTC | AA->TER | No |
| 12 | 129884035 | rs11546493 | CR605944 | HIT000285139 | 383,+ | tgG/tgA | W104* | PTC | AA->TER | Yes |
| 12 | 131296367 | rs11543306 | BC001191 | HIT000030211 | 313,+ | tGg/tAg | W91* | PTC | AA->TER | Yes |
| 12 | 131752323 | rs5023077 | BC068083 | HIT000262942 | 317,- | Cga/Tga | R2* | PTC | NA | No |
| 12 | 131821209 | rs7487595 | L09561 | HIT000191980 | 5961,- | Gag/Tag | E1973* | PTC | AA->TER | Yes |
| 12 | 131866568 | rs12315832 | L09561 | HIT000191980 | 487,- | tTg/tAg | L148* | PTC | AA->TER | Yes |
| 12 | 132249179 | rs659137 | AK055603 | HIT000012217 | 1056,+ | tgG/tgA | W60* | PTC | AA->TER | No |
| 13 | 18138433 | rs9579915 | ENST00000331097 | HIT000127037 | 778,+ | Gag/Tag | E260* | PTC | AA->TER | No |
| 13 | 20113523 | rs4145579 | U20362 | HIT000218556 | 1859,+ | taC/taG | Y556* | PTC | AA->TER | Yes |
| 13 | 24384734 | rs28366805 | AF139625 | HIT000071125 | 640,- | Aaa/Taa | K144* | PTC | AA->TER | Yes |

| Chr | Position | SNP ID | Accession no. | H-Inv ID | cDNA Pos. | Codon change | AA change | Effect | Inferred direction | NMD |
| --- | --- | --- | --- | --- | --- | --- | --- | --- | --- | --- |
| 13 | 24426915 | rs28538644 | AK130982 | HIT000050253 | 281,+ | Tga/Cga | *59R | Read-through | ter2aa | NA |
| 13 | 29935420 | rs11546137 | AK122825 | HIT000042698 | 1163,+ | Gaa/Taa | E74* | PTC | AA->TER | Yes |
| 13 | 31870626 | rs11571833 | U43746 | HIT000219873 | 10204,+ | Aaa/Taa | K3326* | PTC | AA->TER | Yes |
| 13 | 36478125 | rs11550130 | BC020773 | HIT000038951 | 333,+ | Caa/Taa | Q103* | PTC | AA->TER | Yes |
| 13 | 41599674 | rs9566925 | AB078968 | HIT000061346 | 289,+ | Cga/Tga | R90* | PTC | AA->TER | Yes |
| 13 | 42256264 | rs1853636 | AK098238 | HIT000023068 | 125,+ | Cga/Tga | R21* | PTC | AA->TER | Yes |
| 13 | 44048205 | rs2297736 | BC040675 | HIT000096324 | 177,- | tgG/tgA | W15* | PTC | AA->TER | Yes |
| 13 | 44461859 | rs9567515 | AB051491 | HIT000001420 | 105,+ | Gag/Tag | E24* | PTC | AA->TER | Yes |
| 13 | 44812285 | rs11552490 | CR620037 | HIT000299232 | 232,+ | tCg/tAg | S46* | PTC | AA->TER | Yes |
| 13 | 45482562 | rs1055768 | AY283618 | HIT000085749 | 739,+ | tCa/tAa | S223* | PTC | AA->TER | Yes |
| 13 | 47851731 | rs3092891 | L41870 | HIT000193791 | 1470,+ | Cga/Tga | R445* | PTC | AA->TER | Yes |
| 13 | 53588106 | rs1056114 | BC026300 | HIT000091258 | 872,+ | Cga/Tga | R24* | PTC | AA->TER | No |
| 13 | 60203627 | rs7320416 | BC041832 | HIT000096880 | 1317,+ | Aga/Tga | R46* | PTC | AA->TER | No |
| 13 | 63309936 | rs7329005 | AL137719 | HIT000026007 | 229,- | Aga/Tga | R54* | PTC | NA | No |
| 13 | 63309996 | rs2635879 | AL137719 | HIT000026007 | 170,+ | tCg/tAg | S34* | PTC | AA->TER | No |
| 13 | 74711256 | rs12854011 | AL133018 | HIT000024922 | 1263,- | Gaa/Taa | E139* | PTC | AA->TER | No |
| 13 | 74711267 | rs12854029 | AL133018 | HIT000024922 | 1252,- | tGg/tAg | W135* | PTC | AA->TER | No |
| 13 | 74711349 | rs475484 | AL133018 | HIT000024922 | 1170,- | Cag/Tag | Q108* | PTC | AA->TER | No |
| 13 | 75002353 | rs1063482 | AK000009 | HIT000002484 | 141,+ | tgC/tgA | C35* | PTC | AA->TER | Yes |

| Chr | Position | SNP ID | Accession no. | H-Inv ID | cDNA Pos. | Codon change | AA change | Effect | Inferred direction | NMD |
| --- | --- | --- | --- | --- | --- | --- | --- | --- | --- | --- |
| 13 | 78785412 | rs1748768 | BC043240 | HIT000097561 | 847,+ | Aaa/Taa | K59* | PTC | NA | No |
| 13 | 97866256 | rs12429460 | AK025341 | HIT000008615 | 362,+ | Cag/Tag | Q53* | PTC | TER->AA | No |
| 13 | 97955530 | rs3742133 | AK026896 | HIT000010170 | 728,+ | Cag/Tag | Q40* | PTC | AA->TER | Yes |
| 13 | 101163163 | rs2281988 | AK095102 | HIT000019957 | 2009,+ | tCg/tAg | S12* | PTC | TER->AA | No |
| 13 | 101845956 | rs3168724 | AF086059 | HIT000066938 | 108,- | Gaa/Taa | E14* | PTC | TER->AA | No |
| 13 | 107661480 | rs2232636 | BC037491 | HIT000051922 | 290,+ | tgG/tgA | W46* | PTC | AA->TER | No |
| 13 | 109907284 | rs9588168 | AK130129 | HIT000049400 | 1023,- | tCa/tGa | S277* | PTC | AA->TER | No |
| 13 | 114018671 | rs9590491 | BC017244 | HIT000037899 | 118,+ | Gag/Tag | E4* | PTC | AA->TER | Yes |
| 14 | 19768079 | rs1953558 | ENST00000315508 | HIT000120537 | 679,+ | Tag/Cag | *227Q | Read-through | AA->TER | NA |
| 14 | 21060021 | rs741707 | AK001276 | HIT000003750 | 602,+ | Caa/Taa | Q197* | PTC | AA->TER | No |
| 14 | 23835834 | rs2295305 | AF418205 | HIT000079346 | 457,+ | Cag/Tag | Q82* | PTC | AA->TER | Yes |
| 14 | 24046497 | rs13306254 | BC069110 | HIT000263282 | 144,+ | taC/taA | Y38* | PTC | AA->TER | Yes |
| 14 | 24173254 | rs2273844 | AY372494 | HIT000253319 | 60,- | Caa/Taa | Q19* | PTC | AA->TER | Yes |
| 14 | 29153832 | rs12878607 | BC062469 | HIT000260432 | 969,- | Aaa/Taa | K71* | PTC | NA | No |
| 14 | 38720816 | rs11558737 | Y09703 | HIT000325539 | 2165,+ | Taa/Caa | *587Q | Read-through | TER->AA | NA |
| 14 | 49122791 | rs11548399 | AK026649 | HIT000009923 | 54,+ | tgG/tgA | W8* | PTC | AA->TER | Yes |
| 14 | 49186903 | rs3218790 | AF036899 | HIT000063345 | 1341,+ | Aag/Tag | K443* | PTC | AA->TER | Yes |
| 14 | 49872477 | rs7148089 | AF390028 | HIT000078821 | 2966,- | tTa/tGa | L989* | PTC | AA->TER | No |
| 14 | 49879445 | rs11570829 | AF390028 | HIT000078821 | 2308,+ | Cga/Tga | R770* | PTC | AA->TER | Yes |

| Chr | Position | SNP ID | Accession no. | H-Inv ID | cDNA Pos. | Codon change | AA change | Effect | Inferred direction | NMD |
| --- | --- | --- | --- | --- | --- | --- | --- | --- | --- | --- |
| 14 | 54681647 | rs11001 | M57710 | HIT000195836 | 676,+ | Cag/Tag | Q220* | PTC | AA->TER | No |
| 14 | 54835595 | rs28379450 | AL834124 | HIT000028855 | 1928,+ | Cag/Tag | Q167* | PTC | AA->TER | No |
| 14 | 59009518 | rs1046700 | AK058165 | HIT000014771 | 1022,+ | tCa/tAa | S328* | PTC | AA->TER | No |
| 14 | 59015670 | rs17415478 | AK058165 | HIT000014771 | 700,+ | Gag/Tag | E221* | PTC | NA | Yes |
| 14 | 59662154 | rs11625823 | AK095489 | HIT000020344 | 2931,+ | Gaa/Taa | E809* | PTC | AA->TER | Yes |
| 14 | 63629845 | rs2781377 | AF435011 | HIT000079629 | 12214,- | tGg/tAg | W4001* | PTC | AA->TER | Yes |
| 14 | 67152128 | rs12100644 | BC068102 | HIT000262961 | 734,+ | Caa/Taa | Q61* | PTC | AA->TER | No |
| 14 | 68329415 | rs1051533 | X79066 | HIT000323700 | 330,- | tgC/tgA | C12* | PTC | AA->TER | No |
| 14 | 70005689 | rs17107991 | BC047939 | HIT000098848 | 2696,- | Gag/Tag | E314* | PTC | AA->TER | No |
| 14 | 72148971 | rs8011029 | AK024141 | HIT000007415 | 646,- | taT/taA | Y211* | PTC | TER->AA | No |
| 14 | 73497631 | rs3179800 | AK130167 | HIT000049438 | 932,- | tgG/tgA | W298* | PTC | AA->TER | Yes |
| 14 | 74228760 | rs2270424 | BC032944 | HIT000051094 | 201,- | Cga/Tga | R67* | PTC | AA->TER | Yes |
| 14 | 74364132 | rs1057538 | BX647164 | HIT000055811 | 2156,+ | Gga/Tga | G102* | PTC | AA->TER | No |
| 14 | 74997942 | rs3178799 | BC068196 | HIT000262967 | 535,+ | Cga/Tga | R88* | PTC | AA->TER | No |
| 14 | 80713553 | rs4899786 | BC041410 | HIT000096681 | 1730,- | tgC/tgA | C12* | PTC | AA->TER | No |
| 14 | 89943526 | rs11539544 | U16850 | HIT000218355 | 838,- | Gag/Tag | E59* | PTC | AA->TER | No |
| 14 | 90302796 | rs3208874 | ENST00000328174 | HIT000125405 | 127,+ | Gga/Tga | G43* | PTC | AA->TER | No |
| 14 | 91146606 | rs1743155 | AK126034 | HIT000045907 | 2710,+ | Cag/Tag | Q857* | PTC | AA->TER | Yes |
| 14 | 92477239 | rs12588550 | CR591450 | HIT000270645 | 1343,+ | Cga/Tga | R88* | PTC | NA | No |

| Chr | Position | SNP ID | Accession no. | H-Inv ID | cDNA Pos. | Codon change | AA change | Effect | Inferred direction | NMD |
| --- | --- | --- | --- | --- | --- | --- | --- | --- | --- | --- |
| 14 | 93826422 | rs2232698 | BC022261 | HIT000039436 | 395,+ | Cga/Tga | R88* | PTC | AA->TER | Yes |
| 14 | 94662802 | rs12432511 | AB023145 | HIT000000645 | 1009,- | tgT/tgA | C257* | PTC | AA->TER | Yes |
| 14 | 95918572 | rs12435565 | AK000796 | HIT000003271 | 380,+ | Gaa/Taa | E79* | PTC | AA->TER | No |
| 14 | 96019304 | rs17853409 | AK057426 | HIT000014040 | 2013,+ | Gag/Tag | E657* | PTC | AA->TER | Yes |
| 14 | 99620778 | rs1190975 | BC023997 | HIT000039713 | 249,+ | Gaa/Taa | E3* | PTC | AA->TER | Yes |
| 14 | 100384817 | rs9743942 | AK098378 | HIT000023208 | 893,- | tTa/tAa | L143* | PTC | AA->TER | No |
| 14 | 101519192 | rs7157623 | AB002323 | HIT000000031 | 890,+ | Gag/Tag | E285* | PTC | AA->TER | Yes |
| 14 | 102663343 | rs12884397 | M92357 | HIT000196821 | 615,+ | Gag/Tag | E162* | PTC | NA | Yes |
| 14 | 103236778 | rs3212101 | L04733 | HIT000191645 | 1948,- | Cga/Tga | R562* | PTC | AA->TER | No |
| 14 | 104317518 | rs11555436 | BC084538 | HIT000266047 | 545,+ | Cag/Tag | Q43* | PTC | AA->TER | Yes |
| 14 | 104527306 | rs2819465 | AK022356 | HIT000005630 | 1036,- | Cag/Tag | Q115* | PTC | TER->AA | No |
| 14 | 105462118 | rs3094083 | AK125627 | HIT000045500 | 2184,- | Gaa/Taa | E58* | PTC | NA | No |
| 15 | 19195065 | rs4984119 | AK093817 | HIT000018684 | 1619,+ | Cga/Tga | R33* | PTC | NA | No |
| 15 | 19941661 | rs2059928 | CR590938 | HIT000270133 | 557,- | Tga/Cga | *184R | Read-through | NA | NA |
| 15 | 23374977 | rs2628635 | BC041438 | HIT000096706 | 55,+ | tgG/tgA | W16* | PTC | AA->TER | Yes |
| 15 | 26789875 | rs410966 | BC035099 | HIT000093688 | 571,- | tgT/tgA | C18* | PTC | AA->TER | Yes |
| 15 | 28124450 | rs11557903 | ENST00000312470 | HIT000119395 | 377,+ | tCa/tAa | S126* | PTC | AA->TER | No |
| 15 | 29082006 | rs3784589 | AB115501 | HIT000242386 | 3913,- | Gaa/Taa | E1305* | PTC | AA->TER | No |
| 15 | 31390554 | rs12912656 | AJ001515 | HIT000243880 | 86,+ | Gaa/Taa | E6* | PTC | AA->TER | Yes |

| Chr | Position | SNP ID | Accession no. | H-Inv ID | cDNA Pos. | Codon change | AA change | Effect | Inferred direction | NMD |
| --- | --- | --- | --- | --- | --- | --- | --- | --- | --- | --- |
| 15 | 32606274 | rs16954481 | CR594874 | HIT000274069 | 1019,- | Cga/Tga | R46* | PTC | NA | No |
| 15 | 36355053 | rs7180580 | AK128458 | HIT000048331 | 476,+ | Cga/Tga | R109* | PTC | AA->TER | No |
| 15 | 38256165 | rs28989186 | AF068760 | HIT000065622 | 686,+ | Cga/Tga | R194* | PTC | AA->TER | Yes |
| 15 | 38325404 | rs6492938 | AK097601 | HIT000022455 | 1456,- | tCa/tGa | S132* | PTC | AA->TER | No |
| 15 | 38469033 | rs11541640 | AK125765 | HIT000045638 | 592,+ | Gag/Tag | E72* | PTC | AA->TER | Yes |
| 15 | 39584263 | rs2278658 | D16105 | HIT000100571 | 2217,- | tGg/tAg | W707* | PTC | AA->TER | Yes |
| 15 | 39951844 | rs8029609 | AF233523 | HIT000074689 | 5341,- | Cag/Tag | Q1705* | PTC | NA | Yes |
| 15 | 39972774 | rs2271286 | AF233523 | HIT000074689 | 442,- | Cag/Tag | Q72* | PTC | AA->TER | Yes |
| 15 | 41446227 | rs12912744 | BC043612 | HIT000097796 | 710,- | tgG/tgA | W187* | PTC | AA->TER | Yes |
| 15 | 41684210 | rs2614824 | AK124582 | HIT000044455 | 1848,- | Caa/Taa | Q45* | PTC | NA | Yes |
| 15 | 41824569 | rs2597079 | BC066967 | HIT000262387 | 343,- | Cga/Tga | R14* | PTC | AA->TER | Yes |
| 15 | 42813227 | rs1060392 | CR592231 | HIT000271426 | 236,- | tGg/tAg | W28* | PTC | AA->TER | No |
| 15 | 43481610 | rs3809472 | BC039389 | HIT000095657 | 203,- | taC/taA | Y2* | PTC | AA->TER | Yes |
| 15 | 43489865 | rs17857406 | BC051861 | HIT000053717 | 1333,+ | Tag/Cag | *427Q | Read-through | AA->TER | NA |
| 15 | 46524919 | rs363807 | L13923 | HIT000192231 | 5996,+ | Cag/Tag | Q1955* | PTC | AA->TER | Yes |
| 15 | 46524956 | rs363806 | L13923 | HIT000192231 | 5959,+ | tgC/tgA | C1942* | PTC | AA->TER | Yes |
| 15 | 46545069 | rs140630 | L13923 | HIT000192231 | 5063,+ | Cga/Tga | R1644* | PTC | AA->TER | Yes |
| 15 | 46574708 | rs140583 | L13923 | HIT000192231 | 2714,+ | Cga/Tga | R861* | PTC | AA->TER | Yes |
| 15 | 47873772 | rs10519239 | ENST00000332687 | HIT000127930 | 40,+ | Cag/Tag | Q14* | PTC | AA->TER | Yes |

| Chr | Position | SNP ID | Accession no. | H-Inv ID | cDNA Pos. | Codon change | AA change | Effect | Inferred direction | NMD |
| --- | --- | --- | --- | --- | --- | --- | --- | --- | --- | --- |
| 15 | 48434075 | rs8552 | AK000900 | HIT000003374 | 277,- | tgG/tgA | W37* | PTC | AA->TER | No |
| 15 | 48785436 | rs12905720 | AF075008 | HIT000066030 | 471,+ | taA/taT | *36Y | Read-through | TER->AA | NA |
| 15 | 53913532 | rs17415372 | D42055 | HIT000042247 | 2508,+ | tGg/tAg | W809* | PTC | NA | Yes |
| 15 | 59164612 | rs7181279 | AK021439 | HIT000004713 | 1256,- | tGg/tAg | W76* | PTC | AA->TER | No |
| 15 | 63825190 | rs12906813 | AF090925 | HIT000068257 | 1094,+ | taT/taG | Y10* | PTC | AA->TER | No |
| 15 | 64428763 | rs11538107 | BC000870 | HIT000029965 | 416,+ | Cag/Tag | Q119* | PTC | AA->TER | Yes |
| 15 | 64940607 | rs10162949 | BX538204 | HIT000054986 | 913,+ | Cag/Tag | Q46* | PTC | AA->TER | No |
| 15 | 66179201 | rs28754402 | BC036508 | HIT000094453 | 2108,+ | taT/taA | Y59* | PTC | AA->TER | No |
| 15 | 67833547 | rs7180567 | AK127674 | HIT000047547 | 32,- | Caa/Taa | Q9* | PTC | AA->TER | No |
| 15 | 69902284 | rs2246225 | BC039397 | HIT000095664 | 734,- | tgA/tgG | *54W | Read-through | AA->TER | NA |
| 15 | 70288149 | rs11558352 | BC000481 | HIT000029665 | 807,+ | Cag/Tag | Q235* | PTC | AA->TER | Yes |
| 15 | 72122464 | rs11272 | AF230401 | HIT000074570 | 1864,+ | Gag/Tag | E598* | PTC | AA->TER | Yes |
| 15 | 73008579 | rs11553377 | M22760 | HIT000194859 | 167,+ | Gag/Tag | E50* | PTC | AA->TER | Yes |
| 15 | 73803638 | rs28413581 | BC039862 | HIT000052275 | 265,+ | Cag/Tag | Q15* | PTC | AA->TER | Yes |
| 15 | 75027912 | rs11547509 | CR613141 | HIT000292336 | 829,+ | tgG/tgA | W255* | PTC | AA->TER | No |
| 15 | 78069868 | rs2586157 | AF161558 | HIT000002156 | 567,- | Cag/Tag | Q97* | PTC | AA->TER | No |
| 15 | 79388012 | rs17875553 | AY324389 | HIT000251760 | 3817,+ | Gaa/Taa | E1273* | PTC | AA->TER | No |
| 15 | 79785775 | rs367607 | AL109688 | HIT000250173 | 354,+ | Caa/Taa | Q88* | PTC | NA | No |
| 15 | 81117996 | rs12385960 | BC040988 | HIT000096503 | 2000,+ | Cga/Tga | R42* | PTC | AA->TER | No |

| Chr | Position | SNP ID | Accession no. | H-Inv ID | cDNA Pos. | Codon change | AA change | Effect | Inferred direction | NMD |
| --- | --- | --- | --- | --- | --- | --- | --- | --- | --- | --- |
| 15 | 82749329 | rs469020 | AB067507 | HIT000001635 | 6405,+ | Tag/Gag | *446E | Read-through | NA | NA |
| 15 | 82856301 | rs4039982 | AK092490 | HIT000017357 | 876,+ | Gag/Tag | E213* | PTC | NA | Yes |
| 15 | 82856321 | rs4039978 | AK092490 | HIT000017357 | 856,+ | tTa/tGa | L206* | PTC | NA | Yes |
| 15 | 87237299 | rs8039131 | BC062320 | HIT000260321 | 188,- | Cag/Tag | Q17* | PTC | AA->TER | Yes |
| 15 | 89280614 | rs11542714 | AK125721 | HIT000045594 | 1136,+ | Cag/Tag | Q101* | PTC | AA->TER | Yes |
| 15 | 90501413 | rs7181257 | BC043379 | HIT000097639 | 248,- | tGg/tAg | W48* | PTC | AA->TER | No |
| 15 | 91107162 | rs8030913 | ENST00000330136 | HIT000126497 | 295,+ | Gaa/Taa | E11* | PTC | AA->TER | Yes |
| 15 | 92712024 | rs2289010 | BC041387 | HIT000096661 | 1976,- | taT/taA | Y225* | PTC | AA->TER | Yes |
| 15 | 92728302 | rs28599939 | BC041387 | HIT000096661 | 2118,+ | Cag/Tag | Q273* | PTC | AA->TER | Yes |
| 15 | 94613739 | rs4997287 | AK000872 | HIT000003347 | 1183,- | Aaa/Taa | K68* | PTC | NA | No |
| 15 | 95102199 | rs7180888 | ENST00000344738 | HIT000137368 | 508,+ | Cga/Tga | R170* | PTC | AA->TER | Yes |
| 15 | 95128397 | rs3812907 | AY489187 | HIT000254914 | 313,+ | Cga/Tga | R34* | PTC | AA->TER | Yes |
| 16 | 711847 | rs12931094 | BC001181 | HIT000030202 | 430,+ | Gag/Tag | E105* | PTC | AA->TER | Yes |
| 16 | 1095026 | rs533184 | ENST00000293900 | HIT000113773 | 986,+ | tGg/tAg | W329* | PTC | TER->AA | Yes |
| 16 | 1424393 | rs12598718 | BC061641 | HIT000260224 | 460,- | taG/taC | *106Y | Read-through | TER->AA | NA |
| 16 | 1863936 | rs2815297 | BC036644 | HIT000094542 | 579,+ | Cga/Tga | R75* | PTC | AA->TER | No |
| 16 | 2096601 | rs2432403 | L33243 | HIT000193091 | 7499,+ | Cga/Tga | R2430* | PTC | NA | Yes |
| 16 | 2651369 | rs4786312 | BC043252 | HIT000097573 | 5360,- | Taa/Caa | *483Q | Read-through | NA | NA |
| 16 | 4978205 | rs12232463 | AB007880 | HIT000000137 | 448,+ | Gag/Tag | E90* | PTC | AA->TER | Yes |

| Chr | Position | SNP ID | Accession no. | H-Inv ID | cDNA Pos. | Codon change | AA change | Effect | Inferred direction | NMD |
| --- | --- | --- | --- | --- | --- | --- | --- | --- | --- | --- |
| 16 | 5074830 | rs1047747 | BC031873 | HIT000092460 | 2109,- | Cga/Tga | R403* | PTC | NA | No |
| 16 | 6900691 | rs8057643 | AL162011 | HIT000026197 | 4151,+ | Caa/Taa | Q27* | PTC | AA->TER | No |
| 16 | 7701112 | rs8062475 | CR621423 | HIT000300618 | 1390,- | Tag/Cag | *67Q | Read-through | TER->AA | NA |
| 16 | 10135190 | rs7189525 | BC047499 | HIT000098628 | 493,+ | Taa/Caa | *60Q | Read-through | AA->TER | NA |
| 16 | 11274644 | rs438289 | NM_021247 | HIT000317538 | 310,+ | Tga/Cga | *104R | Read-through | TER->AA | NA |
| 16 | 13949123 | rs2020959 | U64315 | HIT000220801 | 2151,+ | tgC/tgA | C712* | PTC | AA->TER | No |
| 16 | 21234774 | rs12446322 | BC056679 | HIT000259575 | 284,+ | tgG/tgA | W14* | PTC | AA->TER | Yes |
| 16 | 21731162 | rs1063862 | BC009198 | HIT000034194 | 625,+ | Tag/Cag | *209Q | Read-through | NA | NA |
| 16 | 21738149 | rs552855 | BC009198 | HIT000034194 | 271,+ | Caa/Taa | Q91* | PTC | NA | Yes |
| 16 | 23299623 | rs13306630 | BC036352 | HIT000051676 | 2058,+ | taA/taT | *641Y | Read-through | TER->AA | NA |
| 16 | 23476878 | rs11546237 | AK124139 | HIT000044012 | 132,+ | Cga/Tga | R8* | PTC | AA->TER | Yes |
| 16 | 28829772 | rs11545703 | BC046118 | HIT000098262 | 1069,+ | Cag/Tag | Q343* | PTC | AA->TER | Yes |
| 16 | 30273069 | rs11547274 | AF104222 | HIT000069408 | 274,+ | Gag/Tag | E52* | PTC | AA->TER | Yes |
| 16 | 30980108 | rs7187220 | BC021997 | HIT000039338 | 2189,- | Cag/Tag | Q548* | PTC | AA->TER | No |
| 16 | 31705654 | rs2359223 | AK090569 | HIT000015436 | 2776,+ | Caa/Taa | Q28* | PTC | TER->AA | No |
| 16 | 32298168 | rs4045305 | ENST00000360199 | HIT000313620 | 651,+ | tgG/tgA | W211* | PTC | AA->TER | Yes |
| 16 | 33312184 | rs6565349 | ENST00000355251 | HIT000309419 | 961,- | Cag/Tag | Q321* | PTC | AA->TER | Yes |
| 16 | 33332288 | rs2632823 | ENST00000355251 | HIT000309419 | 1654,- | Cag/Tag | Q552* | PTC | AA->TER | No |
| 16 | 33520331 | rs2067108 | ENST00000333662 | HIT000128479 | 1570,- | Cga/Tga | R524* | PTC | AA->TER | No |

| Chr | Position | SNP ID | Accession no. | H-Inv ID | cDNA Pos. | Codon change | AA change | Effect | Inferred direction | NMD |
| --- | --- | --- | --- | --- | --- | --- | --- | --- | --- | --- |
| 16 | 33537474 | rs2002923 | ENST00000355807 | HIT000309895 | 141,+ | tgG/tgA | W47* | PTC | AA->TER | No |
| 16 | 33868594 | rs11863617 | AY587847 | HIT000255645 | 1411,- | Cga/Tga | R450* | PTC | NA | No |
| 16 | 34569938 | rs11541272 | BC023607 | HIT000090808 | 918,+ | Gag/Tag | E62* | PTC | AA->TER | No |
| 16 | 47188573 | rs1825681 | AK024901 | HIT000008175 | 1176,- | tGg/tAg | W47* | PTC | AA->TER | No |
| 16 | 54289322 | rs17855051 | M65105 | HIT000196161 | 1333,+ | Gag/Tag | E425* | PTC | AA->TER | Yes |
| 16 | 54417656 | rs5023780 | AB119995 | HIT000242405 | 371,- | Cga/Tga | R104* | PTC | AA->TER | Yes |
| 16 | 54461055 | rs13338754 | AK090997 | HIT000015864 | 595,- | Cag/Tag | Q123* | PTC | AA->TER | Yes |
| 16 | 55092275 | rs17411455 | AF342736 | HIT000077994 | 1810,+ | taC/taA | Y463* | PTC | NA | Yes |
| 16 | 57341304 | rs12325516 | AK057513 | HIT000014127 | 264,+ | Cga/Tga | R65* | PTC | AA->TER | Yes |
| 16 | 57341483 | rs12925419 | AK057513 | HIT000014127 | 450,+ | Cga/Tga | R127* | PTC | AA->TER | Yes |
| 16 | 65003229 | rs4552024 | AK056683 | HIT000013297 | 1837,+ | tgG/tgA | W20* | PTC | AA->TER | No |
| 16 | 65481319 | rs28739533 | AK098273 | HIT000023103 | 1179,- | Cga/Tga | R117* | PTC | AA->TER | No |
| 16 | 65529578 | rs28382815 | BC032095 | HIT000051049 | 1390,+ | Cga/Tga | R136* | PTC | AA->TER | Yes |
| 16 | 66025220 | rs2549643 | AF370400 | HIT000242956 | 1125,+ | tCa/tGa | S88* | PTC | AA->TER | No |
| 16 | 68548464 | rs2549127 | AK096763 | HIT000021618 | 1674,- | Aga/Tga | R22* | PTC | NA | No |
| 16 | 68812132 | rs3972034 | AK128245 | HIT000048118 | 1589,- | tAa/tCa | *186S | Read-through | NA | NA |
| 16 | 69103768 | rs11542597 | AB088369 | HIT000061464 | 601,+ | Gag/Tag | E201* | PTC | AA->TER | Yes |
| 16 | 69251501 | rs4985556 | BC029804 | HIT000040881 | 1022,+ | taC/taA | Y213* | PTC | AA->TER | No |
| 16 | 69618996 | rs1022220 | AK022933 | HIT000006207 | 3170,- | Tag/Cag | *1018Q | Read-through | TER->AA | NA |

| Chr | Position | SNP ID | Accession no. | H-Inv ID | cDNA Pos. | Codon change | AA change | Effect | Inferred direction | NMD |
| --- | --- | --- | --- | --- | --- | --- | --- | --- | --- | --- |
| 16 | 70558637 | rs4788587 | AY164485 | HIT000250871 | 2365,- | Cga/Tga | R789* | PTC | AA->TER | Yes |
| 16 | 71473874 | rs3812982 | AL359599 | HIT000026327 | 2002,+ | tCg/tAg | S81* | PTC | AA->TER | No |
| 16 | 71549718 | rs13333232 | L32832 | HIT000193060 | 2501,- | Gag/Tag | E610* | PTC | AA->TER | Yes |
| 16 | 71650541 | rs12934016 | L32833 | HIT000193061 | 697,+ | taG/taC | *215Y | Read-through | TER->AA | NA |
| 16 | 74181141 | rs7196038 | BX648197 | HIT000056844 | 1598,- | Cga/Tga | R110* | PTC | AA->TER | No |
| 16 | 79740955 | rs3937686 | AY164483 | HIT000250870 | 4594,+ | Gga/Tga | G1532* | PTC | AA->TER | Yes |
| 16 | 79757045 | rs12925771 | AY164483 | HIT000250870 | 3118,- | Cga/Tga | R1040* | PTC | NA | Yes |
| 16 | 79799699 | rs7499011 | AY164483 | HIT000250870 | 658,- | Cag/Tag | Q220* | PTC | AA->TER | Yes |
| 16 | 79904478 | rs4500719 | AK024241 | HIT000007515 | 52,- | tGg/tAg | W17* | PTC | AA->TER | No |
| 16 | 80689541 | rs8191246 | CR591737 | HIT000270932 | 1327,+ | tAg/tGg | *388W | Read-through | TER->AA | NA |
| 16 | 82557093 | rs13299 | AY258066 | HIT000251272 | 2046,- | Gag/Tag | E555* | PTC | AA->TER | No |
| 16 | 83510144 | rs11647743 | AK000958 | HIT000003432 | 1661,+ | Tga/Cga | *97R | Read-through | TER->AA | NA |
| 16 | 84678241 | rs7499836 | CR627169 | HIT000306094 | 1706,- | Aaa/Taa | K48* | PTC | AA->TER | Yes |
| 16 | 85159945 | rs11640590 | ENST00000320354 | HIT000122393 | 1503,+ | taC/taA | Y501* | PTC | AA->TER | No |
| 16 | 86435573 | rs4843270 | CR936746 | HIT000307019 | 5976,- | Gga/Tga | G159* | PTC | AA->TER | No |
| 16 | 87788983 | rs2270416 | D83542 | HIT000101805 | 2441,- | taC/taA | Y788* | PTC | TER->AA | No |
| 16 | 88126503 | rs17850240 | Y16610 | HIT000325941 | 1294,+ | Gag/Tag | E428* | PTC | AA->TER | Yes |
| 16 | 88505004 | rs17408173 | BC011884 | HIT000035513 | 1944,+ | Gaa/Taa | E630* | PTC | AA->TER | No |
| 16 | 88529672 | rs11554952 | CR598025 | HIT000277220 | 1399,+ | Gaa/Taa | E438* | PTC | AA->TER | No |

| Chr | Position | SNP ID | Accession no. | H-Inv ID | cDNA Pos. | Codon change | AA change | Effect | Inferred direction | NMD |
| --- | --- | --- | --- | --- | --- | --- | --- | --- | --- | --- |
| 17 | 181103 | rs9911905 | BC027928 | HIT000091411 | 55,+ | tCa/tGa | S13* | PTC | TER->AA | Yes |
| 17 | 368066 | rs1060227 | AF085927 | HIT000066806 | 436,+ | tgG/tgA | W114* | PTC | TER->AA | No |
| 17 | 791584 | rs7215387 | AK130278 | HIT000049549 | 1145,- | tTg/tAg | L375* | PTC | NA | Yes |
| 17 | 793008 | rs12937651 | AK130278 | HIT000049549 | 120,- | taT/taG | Y33* | PTC | TER->AA | Yes |
| 17 | 1708196 | rs11656253 | ENST00000226102 | HIT000103962 | 295,- | Cga/Tga | R99* | PTC | NA | No |
| 17 | 3142353 | rs7218125 | BC069414 | HIT000263565 | 274,- | Aag/Tag | K92* | PTC | AA->TER | No |
| 17 | 3366514 | rs11654533 | AF514998 | HIT000081099 | 2249,- | Cga/Tga | R729* | PTC | AA->TER | Yes |
| 17 | 3801717 | rs17846878 | BC035729 | HIT000051491 | 359,+ | tgG/tgA | W77* | PTC | AA->TER | Yes |
| 17 | 4408497 | rs7215121 | AK129501 | HIT000048772 | 498,- | Tga/Cga | *152R | Read-through | TER->AA | NA |
| 17 | 4488681 | rs11870258 | M23892 | HIT000194905 | 593,- | tGg/tAg | W197* | PTC | AA->TER | Yes |
| 17 | 4778076 | rs12948309 | ENST00000329125 | HIT000125946 | 1397,+ | tTa/tAa | L466* | PTC | AA->TER | No |
| 17 | 7327004 | rs7214088 | ENST00000331053 | HIT000127012 | 977,+ | tGg/tAg | W326* | PTC | NA | No |
| 17 | 11836724 | rs11545759 | AK092656 | HIT000017523 | 753,+ | Cag/Tag | Q50* | PTC | AA->TER | Yes |
| 17 | 15631463 | rs534998 | AK054814 | HIT000011428 | 574,- | tCa/tGa | S191* | PTC | NA | No |
| 17 | 16644205 | rs637755 | BC063453 | HIT000260913 | 776,+ | Cga/Tga | R92* | PTC | NA | Yes |
| 17 | 19549365 | rs17853411 | AK055758 | HIT000012372 | 942,- | Tga/Cga | *296R | Read-through | TER->AA | NA |
| 17 | 20434004 | rs603525 | ENST00000340731 | HIT000133361 | 807,- | tgG/tgA | W269* | PTC | NA | No |
| 17 | 20709322 | rs4605228 | BC068597 | HIT000263152 | 784,+ | Taa/Caa | *262Q | Read-through | AA->TER | NA |
| 17 | 26250373 | rs3192548 | AJ314645 | HIT000247141 | 1060,+ | taT/taA | Y345* | PTC | AA->TER | No |

| Chr | Position | SNP ID | Accession no. | H-Inv ID | cDNA Pos. | Codon change | AA change | Effect | Inferred direction | NMD |
| --- | --- | --- | --- | --- | --- | --- | --- | --- | --- | --- |
| 17 | 27291444 | rs2627175 | D63881 | HIT000042297 | 495,- | Cga/Tga | R101* | PTC | AA->TER | Yes |
| 17 | 31967671 | rs12952744 | AK094752 | HIT000019607 | 663,+ | tTg/tAg | L203* | PTC | AA->TER | No |
| 17 | 32759994 | rs28364048 | AY315624 | HIT000251690 | 104,+ | Cag/Tag | Q22* | PTC | AA->TER | No |
| 17 | 34872333 | rs9896487 | AF227198 | HIT000074461 | 516,+ | taT/taG | Y161* | PTC | AA->TER | Yes |
| 17 | 36587744 | rs28457257 | AJ406934 | HIT000247504 | 243,- | Cag/Tag | Q67* | PTC | AA->TER | No |
| 17 | 37232184 | rs11540883 | BC016467 | HIT000037576 | 1807,+ | Tga/Gga | *583G | Read-through | TER->AA | NA |
| 17 | 37528641 | rs1055231 | AK093488 | HIT000018355 | 1668,+ | Cag/Tag | Q83* | PTC | AA->TER | No |
| 17 | 38257563 | rs629682 | U39447 | HIT000219688 | 837,- | tGg/tAg | W226* | PTC | AA->TER | Yes |
| 17 | 39609781 | rs7224330 | AK055685 | HIT000012299 | 1299,- | tgG/tgA | W210* | PTC | AA->TER | No |
| 17 | 40116796 | rs11549083 | BC023298 | HIT000090755 | 267,+ | Cag/Tag | Q88* | PTC | AA->TER | Yes |
| 17 | 41329788 | rs8079933 | AK091698 | HIT000016565 | 599,+ | Gga/Tga | G87* | PTC | AA->TER | No |
| 17 | 43413096 | rs1043474 | CR605714 | HIT000284909 | 1318,+ | Gag/Tag | E250* | PTC | AA->TER | Yes |
| 17 | 45164967 | rs11539791 | BC037572 | HIT000051940 | 392,+ | tGg/tAg | W104* | PTC | AA->TER | Yes |
| 17 | 45567301 | rs11553931 | CR605734 | HIT000284929 | 986,- | Cag/Tag | Q127* | PTC | AA->TER | No |
| 17 | 47194799 | rs875510 | AK131515 | HIT000249803 | 958,+ | Cag/Tag | Q29* | PTC | AA->TER | No |
| 17 | 52280368 | rs12601812 | U49379 | HIT000220095 | 918,+ | tgT/tgA | C277* | PTC | AA->TER | Yes |
| 17 | 52416354 | rs4794677 | AK124440 | HIT000044313 | 1112,- | tGg/tAg | W77* | PTC | AA->TER | No |
| 17 | 52710719 | rs12604002 | AK021772 | HIT000005046 | 440,+ | Cga/Tga | R16* | PTC | AA->TER | No |
| 17 | 55129755 | rs11267 | AK057033 | HIT000013647 | 1743,- | Gaa/Taa | E124* | PTC | AA->TER | No |

| Chr | Position | SNP ID | Accession no. | H-Inv ID | cDNA Pos. | Codon change | AA change | Effect | Inferred direction | NMD |
| --- | --- | --- | --- | --- | --- | --- | --- | --- | --- | --- |
| 17 | 55640966 | rs2627859 | AF533230 | HIT000081343 | 2776,- | tgG/tgA | W868* | PTC | NA | Yes |
| 17 | 57022735 | rs17610181 | BC062710 | HIT000260601 | 615,- | Cga/Tga | R197* | PTC | AA->TER | No |
| 17 | 57700290 | rs17857479 | BC058890 | HIT000259900 | 844,- | Cag/Tag | Q254* | PTC | AA->TER | Yes |
| 17 | 58156162 | rs17857042 | BX648705 | HIT000057352 | 2372,- | tTg/tAg | L696* | PTC | AA->TER | Yes |
| 17 | 59134837 | rs9915192 | AF308302 | HIT000076927 | 1263,- | Cga/Tga | R384* | PTC | AA->TER | No |
| 17 | 59288515 | rs2584610 | AB026156 | HIT000058846 | 408,- | Cag/Tag | Q116* | PTC | AA->TER | Yes |
| 17 | 59608490 | rs17853690 | BC040521 | HIT000052413 | 2604,- | tGg/tAg | W840* | PTC | AA->TER | Yes |
| 17 | 60211869 | rs3186950 | BC041324 | HIT000096613 | 2677,+ | tgA/tgG | *521W | Read-through | TER->AA | NA |
| 17 | 60211871 | rs3186948 | BC041324 | HIT000096613 | 2675,+ | Tga/Cga | *521R | Read-through | TER->AA | NA |
| 17 | 61865500 | rs9898333 | AK129693 | HIT000048964 | 302,+ | Caa/Taa | Q53* | PTC | AA->TER | No |
| 17 | 62890090 | rs7223459 | AK057988 | HIT000014595 | 382,- | Cga/Tga | R127* | PTC | AA->TER | No |
| 17 | 63714376 | rs12945591 | CR603455 | HIT000282650 | 1152,+ | taC/taA | Y203* | PTC | AA->TER | No |
| 17 | 64661568 | rs10491178 | AL832004 | HIT000026916 | 4638,+ | Cga/Tga | R371* | PTC | AA->TER | Yes |
| 17 | 70100401 | rs545652 | AK056444 | HIT000013058 | 983,- | tgA/tgC | *207C | Read-through | TER->AA | NA |
| 17 | 70528319 | rs8068910 | BC015335 | HIT000037083 | 514,+ | Aaa/Taa | K170* | PTC | AA->TER | No |
| 17 | 71001211 | rs7214333 | D83779 | HIT000042331 | 2334,+ | Gag/Tag | E711* | PTC | AA->TER | Yes |
| 17 | 71895233 | rs3744040 | AK022402 | HIT000005676 | 1518,+ | Cag/Tag | Q376* | PTC | AA->TER | No |
| 17 | 72936969 | rs312897 | AK022252 | HIT000005526 | 478,- | tCa/tGa | S115* | PTC | AA->TER | No |
| 17 | 73231291 | rs16970479 | AK127897 | HIT000047770 | 1539,- | Tga/Cga | *158R | Read-through | AA->TER | NA |

| Chr | Position | SNP ID | Accession no. | H-Inv ID | cDNA Pos. | Codon change | AA change | Effect | Inferred direction | NMD |
| --- | --- | --- | --- | --- | --- | --- | --- | --- | --- | --- |
| 17 | 73231428 | rs2033717 | AK127897 | HIT000047770 | 1402,+ | tCg/tAg | S112* | PTC | AA->TER | No |
| 17 | 76559435 | rs6565507 | AK127919 | HIT000047792 | 174,- | tgG/tgA | W58* | PTC | AA->TER | Yes |
| 17 | 76851439 | rs11867477 | AK124483 | HIT000044356 | 125,+ | tCg/tAg | S33* | PTC | AA->TER | No |
| 17 | 77092875 | rs11549178 | CR619574 | HIT000298769 | 796,+ | Cag/Tag | Q246* | PTC | AA->TER | Yes |
| 17 | 77093685 | rs11549233 | CR619574 | HIT000298769 | 262,+ | Aag/Tag | K68* | PTC | AA->TER | Yes |
| 17 | 77664739 | rs11653662 | AK074334 | HIT000015114 | 690,- | Cga/Tga | R226* | PTC | AA->TER | No |
| 17 | 78034350 | rs17855348 | BC000438 | HIT000029624 | 895,+ | tGg/tAg | W263* | PTC | TER->AA | Yes |
| 17 | 78278226 | rs11551833 | CR590236 | HIT000269431 | 820,+ | Gag/Tag | E274* | PTC | AA->TER | No |
| 18 | 648170 | rs28602966 | BC028301 | HIT000040517 | 171,- | taC/taA | Y26* | PTC | AA->TER | Yes |
| 18 | 899583 | rs11540551 | S83513 | HIT000217215 | 479,+ | Aag/Tag | K160* | PTC | AA->TER | No |
| 18 | 3385125 | rs4392157 | ENST00000328419 | HIT000125542 | 177,- | tgG/tgA | W59* | PTC | AA->TER | No |
| 18 | 11656548 | rs9807408 | BC034640 | HIT000093523 | 3617,- | Tga/Cga | *159R | Read-through | AA->TER | NA |
| 18 | 14233212 | rs12326588 | ENST00000338190 | HIT000130820 | 94,+ | Aaa/Taa | K32* | PTC | NA | Yes |
| 18 | 14830629 | rs5015694 | AF269088 | HIT000075749 | 2854,+ | Gag/Tag | E892* | PTC | NA | Yes |
| 18 | 17457736 | rs11548769 | CR613811 | HIT000293006 | 275,+ | Aaa/Taa | K41* | PTC | AA->TER | Yes |
| 18 | 18001213 | rs11874782 | AK123079 | HIT000042952 | 1432,- | tgC/tgA | C16* | PTC | AA->TER | No |
| 18 | 22026329 | rs17852262 | BC028371 | HIT000040540 | 697,+ | Gaa/Taa | E199* | PTC | AA->TER | No |
| 18 | 41920164 | rs11541934 | AK129739 | HIT000049010 | 1407,+ | Cag/Tag | Q448* | PTC | AA->TER | Yes |
| 18 | 42428364 | rs668003 | AK127869 | HIT000047742 | 511,- | Gag/Tag | E122* | PTC | AA->TER | Yes |

| Chr | Position | SNP ID | Accession no. | H-Inv ID | cDNA Pos. | Codon change | AA change | Effect | Inferred direction | NMD |
| --- | --- | --- | --- | --- | --- | --- | --- | --- | --- | --- |
| 18 | 44442335 | rs299748 | CR596894 | HIT000276089 | 127,- | Cag/Tag | Q25* | PTC | AA->TER | No |
| 18 | 50134887 | rs17292725 | AF480305 | HIT000080450 | 55,- | Cga/Tga | R19* | PTC | AA->TER | Yes |
| 18 | 52006523 | rs12604483 | AK127645 | HIT000047518 | 556,- | taT/taA | Y64* | PTC | AA->TER | Yes |
| 18 | 52417963 | rs688992 | BC034791 | HIT000093560 | 685,+ | taT/taG | Y60* | PTC | AA->TER | Yes |
| 18 | 54803906 | rs17065360 | CR600377 | HIT000279572 | 767,- | Gaa/Taa | E22* | PTC | AA->TER | No |
| 18 | 55828833 | rs11152199 | ENST00000341108 | HIT000133738 | 523,- | Gag/Tag | E175* | PTC | AA->TER | No |
| 18 | 56464221 | rs17067194 | ENST00000355336 | HIT000309494 | 1420,+ | Cga/Tga | R474* | PTC | AA->TER | No |
| 18 | 59530818 | rs4940595 | AF419953 | HIT000079376 | 268,+ | Gaa/Taa | E90* | PTC | TER->AA | Yes |
| 18 | 70208398 | rs11553060 | ENST00000333971 | HIT000128665 | 127,+ | Caa/Taa | Q39* | PTC | AA->TER | Yes |
| 18 | 72282564 | rs12964858 | D86975 | HIT000042357 | 1753,- | Aag/Tag | K479* | PTC | AA->TER | Yes |
| 19 | 597891 | rs10403235 | AK127589 | HIT000047462 | 419,- | Cga/Tga | R53* | PTC | AA->TER | Yes |
| 19 | 2379834 | rs1049923 | AK129641 | HIT000048912 | 670,- | tTa/tGa | L5* | PTC | NA | Yes |
| 19 | 2665637 | rs8212 | AK124445 | HIT000044318 | 69,+ | Cga/Tga | R22* | PTC | AA->TER | No |
| 19 | 3546033 | rs8113293 | U11271 | HIT000218039 | 1236,- | Cga/Tga | R342* | PTC | AA->TER | No |
| 19 | 4308555 | rs17853076 | AK024930 | HIT000008204 | 891,- | Cga/Tga | R50* | PTC | AA->TER | No |
| 19 | 4439805 | rs1063176 | AF294267 | HIT000242863 | 483,+ | Gag/Tag | E141* | PTC | AA->TER | Yes |
| 19 | 6616128 | rs17851607 | AF064090 | HIT000065389 | 914,- | Gag/Tag | E178* | PTC | AA->TER | No |
| 19 | 8273218 | rs2232788 | AF161254 | HIT000072066 | 1043,+ | Cga/Tga | R156* | PTC | AA->TER | No |
| 19 | 9098263 | rs17001893 | NM_001001958 | HIT000315568 | 364,- | Cga/Tga | R122* | PTC | AA->TER | No |

| Chr | Position | SNP ID | Accession no. | H-Inv ID | cDNA Pos. | Codon change | AA change | Effect | Inferred direction | NMD |
| --- | --- | --- | --- | --- | --- | --- | --- | --- | --- | --- |
| 19 | 11366165 | rs13344609 | AK126263 | HIT000046136 | 427,+ | Gaa/Taa | E88* | PTC | AA->TER | No |
| 19 | 11421226 | rs17415169 | J03075 | HIT000191127 | 1719,+ | tAg/tTg | *528L | Read-through | TER->AA | NA |
| 19 | 11421227 | rs17411469 | J03075 | HIT000191127 | 1720,+ | taG/taT | *528Y | Read-through | TER->AA | NA |
| 19 | 12048861 | rs10426017 | AL832297 | HIT000027209 | 2087,+ | Taa/Gaa | *263E | Read-through | AA->TER | NA |
| 19 | 12362281 | rs2006651 | AK027660 | HIT000010932 | 2585,+ | tAg/tTg | *612L | Read-through | NA | NA |
| 19 | 12401971 | rs28559848 | AB011414 | HIT000058279 | 2119,- | tAg/tTg | *672L | Read-through | NA | NA |
| 19 | 12926162 | rs11538996 | AF479749 | HIT000080439 | 553,+ | Gag/Tag | E177* | PTC | AA->TER | No |
| 19 | 15600230 | rs4646532 | AF133298 | HIT000070969 | 1241,+ | Cga/Tga | R412* | PTC | AA->TER | Yes |
| 19 | 16047505 | rs1862601 | AK056565 | HIT000013179 | 1704,+ | tAa/tCa | *292S | Read-through | TER->AA | NA |
| 19 | 16481328 | rs3826726 | AK025395 | HIT000008669 | 1222,+ | Caa/Taa | Q390* | PTC | NA | Yes |
| 19 | 17258359 | rs4995358 | AK092706 | HIT000017573 | 1812,+ | Tga/Gga | *576G | Read-through | TER->AA | NA |
| 19 | 17278033 | rs16981720 | BC021801 | HIT000039287 | 348,+ | Cag/Tag | Q42* | PTC | AA->TER | No |
| 19 | 17365598 | rs10404192 | BC019872 | HIT000089983 | 762,- | Aga/Tga | R124* | PTC | AA->TER | Yes |
| 19 | 18358162 | rs16982331 | AB000584 | HIT000057919 | 195,+ | Gag/Tag | E55* | PTC | AA->TER | Yes |
| 19 | 18408855 | rs11552754 | BC066902 | HIT000262323 | 391,+ | Gag/Tag | E115* | PTC | AA->TER | Yes |
| 19 | 19068010 | rs12983184 | BC045598 | HIT000052939 | 230,+ | tCa/tAa | S26* | PTC | AA->TER | Yes |
| 19 | 21782555 | rs1049169 | CR933640 | HIT000306822 | 2767,+ | tgT/tgA | C715* | PTC | AA->TER | No |
| 19 | 22113113 | rs3813590 | ENST00000310882 | HIT000118854 | 364,- | Caa/Taa | Q122* | PTC | AA->TER | No |
| 19 | 23714876 | rs7249699 | CR624679 | HIT000303874 | 697,- | tTa/tAa | L45* | PTC | AA->TER | No |

| Chr | Position | SNP ID | Accession no. | H-Inv ID | cDNA Pos. | Codon change | AA change | Effect | Inferred direction | NMD |
| --- | --- | --- | --- | --- | --- | --- | --- | --- | --- | --- |
| 19 | 34611487 | rs8107220 | AK094793 | HIT000019648 | 403,- | Cga/Tga | R84* | PTC | AA->TER | Yes |
| 19 | 39808342 | rs10425319 | AK125967 | HIT000045840 | 2664,+ | Gga/Tga | G68* | PTC | TER->AA | No |
| 19 | 39859530 | rs10413838 | CR603799 | HIT000282994 | 1069,+ | Taa/Gaa | *79E | Read-through | TER->AA | NA |
| 19 | 40410779 | rs13382163 | AK098526 | HIT000023356 | 690,- | taC/taG | Y215* | PTC | AA->TER | Yes |
| 19 | 40410860 | rs541169 | AK098526 | HIT000023356 | 609,+ | tgG/tgA | W188* | PTC | AA->TER | Yes |
| 19 | 42002191 | rs1227794 | BC057245 | HIT000259670 | 1018,- | Cga/Tga | R299* | PTC | AA->TER | Yes |
| 19 | 46719354 | rs4803476 | ENST00000320909 | HIT000122602 | 769,- | Aaa/Taa | K257* | PTC | NA | No |
| 19 | 47258509 | rs12609687 | S40369 | HIT000215576 | 479,- | tCg/tAg | S160* | PTC | AA->TER | Yes |
| 19 | 48064874 | rs12986075 | BC058285 | HIT000259841 | 924,- | Cga/Tga | R288* | PTC | NA | Yes |
| 19 | 48449601 | rs1058538 | CR623240 | HIT000302435 | 1374,+ | tgA/tgG | *427W | Read-through | TER->AA | NA |
| 19 | 48449605 | rs1062984 | CR623240 | HIT000302435 | 1370,+ | tCa/tAa | S426* | PTC | AA->TER | No |
| 19 | 48757006 | rs25495 | M36089 | HIT000195661 | 699,+ | Aag/Tag | K51* | PTC | AA->TER | Yes |
| 19 | 49019355 | rs9917033 | AK131520 | HIT000249808 | 760,- | tCa/tAa | S93* | PTC | AA->TER | No |
| 19 | 50139910 | rs5164 | AK126257 | HIT000046130 | 181,+ | tgG/tgA | W47* | PTC | AA->TER | Yes |
| 19 | 50491815 | rs2098088 | AK022239 | HIT000005513 | 332,- | tCa/tGa | S69* | PTC | AA->TER | No |
| 19 | 50883603 | rs12424 | U15008 | HIT000218293 | 94,+ | Gag/Tag | E22* | PTC | AA->TER | Yes |
| 19 | 50906783 | rs17855490 | BC008046 | HIT000050493 | 1510,- | tGa/tCa | *462S | Read-through | AA->TER | NA |
| 19 | 51397966 | rs28798191 | BC062328 | HIT000260329 | 194,- | Cga/Tga | R9* | PTC | NA | Yes |
| 19 | 53429518 | rs2043211 | AB023172 | HIT000000672 | 343,- | tgT/tgA | C10* | PTC | AA->TER | Yes |

| Chr | Position | SNP ID | Accession no. | H-Inv ID | cDNA Pos. | Codon change | AA change | Effect | Inferred direction | NMD |
| --- | --- | --- | --- | --- | --- | --- | --- | --- | --- | --- |
| 19 | 53537872 | rs16982007 | AK129801 | HIT000049072 | 722,- | taC/taG | Y234* | PTC | AA->TER | Yes |
| 19 | 53898415 | rs281377 | BC001899 | HIT000030730 | 451,+ | Cga/Tga | R151* | PTC | AA->TER | Yes |
| 19 | 53898538 | rs28362838 | BC001899 | HIT000030730 | 574,+ | Cga/Tga | R192* | PTC | AA->TER | Yes |
| 19 | 54137586 | rs10423255 | BC032730 | HIT000041697 | 748,+ | Cag/Tag | Q233* | PTC | AA->TER | Yes |
| 19 | 55246050 | rs10422147 | AK130360 | HIT000049631 | 393,+ | tgG/tgA | W93* | PTC | AA->TER | Yes |
| 19 | 55528943 | rs11879785 | BC019048 | HIT000089865 | 1269,- | tgC/tgA | C421* | PTC | TER->AA | No |
| 19 | 55597741 | rs11550555 | M80397 | HIT000196429 | 954,+ | Gaa/Taa | E301* | PTC | AA->TER | Yes |
| 19 | 56320178 | rs1697545 | AF227924 | HIT000074481 | 202,+ | taC/taA | Y45* | PTC | AA->TER | Yes |
| 19 | 56696715 | rs16982743 | AK095919 | HIT000020774 | 141,- | Cag/Tag | Q29* | PTC | AA->TER | Yes |
| 19 | 56787865 | rs3794983 | AK054965 | HIT000011579 | 681,+ | Aaa/Taa | K209* | PTC | TER->AA | No |
| 19 | 56822743 | rs8108078 | U71383 | HIT000221160 | 1208,- | Cga/Tga | R356* | PTC | AA->TER | Yes |
| 19 | 57496660 | rs321914 | AK097759 | HIT000022613 | 1003,+ | Cga/Tga | R87* | PTC | AA->TER | No |
| 19 | 59396925 | rs11544026 | CR616829 | HIT000296024 | 176,+ | Gag/Tag | E21* | PTC | AA->TER | Yes |
| 19 | 60033405 | rs1130516 | X94262 | HIT000324854 | 1199,+ | Cag/Tag | Q400* | PTC | NA | No |
| 19 | 61191091 | rs306457 | AY154463 | HIT000084795 | 3090,- | taG/taC | *1030Y | Read-through | TER->AA | NA |
| 19 | 62334594 | rs9973206 | AF229438 | HIT000074530 | 2772,+ | taC/taA | Y913* | PTC | TER->AA | No |
| 19 | 62755668 | rs28547388 | AL833090 | HIT000028002 | 451,+ | taT/taG | Y101* | PTC | AA->TER | No |
| 20 | 1827313 | rs1998060 | AK075122 | HIT000082157 | 539,- | taA/taT | *91Y | Read-through | AA->TER | NA |
| 20 | 2684445 | rs17854869 | AB037863 | HIT000001157 | 1847,+ | Cag/Tag | Q529* | PTC | AA->TER | Yes |

| Chr | Position | SNP ID | Accession no. | H-Inv ID | cDNA Pos. | Codon change | AA change | Effect | Inferred direction | NMD |
| --- | --- | --- | --- | --- | --- | --- | --- | --- | --- | --- |
| 20 | 4124275 | rs6084659 | BC069037 | HIT000263220 | 357,+ | taG/taT | *69Y | Read-through | TER->AA | NA |
| 20 | 5169180 | rs6133196 | AK124694 | HIT000044567 | 2612,- | Cga/Tga | R113* | PTC | AA->TER | No |
| 20 | 8646452 | rs13042803 | AB011153 | HIT000000298 | 1857,+ | taC/taA | Y490* | PTC | AA->TER | Yes |
| 20 | 9983110 | rs13045682 | AK097689 | HIT000022543 | 2347,+ | Gaa/Taa | E679* | PTC | AA->TER | Yes |
| 20 | 19906375 | rs199578 | AK130346 | HIT000049617 | 383,+ | tGg/tAg | W90* | PTC | TER->AA | No |
| 20 | 19964904 | rs1046346 | AF255443 | HIT000075137 | 2478,+ | Cag/Tag | Q816* | PTC | AA->TER | No |
| 20 | 21090666 | rs11553176 | AK025476 | HIT000008750 | 624,+ | tCa/tGa | S187* | PTC | AA->TER | Yes |
| 20 | 23752714 | rs13041691 | BC062679 | HIT000260570 | 442,- | taC/taA | Y123* | PTC | AA->TER | No |
| 20 | 23753918 | rs6049157 | BC062679 | HIT000260570 | 344,- | Cga/Tga | R91* | PTC | NA | Yes |
| 20 | 25701542 | rs2248738 | AK054844 | HIT000011458 | 538,- | tGg/tAg | W25* | PTC | AA->TER | Yes |
| 20 | 28242022 | rs11152457 | CR933606 | HIT000306798 | 1030,+ | tgC/tgA | C147* | PTC | AA->TER | Yes |
| 20 | 30052171 | rs6087809 | AK123879 | HIT000043752 | 2681,- | Aga/Tga | R74* | PTC | AA->TER | No |
| 20 | 33331233 | rs11553027 | AF047433 | HIT000001733 | 542,+ | Gga/Tga | G158* | PTC | AA->TER | Yes |
| 20 | 33901997 | rs17093156 | NR_000032 | HIT000320638 | 255,- | tgA/tgG | *85W | Read-through | TER->AA | NA |
| 20 | 34005185 | rs11538234 | BC041022 | HIT000096533 | 1507,- | Cag/Tag | Q146* | PTC | AA->TER | No |
| 20 | 34307171 | rs11905241 | CR610727 | HIT000289922 | 1074,- | tgG/tgA | W42* | PTC | AA->TER | No |
| 20 | 35241114 | rs11539065 | BC030006 | HIT000092004 | 298,- | tgG/tgA | W71* | PTC | AA->TER | Yes |
| 20 | 36073646 | rs1057237 | BC013121 | HIT000036134 | 2196,+ | Caa/Taa | Q663* | PTC | AA->TER | Yes |
| 20 | 36278370 | rs12480600 | AK094947 | HIT000019802 | 161,- | tgG/tgA | W17* | PTC | AA->TER | No |

| Chr | Position | SNP ID | Accession no. | H-Inv ID | cDNA Pos. | Codon change | AA change | Effect | Inferred direction | NMD |
| --- | --- | --- | --- | --- | --- | --- | --- | --- | --- | --- |
| 20 | 43270193 | rs2233885 | J04440 | HIT000191260 | 847,+ | Cga/Tga | R281* | PTC | AA->TER | Yes |
| 20 | 43366762 | rs2233091 | AK074593 | HIT000081628 | 408,+ | Cag/Tag | Q55* | PTC | AA->TER | Yes |
| 20 | 43940042 | rs11557696 | BC027611 | HIT000040288 | 1640,- | Cag/Tag | Q480* | PTC | AA->TER | No |
| 20 | 44507949 | rs3092741 | AK054905 | HIT000011519 | 2465,+ | tGg/tAg | W73* | PTC | NA | No |
| 20 | 47328625 | rs28362646 | AY513722 | HIT000255078 | 504,- | tAg/tGg | *118W | Read-through | TER->AA | NA |
| 20 | 47564255 | rs13306027 | D38145 | HIT000101420 | 967,+ | Gag/Tag | E314* | PTC | AA->TER | Yes |
| 20 | 48132036 | rs6095755 | CR615526 | HIT000294721 | 977,+ | Gag/Tag | E38* | PTC | NA | No |
| 20 | 54505879 | rs6024911 | AK131203 | HIT000249491 | 899,- | Caa/Taa | Q65* | PTC | AA->TER | Yes |
| 20 | 55179422 | rs6070006 | X51801 | HIT000321697 | 1418,- | taG/taT | *432Y | Read-through | AA->TER | NA |
| 20 | 55400202 | rs11546708 | AF432218 | HIT000079604 | 339,+ | taC/taG | Y53* | PTC | AA->TER | Yes |
| 20 | 55497442 | rs6015012 | NM_001008735 | HIT000316586 | 48,- | taT/taA | Y16* | PTC | NA | No |
| 20 | 56241375 | rs3818744 | AF119843 | HIT000070230 | 1566,- | Cga/Tga | R343* | PTC | TER->AA | Yes |
| 20 | 56627603 | rs8116576 | AK054637 | HIT000011251 | 1347,+ | Cag/Tag | Q77* | PTC | AA->TER | No |
| 20 | 58077280 | rs6027272 | AY358539 | HIT000252583 | 318,+ | Cga/Tga | R71* | PTC | AA->TER | No |
| 20 | 59947802 | rs6061389 | AK092092 | HIT000016959 | 1228,+ | Cag/Tag | Q96* | PTC | AA->TER | No |
| 20 | 60016024 | rs4414752 | Y11354 | HIT000325652 | 1942,- | Caa/Taa | Q648* | PTC | AA->TER | Yes |
| 20 | 60337317 | rs6143022 | AF443072 | HIT000079754 | 4492,- | taC/taG | Y1475* | PTC | AA->TER | Yes |
| 20 | 61069169 | rs17856725 | BC025312 | HIT000039893 | 1369,+ | Tag/Gag | *457E | Read-through | AA->TER | NA |
| 20 | 61964515 | rs17852993 | BC031949 | HIT000041391 | 1221,+ | tAg/tGg | *393W | Read-through | AA->TER | NA |

| Chr | Position | SNP ID | Accession no. | H-Inv ID | cDNA Pos. | Codon change | AA change | Effect | Inferred direction | NMD |
| --- | --- | --- | --- | --- | --- | --- | --- | --- | --- | --- |
| 20 | 62309963 | rs13041967 | AB020642 | HIT000000552 | 1334,+ | Aag/Tag | K324* | PTC | NA | Yes |
| 21 | 9964627 | rs1810540 | AF007118 | HIT000061860 | 1024,+ | Cga/Tga | R229* | PTC | NA | Yes |
| 21 | 10071492 | rs28441174 | AF218570 | HIT000074290 | 488,- | Cga/Tga | R94* | PTC | NA | Yes |
| 21 | 13332600 | rs462981 | BC036510 | HIT000094455 | 253,+ | Aga/Tga | R12* | PTC | AA->TER | Yes |
| 21 | 13678620 | rs9979106 | ENST00000309142 | HIT000118238 | 1093,- | Cga/Tga | R365* | PTC | NA | No |
| 21 | 26058802 | rs2829898 | BX647755 | HIT000056402 | 1497,+ | tgG/tgA | W323* | PTC | AA->TER | Yes |
| 21 | 26174938 | rs736479 | S41243 | HIT000215584 | 206,- | Cga/Tga | R41* | PTC | TER->AA | No |
| 21 | 30665998 | rs877346 | NM_181621 | HIT000318402 | 405,+ | tgT/tgA | C135* | PTC | AA->TER | No |
| 21 | 30690680 | rs1010682 | AJ457066 | HIT000248063 | 286,+ | tgT/tgA | C95* | PTC | AA->TER | No |
| 21 | 30785949 | rs1048494 | AF139541 | HIT000242736 | 198,+ | tgC/tgA | C66* | PTC | AA->TER | No |
| 21 | 33091188 | rs28662924 | AK096601 | HIT000021456 | 493,+ | tGg/tAg | W68* | PTC | NA | No |
| 21 | 33558551 | rs14861 | AK123722 | HIT000043595 | 1723,- | Cag/Tag | Q32* | PTC | AA->TER | No |
| 21 | 34126290 | rs2834271 | AL117447 | HIT000024609 | 695,+ | tgA/tgG | *113W | Read-through | TER->AA | NA |
| 21 | 34386928 | rs1783302 | AK055913 | HIT000012527 | 2410,- | taG/taC | *94Y | Read-through | AA->TER | NA |
| 21 | 34419562 | rs11557338 | AY061855 | HIT000084037 | 275,+ | Gga/Tga | G33* | PTC | AA->TER | Yes |
| 21 | 34743485 | rs17853625 | AF135188 | HIT000071040 | 318,- | tgC/tgA | C106* | PTC | AA->TER | No |
| 21 | 38450325 | rs2836172 | AJ306840 | HIT000246958 | 456,+ | Aaa/Taa | K58* | PTC | AA->TER | No |
| 21 | 39636455 | rs3959101 | CR591548 | HIT000270743 | 309,+ | tgG/tgA | W31* | PTC | AA->TER | No |
| 21 | 40022959 | rs8130411 | CR624487 | HIT000303682 | 268,+ | Cag/Tag | Q82* | PTC | AA->TER | Yes |

| Chr | Position | SNP ID | Accession no. | H-Inv ID | cDNA Pos. | Codon change | AA change | Effect | Inferred direction | NMD |
| --- | --- | --- | --- | --- | --- | --- | --- | --- | --- | --- |
| 21 | 40677867 | rs2837598 | AF401033 | HIT000079083 | 792,+ | Cag/Tag | Q21* | PTC | AA->TER | Yes |
| 21 | 41446702 | rs11702600 | ENST00000331748 | HIT000127405 | 854,+ | tCa/tGa | S285* | PTC | TER->AA | No |
| 21 | 42281104 | rs693386 | CR594540 | HIT000273735 | 993,- | tCa/tAa | S71* | PTC | TER->AA | No |
| 21 | 42881426 | rs2849723 | ENST00000339657 | HIT000132287 | 955,+ | Cga/Tga | R319* | PTC | NA | No |
| 21 | 43196789 | rs4148974 | CR607646 | HIT000286841 | 610,+ | Cga/Tga | R200* | PTC | AA->TER | Yes |
| 21 | 43608028 | rs2073428 | AK123727 | HIT000043600 | 389,+ | Gag/Tag | E121* | PTC | AA->TER | Yes |
| 21 | 44571555 | rs11700493 | AF147333 | HIT000071406 | 137,- | taG/taT | *39Y | Read-through | AA->TER | NA |
| 21 | 44819269 | rs7276273 | AB076351 | HIT000242040 | 1236,+ | tgA/tgC | *402C | Read-through | AA->TER | NA |
| 21 | 44836011 | rs233303 | AB076353 | HIT000242042 | 804,- | tgC/tgA | C261* | PTC | AA->TER | No |
| 21 | 45243622 | rs8134028 | BC027456 | HIT000091334 | 661,- | Cga/Tga | R77* | PTC | AA->TER | No |
| 21 | 45539742 | rs17329019 | BC063873 | HIT000261249 | 451,+ | tgG/tgA | W5* | PTC | AA->TER | No |
| 21 | 45588725 | rs12329656 | ENST00000215202 | HIT000102894 | 562,- | Cag/Tag | Q188* | PTC | AA->TER | No |
| 21 | 46398518 | rs8133955 | BC052248 | HIT000099479 | 251,- | Cga/Tga | R71* | PTC | AA->TER | Yes |
| 21 | 46485172 | rs2298696 | AJ010089 | HIT000244382 | 5651,- | Gag/Tag | E1872* | PTC | AA->TER | Yes |
| 22 | 15342551 | rs12484275 | AY026350 | HIT000083229 | 105,+ | tgG/tgA | W33* | PTC | NA | No |
| 22 | 15503130 | rs2381064 | AY219889 | HIT000251081 | 1027,+ | tAg/tCg | *127S | Read-through | TER->AA | NA |
| 22 | 16601198 | rs11542850 | BC009197 | HIT000034193 | 283,+ | Cag/Tag | Q50* | PTC | AA->TER | Yes |
| 22 | 17216907 | rs5742571 | ENST00000292697 | HIT000113618 | 607,+ | Cag/Tag | Q203* | PTC | NA | No |
| 22 | 17886479 | rs885985 | AK124019 | HIT000043892 | 1170,- | Cag/Tag | Q37* | PTC | TER->AA | No |

| Chr | Position | SNP ID | Accession no. | H-Inv ID | cDNA Pos. | Codon change | AA change | Effect | Inferred direction | NMD |
| --- | --- | --- | --- | --- | --- | --- | --- | --- | --- | --- |
| 22 | 18448053 | rs2106143 | BC037564 | HIT000094888 | 392,+ | Gag/Tag | E5* | PTC | AA->TER | Yes |
| 22 | 19370205 | rs5760036 | ENST00000339055 | HIT000131685 | 694,+ | Cga/Tga | R232* | PTC | AA->TER | Yes |
| 22 | 19681560 | rs4822808 | CR597525 | HIT000276720 | 832,+ | Caa/Taa | Q90* | PTC | AA->TER | Yes |
| 22 | 20705447 | rs2582875 | BC089413 | HIT000266256 | 304,- | Cag/Tag | Q98* | PTC | AA->TER | Yes |
| 22 | 20924181 | rs17851581 | CR456609 | HIT000267543 | 327,+ | Cag/Tag | Q106* | PTC | AA->TER | No |
| 22 | 20954233 | rs2877006 | AK094832 | HIT000019687 | 634,+ | tgG/tgA | W85* | PTC | AA->TER | No |
| 22 | 21745467 | rs13055536 | AK126747 | HIT000046620 | 930,+ | Gga/Tga | G194* | PTC | TER->AA | No |
| 22 | 21951829 | rs11558699 | X02596 | HIT000320935 | 2781,+ | Cag/Tag | Q765* | PTC | AA->TER | Yes |
| 22 | 24930197 | rs5761405 | AK095662 | HIT000020517 | 471,+ | tGg/tAg | W81* | PTC | AA->TER | No |
| 22 | 25392707 | rs6005125 | AK124820 | HIT000044693 | 465,- | tGg/tAg | W143* | PTC | AA->TER | Yes |
| 22 | 25923156 | rs3888463 | AL833493 | HIT000028405 | 2514,+ | Aga/Tga | R19* | PTC | AA->TER | No |
| 22 | 26725206 | rs138658 | AL834353 | HIT000029083 | 173,- | Caa/Taa | Q22* | PTC | AA->TER | No |
| 22 | 28162153 | rs16987628 | AJ010228 | HIT000244395 | 651,+ | Cga/Tga | R148* | PTC | NA | No |
| 22 | 28162438 | rs12484086 | AJ010228 | HIT000244395 | 936,+ | Cag/Tag | Q243* | PTC | AA->TER | No |
| 22 | 30338958 | rs5753728 | AB011114 | HIT000000259 | 4029,+ | taG/taC | *1212Y | Read-through | TER->AA | NA |
| 22 | 30559378 | rs28366836 | AB014545 | HIT000000370 | 2540,+ | taT/taG | Y827* | PTC | AA->TER | Yes |
| 22 | 34925258 | rs5995251 | AY014914 | HIT000083207 | 123,- | tTa/tAa | L41* | PTC | NA | Yes |
| 22 | 35745225 | rs17850508 | CR594315 | HIT000273510 | 748,+ | Cag/Tag | Q157* | PTC | AA->TER | Yes |
| 22 | 36399107 | rs4887 | CR456511 | HIT000267445 | 304,+ | tgG/tgA | W69* | PTC | AA->TER | Yes |

| Chr | Position | SNP ID | Accession no. | H-Inv ID | cDNA Pos. | Codon change | AA change | Effect | Inferred direction | NMD |
| --- | --- | --- | --- | --- | --- | --- | --- | --- | --- | --- |
| 22 | 36571909 | rs11551392 | AK056129 | HIT000012743 | 787,+ | Cag/Tag | Q112* | PTC | AA->TER | Yes |
| 22 | 36596429 | rs11551380 | AK056129 | HIT000012743 | 1570,+ | Cag/Tag | Q373* | PTC | AA->TER | Yes |
| 22 | 37894904 | rs17000818 | AK074445 | HIT000015225 | 271,- | Gga/Tga | G9* | PTC | AA->TER | No |
| 22 | 39319635 | rs28566704 | AK128172 | HIT000048045 | 234,- | Gag/Tag | E14* | PTC | AA->TER | No |
| 22 | 40180563 | rs13766 | CR604088 | HIT000283283 | 137,+ | Cag/Tag | Q43* | PTC | AA->TER | No |
| 22 | 40859098 | rs6002633 | BC037807 | HIT000094933 | 806,+ | Cga/Tga | R103* | PTC | AA->TER | No |
| 22 | 40994791 | rs1001586 | BC039496 | HIT000095760 | 667,- | Gga/Tga | G223* | PTC | AA->TER | No |
| 22 | 41844593 | rs28392636 | X89986 | HIT000324401 | 401,+ | Gaa/Taa | E23* | PTC | AA->TER | Yes |
| 22 | 41863684 | rs11538338 | AL359401 | HIT000250444 | 185,+ | Cga/Tga | R57* | PTC | AA->TER | Yes |
| 22 | 42968230 | rs16991711 | AK090953 | HIT000015820 | 1106,+ | tgG/tgA | W46* | PTC | AA->TER | No |
| 22 | 45401494 | rs5769076 | CR609594 | HIT000288789 | 669,+ | tgG/tgA | W8* | PTC | TER->AA | No |
| 22 | 45628603 | rs5769241 | BC029897 | HIT000040917 | 1150,+ | Gag/Tag | E339* | PTC | NA | Yes |
| 22 | 48338906 | rs763128 | AK124962 | HIT000044835 | 1418,- | Cga/Tga | R139* | PTC | NA | No |
| 22 | 49109585 | rs28472552 | AK097210 | HIT000022065 | 1235,+ | tgG/tgA | W34* | PTC | NA | No |
| 22 | 49191231 | rs1063900 | BC087612 | HIT000266154 | 2198,+ | tgG/tgA | W676* | PTC | AA->TER | Yes |
| X | 13441336 | rs5935650 | AK097032 | HIT000021887 | 817,+ | tGa/tTa | *218L | Read-through | AA->TER | NA |
| X | 18403022 | rs17857094 | AY217744 | HIT000085331 | 2715,+ | Caa/Taa | Q832* | PTC | AA->TER | Yes |
| X | 19688837 | rs6654074 | BC047938 | HIT000098847 | 853,- | Cga/Tga | R34* | PTC | AA->TER | No |
| X | 19903992 | rs6527963 | CR610950 | HIT000290145 | 1346,+ | tgG/tgA | W7* | PTC | NA | No |

| Chr | Position | SNP ID | Accession no. | H-Inv ID | cDNA Pos. | Codon change | AA change | Effect | Inferred direction | NMD |
| --- | --- | --- | --- | --- | --- | --- | --- | --- | --- | --- |
| X | 23445348 | rs12711 | BC016770 | HIT000037681 | 61,+ | Gag/Tag | E2* | PTC | AA->TER | Yes |
| X | 30849286 | rs7057057 | AF285592 | HIT000076278 | 545,- | Gag/Tag | E148* | PTC | AA->TER | No |
| X | 35580784 | rs4829392 | ENST00000340777 | HIT000133407 | 814,+ | Cga/Tga | R272* | PTC | AA->TER | No |
| X | 36788868 | rs28756702 | AK125992 | HIT000045865 | 2502,+ | Aaa/Taa | K834* | PTC | AA->TER | No |
| X | 38923191 | rs5963635 | AK093678 | HIT000018545 | 483,- | Cga/Tga | R76* | PTC | AA->TER | Yes |
| X | 43412645 | rs12850496 | M69177 | HIT000196220 | 676,- | tCg/tAg | S200* | PTC | AA->TER | Yes |
| X | 46088676 | rs2478057 | AK000351 | HIT000002826 | 640,+ | tGg/tAg | W159* | PTC | TER->AA | No |
| X | 46758350 | rs10530 | AY359056 | HIT000253100 | 786,- | tgG/tgA | W85* | PTC | AA->TER | Yes |
| X | 46792194 | rs12852407 | BC008733 | HIT000033931 | 996,+ | taC/taA | Y206* | PTC | AA->TER | Yes |
| X | 47128385 | rs1052251 | ENST00000327701 | HIT000125139 | 752,+ | tTa/tGa | L251* | PTC | AA->TER | No |
| X | 47185591 | rs1050133 | BC002466 | HIT000030915 | 1556,+ | Cag/Tag | Q489* | PTC | AA->TER | Yes |
| X | 47202230 | rs17850165 | BC000866 | HIT000029962 | 655,+ | tgG/tgA | W170* | PTC | AA->TER | No |
| X | 47414300 | rs723119 | ENST00000338709 | HIT000131339 | 127,- | Cag/Tag | Q43* | PTC | AA->TER | Yes |
| X | 47890624 | rs5906622 | D49356 | HIT000101506 | 432,- | taC/taG | Y13* | PTC | AA->TER | Yes |
| X | 48169505 | rs28622886 | AK055137 | HIT000011751 | 1884,- | Tga/Gga | *150G | Read-through | NA | NA |
| X | 48888231 | rs1055983 | AK023038 | HIT000006312 | 370,- | taT/taG | Y13* | PTC | AA->TER | No |
| X | 56646937 | rs11088 | ENST00000334881 | HIT000129226 | 95,+ | tTa/tAa | L32* | PTC | AA->TER | No |
| X | 71279056 | rs11545413 | M58458 | HIT000195867 | 88,+ | tGg/tAg | W18* | PTC | AA->TER | Yes |
| X | 72216009 | rs2362986 | AB027013 | HIT000058879 | 1697,+ | tgT/tgA | C447* | PTC | AA->TER | No |

| Chr | Position | SNP ID | Accession no. | H-Inv ID | cDNA Pos. | Codon change | AA change | Effect | Inferred direction | NMD |
| --- | --- | --- | --- | --- | --- | --- | --- | --- | --- | --- |
| X | 73271946 | rs1811177 | AK128300 | HIT000048173 | 2710,- | Aag/Tag | K71* | PTC | NA | No |
| X | 74585873 | rs12853363 | ENST00000333790 | HIT000128557 | 243,+ | taT/taG | Y81* | PTC | AA->TER | No |
| X | 74787550 | rs1343879 | AK094624 | HIT000019479 | 543,- | Gag/Tag | E120* | PTC | AA->TER | No |
| X | 79991770 | rs12556157 | CR617135 | HIT000296330 | 698,+ | tGg/tAg | W178* | PTC | AA->TER | No |
| X | 91174833 | rs6615327 | ENST00000332564 | HIT000127862 | 542,- | tGg/tAg | W176* | PTC | NA | No |
| X | 95977698 | rs20382 | Y15909 | HIT000325911 | 1199,+ | tgC/tgA | C283* | PTC | AA->TER | Yes |
| X | 102124272 | rs11550088 | AF237783 | HIT000074756 | 247,+ | Caa/Taa | Q26* | PTC | AA->TER | No |
| X | 102277532 | rs3180254 | BC015794 | HIT000037328 | 502,+ | taT/taA | Y102* | PTC | AA->TER | No |
| X | 102738052 | rs2143828 | CR623631 | HIT000302826 | 415,- | Gaa/Taa | E17* | PTC | AA->TER | No |
| X | 103100905 | rs2301384 | AK093522 | HIT000018389 | 245,- | Cag/Tag | Q82* | PTC | NA | Yes |
| X | 103301056 | rs9803338 | BC053599 | HIT000053934 | 1277,- | Taa/Caa | *407Q | Read-through | AA->TER | NA |
| X | 109396099 | rs11265 | ENST00000255500 | HIT000106827 | 184,+ | Cag/Tag | Q62* | PTC | AA->TER | No |
| X | 119059687 | rs6645626 | AK123976 | HIT000043849 | 884,+ | Cga/Tga | R47* | PTC | NA | No |
| X | 119908487 | rs10657 | BC050732 | HIT000053579 | 1491,+ | Gag/Tag | E472* | PTC | AA->TER | No |
| X | 128789119 | rs1055033 | BC009649 | HIT000034489 | 2344,+ | tgA/tgT | *772C | Read-through | TER->AA | NA |
| X | 135479757 | rs17855660 | BC039856 | HIT000052272 | 2539,- | Gaa/Taa | E693* | PTC | AA->TER | Yes |
| X | 135681992 | rs16978599 | BC006550 | HIT000032918 | 1151,+ | taC/taA | Y335* | PTC | NA | No |
| X | 142692988 | rs237520 | ENST00000218188 | HIT000103289 | 266,+ | tTa/tGa | L89* | PTC | NA | No |
| X | 144417360 | rs1727458 | ENST00000332917 | HIT000128070 | 85,- | Gaa/Taa | E15* | PTC | AA->TER | Yes |

| Chr | Position | SNP ID | Accession no. | H-Inv ID | cDNA Pos. | Codon change | AA change | Effect | Inferred direction | NMD |
| --- | --- | --- | --- | --- | --- | --- | --- | --- | --- | --- |
| X | 144418436 | rs12855015 | ENST00000332917 | HIT000128070 | 1063,+ | Gag/Tag | E341* | PTC | AA->TER | Yes |
| X | 152481514 | rs4065272 | BC012355 | HIT000035775 | 1951,+ | Tga/Gga | *636G | Read-through | TER->AA | NA |
| X | 152488395 | rs12853024 | AK125631 | HIT000045504 | 935,- | Gaa/Taa | E273* | PTC | AA->TER | Yes |
| X | 152817859 | rs5945388 | X99687 | HIT000325243 | 112,+ | Gaa/Taa | E13* | PTC | AA->TER | No |
| X | 153114421 | rs743546 | X53416 | HIT000321833 | 1793,+ | Gag/Tag | E541* | PTC | AA->TER | Yes |
| X | 153557941 | rs2853347 | AF067008 | HIT000065531 | 287,+ | tCa/tAa | S76* | PTC | AA->TER | Yes |
| Y | 5250138 | rs2558170 | ENST00000312288 | HIT000119328 | 196,- | Cga/Tga | R66* | PTC | NA | No |
| Y | 10621867 | rs4018102 | ENST00000341125 | HIT000133755 | 1321,- | Aag/Tag | K441* | PTC | NA | No |
| Y | 10623095 | rs382855 | ENST00000341125 | HIT000133755 | 150,+ | taT/taA | Y50* | PTC | NA | Yes |
